# Medicinal plants of Sabah (North Borneo): lest we forget

**DOI:** 10.1080/13880209.2025.2487557

**Published:** 2025-05-02

**Authors:** Carynn Tanbuda, Mazdida Sulaiman, Pauline Yong Pau Lin, Nor Azizun Rusdi, Jaya Sathiya Seelan, Ng Shean Yeaw, Fiffy Hasnidah Saikim, Mogana Rajagopal, Nicholas Pang Tze Ping, Melanie Martos Garcia, Jhonnel Villegas, Shari Jeffri, Veeranoot Nissapatorn, Mark S. Butler, Christophe Wiart

**Affiliations:** a Institute for Tropical Biology and Conservation, Universiti Malaysia Sabah, Jalan UMS, Kota Kinabalu, Sabah, Malaysia; b Department of Chemistry, Faculty of Science, Universiti Malaya, Kuala Lumpur, Malaysia; c Unit for Ethnography Research and Development, Universiti Malaysia Sabah, Jalan UMS, Kota Kinabalu, Sabah, Malaysia; d Faculty of Pharmaceutical Sciences, UCSI University, Cheras, Kuala Lumpur, Malaysia; e Faculty of Medicine, Universiti Malaysia Sabah, Jalan UMS, Kota Kinabalu, Sabah, Malaysia; f College of Health Sciences, Mapua Malayan College Mindanao, Matina, Davao, Philippines; g Faculty of Education and Teacher Training, Davao Oriental State University, Mati, Davao Oriental, Philippines; h Sabah Society, Kota Kinabalu, Sabah, Malaysia; i School of Allied Health Sciences, Walailak University, Nakhon Si Thammarat, Thailand; j MSBChem Consulting, Brisbane, QLD, Australia

**Keywords:** Austronesians, drug discovery, ethnopharmacology, medicinal plants, North Borneo, Sabah

## Abstract

**Context:**

The discovery of plants and bioactive compounds with the potential to become botanical or pharmaceutical drugs remains a cornerstone of drug innovation. Many of these valuable molecules originate from traditional botanical pharmacopeias, repositories of centuries-old knowledge that are often underappreciated in modern research.

**Objective:**

This review highlights the medicinal plants identified in Sabah from 1922 to 2024, analyzing their taxonomical distribution, uses, utilization among ethnic groups, and their potential for clinical uses.

**Methods:**

The data for this review were gathered from Google Scholar, PubMed, ScienceDirect, Web of Science, PubMed, the Internet Archive, and Google Books. A keyword combination of “Medicinal” and “Plants” and “Sabah” yielded 21,700 results. Each result was examined, and articles that did not contain information relevant to the topic or came from non-peer-reviewed journals were excluded. Each of the remaining 87 selected articles was critically reviewed to extract pertinent information.

**Results:**

A review of the available data indicates that 696 plant species are used in Sabah, including 412 angiosperms. These plants are primarily utilized to treat diseases or symptoms related to infections, digestive issues, injuries, and pains. Notably, 156 species employed by local Sabahan Dusunic, Murutic, and Kelabit ethnic groups remain unstudied in terms of their phytochemical and pharmacological properties, highlighting their potential for further investigation.

**Conclusion:**

Sabah’s medicinal plants offer tremendous potential for discovering natural products of therapeutic value.

## Introduction

The ancient Greeks and Romans do not appear to have been aware of the island of Borneo, despite their trade networks extended to India, Sri Lanka, and parts of Southeast Asia. In the seventh century, it became part of the Buddhist Srivijaya Empire, and later fell under the control of the Hindu Javanese Majapahit Empire in the thirteenth century (Kaur 2016). By the fifteenth century, the Sultan of Brunei ruled over a territory that encompassed Borneo and parts of the Philippines. During this period, the Sultanate of Brunei was a vassal of the Chinese Ming dynasty, engaging in trade that included the exchange of camphor from *Dryobalanops aromatica* C.F. Gaertn. (Dipterocarpaceae) and other forest products, as well as bird’s nests for ceramics, gold, silver, and silk (De Vienne 2015; Gin 2015). Antonio Pigafetta, the chronicler of Magellan’s expedition, recorded the presentation of a “pot of betel leaves and areca nuts” during his 1521 visit to the Sultan of Brunei (Wright 1966). In 1877, the Sultan of Brunei leased the northeastern part of Borneo to the British North Borneo Company, which was administered as an independent enclave under British protection. During this period, quantities of dammar from *Shorea leprosula* Miq. (Dipterocarpaceae) and gutta percha from *Palaquium leiocarpum* Boerl. (Sapotaceae) were exported. Other products of economic importance at that time were the latex of "jelutong" from *Dyera costulata* Hook.f. (Apocynaceae) and from plants of the genus *Alstonia* R.Br. (1810) (Apocynaceae) (Rutter 1922). After World War II, North Borneo’s pre-war independence was not reinstated, and it became a British colony. On 16 September 1963, it joined Malaysia, leading to significant changes, including its renaming to Sabah (Leong 2009; Peng et al. 2022).

The origin of the word "Sabah" remains a subject of debate among linguists (Maxwell 1981; Ken 2015). According to Sabahan historian Shari Jeffri, this term might come from a Bruneian word describing Brunei’s northern territory and an Arabic word meaning abundance or land of abundance (personal communication). Sabah is located at 5° North latitude and 117° East longitude, covering an area of 73,904 km^2^, and lies south of the Pacific typhoon belt. Its topography features mountainous regions in the north and west, including Mount Kinabalu, while the eastern part consists of plains that were once entirely covered by primary tropical rainforest. The region’s rich botanical diversity garnered the admiration of colonial administrators such as Hugh Low (Roth and Low 1896) and Evans (1922), the latter noting that there were "so many species that even a botanist who had resided for years in the country would have difficulty in identifying them."

European and North American botanists have studied Sabah’s flora since the late nineteenth century, discovering numerous new species. Amongst the notable botanists are Odoardo Beccari (1843–1920), Henry Nicholas Ridley (1855–1956), Otto Stapf (1857–1933), Adolph Daniel Edward Elmer (1870–1942), Elmer Drew Merrill (1876–1956), Edred John Henry Corner (1906–1996), Benjamin Clemence Stone (1933–1994), John Dransfield (1945–), and John Homer Beaman (1929–2015) (De Wit 1948; Stone 1980; Beaman 1998). Beaman was deeply impressed by Sabah’s botanical richness, stating, “in terms of species per unit area, Mount Kinabalu must have one of the most diverse floras on earth” (Poulsen et al. 2000). Another distinctive feature of Sabah, particularly the Mount Kinabalu area, is the presence of ultramafic outcrops, which promote high levels of endemism (Van der Ent 2011). However, following Sabah’s integration into Malaysia, this remarkable botanical wealth was nearly destroyed to make way for oil palm plantations in the 1980s and 1990s (Collins et al. 1991; Gunggut et al. 2014; Sodhi et al. 2004).

Sabah is home to a mosaic of ethnic groups principally and linguistically Austronesians and consisting of Butungs, Javanese stock (Jawa), and North-Western Austronesian stock (Kroeger 1986; Kroeger 1991). The North-Western Austronesian stock includes the members of the Kelabit group (Lundayehs), Palawanic group (Bonggi-Molbogs), Danau group (Illanuns), Suluks (Tausugs), Bugis, Ida’anic group (Ida’ans), Malayic group (Bruneis or Kedayans, Sea Dayaks or Ibans, Cocos Malays, Bajau group (East Coast Bajaus including Kagayans and Ubians, West Coast Bajaus or Sama Bajaus), and the Bornean linguistic group. Within the Bornean linguistic group are the Dusunic sub-group (Bisayas, Dumpas, Dusuns, Kadazans, Kimaragangs, Kuijaus, Labuks, Lotuds, Papars, Rungus, Sonsogons, Tatanas, Tobilungs, and others) (Appell 1968; Pugh-Kitingan 2015), the Murutic sub-group (Bookans, Ganas, Kalabakans, Keningaus, Muruts, Paluans, Sembakungs, Serundungs, Tagals, Timugons, and others), the Paitanic sub-group (Abai Sungais, Kalabuans, Lingkabaus, Lobus, Paitans, Rumanaus, Sinabus, Subpans, and Upper Kinabatangans often collectively called Orang Sungai), and the Tidung sub-group (Tidungs). Other minority groups in Sabah include descendants of Hakka Chinese immigrants, who were brought to North Borneo by Christian missions and tobacco companies, as well as a small number of Indians. The Chinese community in Sabah relies primarily on medicinal plants associated with traditional Chinese medicine, which are not covered in this review. The last remaining Negrito groups in Sabah are now extinct (Williams 1968; Oppenheimer 2009).

Until the advent of modern medicine, the local inhabitants of Sabah, particularly those from the Dusunic and Murutic communities, faced significant health challenges, including anemia, intestinal worms, malaria, poliomyelitis, protein malnutrition, smallpox, and tuberculosis (Koblenzer 1958). Even though healthcare advances have been made in Sabah, certain areas remain underserved by medical services. In these remote regions, medicinal plants continue to be primary means of treating diseases, especially in low-income communities. For instance, malaria remains a major health issue in rural Sabah (Ramdzan et al. 2020). Historical records of medicinal plant use in Sabah date back to Ruttler (1922), who documented the use of camphor and gambier. Evans (1922) described the beliefs and rituals surrounding the sacred “lempada” trees in the genus *Tabernaemontana* Plum. ex L. (1753) (Apocynaceae) of the Dusun people. Among the earliest phytochemical studies of Sabah’s plants are those by Arthur (1954) and Goh et al. (1997). More recent studies by researchers of the Malay Peninsula (Kam et al. 1999) led to the identification of a series of monoterpene indole alkaloids, among which kopsiflorine, which enhanced the toxicity of vincristine against multidrug-resistant human epidermoid carcinoma (KB) cells (Rho et al. 1999).

The treatment of certain cancers, including drug-resistant cancers, the threat of new viral pandemics, and the emergence of bacteria and fungi resistant to antibiotics and antifungals require the urgent discovery of new therapeutic molecules. Such original drugs may be found in the plants used in traditional botanical pharmacopeias (Cox and Balick 1994), and particularly in plants from primary tropical forests (Soejarto and Farnsworth 1989). For example, research conducted in the 1990s on *Calophyllum teysmannii* var. *inophylloide* (King) P.F. Stevens (Clusiaceae), in Sarawak, Malaysia, located in northern Borneo, led to the discovery of a novel pyranocoumarin, calanolide A, with showed potential therapeutic applications for AIDS treatment (Kashman et al. 1992; Fuller et al. 1994). Another biologically active compound of pharmaceutical interest from the Sarawak rainforest is silvestrol, a flavonolignan isolated from *Aglaia silvestris* Merr. (Meliaceae), which has potential for the treatment of viral infections and cancer (Schulz et al. 2021; Sharapov et al. 2023).

In this context, this review addresses the following points: taxonomic distribution, uses, specificity of ethnic groups, side effects, therapeutic applications, and future perspectives. This review aims to facilitate the discovery of molecules of clinical value, over-the-counter herbal remedies, nutraceuticals, or cosmetics from the Sabah’s medicinal plants.

## Methods

All data for this review were sourced from Google Scholar, PubMed, ScienceDirect, Web of Science, Internet Archive, and Google Books. A search using the keywords "Medicinal," "Plants," and "Sabah" returned 21,700 results. The inclusion criteria focused on articles, conference proceedings, and books specifically addressing the medicinal plants of Sabah. Articles, conference proceedings, and books related to medicinal plants from regions or countries outside Sabah, as well as non-English works or those not peer-reviewed, were excluded. Each of the 87 selected articles was critically reviewed to extract relevant information.

## Taxonomical distribution

### General observation

Analysis of the available evidence revealed the use of 696 medicinal plant species, representing 136 families. This includes five lycophytes (0.7%), 29 monilophytes (4.1%), six gymnosperms (0.8%), and 656 angiosperms (94.2%). Among the angiosperms, there are 218 basal angiosperms (33.3%), 273 core angiosperms (41.6%), and 165 upper angiosperms (25.1%). Three species of lycophytes, six of monilophytes, two of gymnosperms, 61 basal angiosperms, 58 of core angiosperms, and 42 of upper angiosperms remain incompletely identified. Additionally, one fern and a species in the family Poaceae are also unidentified. It is important to note that the botanical identifications provided by local experts may not always be definitive.

### Lycophytes

Five species of mosses are in the subclass Lycopodiidae from two families are medicinal in Sabah: three in the Lycopodiaceae and two in the Selaginellaceae. None of the five species identified are endemic to Sabah (Table 1).

**Table 1.** Medicinal plants of Sabah (lycophytes).

Subclass Order Family	Genus, species, authority	Symptoms/diseases (ethnic group)	Local names	References
**Lycopodiidae Bek. (1862) (Lycophytes)**				
**Lycopodiales DC. ex Bercht. & J. Presl (1820)**				
Lycopodiaceae P. Beauv. ex Mirb. (1802)	*Lycopodium cernuum* L.	Canker sores, fever, hypertension (Dusun)	Gogor	Voeks and bin Nyawa (2006), Wiart (2024)
		Asthma, chest pain, coughs (Brunei)	Tapok tapokan	Mohiddin et al. (1992)
	*Lycopodium phlegmaria* L.	Hair loss (Brunei)	Sari gading bini	Mohiddin et al. (1992)
	*Lycopodium* sp.	Fever (Dusun)	Rongilut	Kulip et al. (2005)
**Selaginellales Prantl (1854)**				
Selaginellaceae Willk. (1854)	*Selaginella argentea* (Wall. ex Hook. & Grev.) Spring^σ†Δ^	Asthma, body aches, fever, headaches (Murut)	Sondotnulogo	Ahmad and Raji (1992), Kulip (2003a)
		Medicinal (Bonggi-Molbog)	Ipa ipa puteh	Lin (2022)
	*Selaginella plana* (Desv. ex Poir.) Hieron^σϕµ^	Fever, headaches (Murut)		Ahmad and Holdsworth (1994)

β: Borneo; σ: Sundaland; ϕ: Wallacea; µ: Sahuland; †: no phytochemical and/or pharmacological study; Δ: worthy of further investigation

### Monilophytes

Twenty-nine monilophytes species, representing 15 families, are medicinal in Sabah, 19 of which in the subclass Polypodiidae and out of these four species in the genus Polypodiales Link (1833) and 11 species are in the genus Schizaeales Schimp. (1869) of which four species in the Lygodiaceae. None of the species identified are endemic to Sabah (Table 2).

**Table 2.** Medicinal plants of Sabah (monilophytes).

Subclass Order Family	Genus, species, authority	Symptoms/diseases (ethnic group)	Local name	References
**Ophioglossidae Klinge (1832)**				
**Ophioglossales Link. (1833)**				
Ophioglossaceae Martinov (1820)	*Helminthostachys zeylanica* (L.) Hook.	Cancer, wounds (Lundayeh)	Pajerok	Kulip et al. (2000), Kulip (2007a)
		Postpartum (Bonggi-Mogbol)	Onitug	Lin (2022)
		Medicinal food (Dusun)	aruk Aruk	Noweg et al. (2004)
**Maratiidae Klinge (1882)**				
**Marattiales Link. (1833)**				
Marattiaceae Kaulf. (1824)	*Angiopteris evecta* (G. Forst.) Hoffm.	Diarrhea, swelling (Dusun)	Paku tiou	Wiart (2024)
	*Angiopteris* sp.	Medicinal	Pako	Kodoh (2005)
**Equiseridae Warm. (1883)**				
**Equisetales DC. ex Bercht. & J. Presl** **(1820)**				
Equisetaceae Michx. ex DC. (1804)	*Equisetum ramosissimum* Desf.	Fever (Dusun)	Langod	Wiart (2024)
**Polypodiidae Cronquist, Takht. & W. Zimm. (1966)**				
**Blechnales Pic. Sem. ex Reveal (1993)**				
Blechnaceae Newmann (1844)	*Blechnum orientale* L.	Bacterial skin infection, insect stings, flatulence, headaches, wounds, ulcers (Dusun)	Dungau	Ahmad and Holdsworth (2003), Benggon (2008)
		Bacterial skin infection, insect stings, flatulence, headaches, wounds, ulcers (Kadazan)	Dungau	Ahmad and Holdsworth (2003), Benggon (2008)
	*Stenochlaena palustris* (Burm. f.) Bedd.	Medicinal food, postpartum, fever, skin diseases (Dusun)	Lambiding	Noweg et al. (2003), (Maid et al. (2017)
		Postpartum, fever, skin diseases (Kadazan)	Lambiding	Noweg et al. (2003)
**Gleicheniales Link** **(1825)**				
Gleicheniaceae C. Presl (1825)	*Dicranopteris* sp.	Sore eyes (Dusun)	Kawang kawang	Kulip et al. (2005)
		Sore eyes (Kadazan)	Kawang kawang	Kulip et al. (2005)
	*Gleichenia truncata* (Willd.) Sprain^†^	Sore eyes (Dusun)	Laputong	Kulip (2014)
**Polypodiales Link (1833)**				
Athyriaceae Alston (1956)	*Diplazium cordifolium* Bl.^† Δ^	Cold, fever (Bajau)	Giman	Wiart (2024)
	*Diplazium esculentum* (Retz.) Sw.	Medicinal food (Dusun)	Pakis	Noweg et al. (2003), Maid et al. (2017)
Polypodiaceae Link J. Presl & C. Presl (1822)	*Drymoglossum piloselloides* (L.) C. Presl^†Δ^	Diuretic, gallstones, hypertension (Brunei)	Sisik naga	Mahmud and Razali (2016)
	*Drynaria roosii* Nakaike	Prenatal care (Bonggi-Molbog)	Kobkab	Lin (2022)
	*Drynaria sparsisora* (Desv.) T. Moore^Δ^	Asthma, heart diseases (Dusun)	Tapako	Wiart (2024)
		Asthma, heart diseases (Kadazan)	Tapako	Wiart (2024)
	*Pyrrosia lanceolata* (L.) Fawr.^†^	Pancreatitis (Lundayeh)	Ubat alib	Kulip et al. (2000)
**Schizaeales Schimp.** **(1869)**				
Cytheaceae Kaulf. (1827)	*Cibotium* sp.	Bacterial skin infection, snakebites (Dusun)	Paku	Kulip et al. (2005)
		Bacterial skin infection, snakebites (Kadazan)	Paku	Kulip et al. (2005)
Lygodiaceae M. Roem. (1840)	*Lygodium circinnatum* Sw.^†Δ^	Venereal diseases (Lundayeh)	Waratang	Kulip et al. (2000)
		Womb diseases (Dusun)	Taribu mianai	Voeks and bin Nyawa (2006)
	*Lygodium flexuosum* (L.) Sw.	Fever, haemoptysis (Bajau)	Ribu besar	Mojiol et al. (2010)
	*Lygodium microphyllum* (Cav.) R. Br.^†^	Womb diseases (Dusun)	Taribu indu	Voeks and bin Nyawa (2006)
	*Lygodium salicifolium* C. Presl^†Δ^	Chickenpox, smallpox (Lundayeh)	Ubat amur	Kulip et al. (2000)
Nephrolepidaceae Pic. Serm. (1975)	*Nephrolepis acutifolia* (Desv.) Christ	Medicinal food (Dusun)	Paku puteh	Noweg et al. (2003)
	*Nephrolepis dicksonioides* Christ^πϕµ†^	Chills, itchiness (Dusun)	Ngkubuk	Voeks and bin Nyawa (2006)
	*Nephrolepis* sp.	Headaches (Dusun)	Monumpuru	Andersen et al. (2003)
		Headaches (Kadazan)	Monumpuru	Andersen et al. (2003)
Pteridaceae E.D.M. Kirchn. (1831)	*Acrostichum aureum* L.	Medicinal food (Dusun)	Paku besar	Noweg et al. (2003)
	*Pteris* sp.	Fatigue (Dusun)	–	Voeks and Nyawa (2001)
Schizaeaceae Kaulf. (1827)	*Schizaea dichotoma* (L.) Sw.^†^	Fatigue (Dusun)	Pitagar paying	Voeks and Nyawa (2001)
Thelypteridaceae Ching ex Pic. Serm. (1970)	*Cyclosorus aridus* (D.Don) Ching^†^	Medicinal food (Dusun)	Paku	Noweg (2003)
	*Pneumatopteris* sp.	Flatulence, postpartum (Dusun)	Menampun	Kulip et al. (2000)
		Flatulence, postpartum (Kadazan)	Menampun	Kulip et al. (2000)
	*Pronephrium asperum* (C. Presl) Holttum^σπϕµ†^	Medicinal (Murut)	Ingkakahas	Kulip (2003a)
Incognita	Incognita	Medicinal (Bonggi-Molbog)	Ipah ipah merah	Lin (2022)

β: Borneo; σ: Sundaland; ϕ: Wallacea; π: Philippines; µ: Sahuland; †: no phytochemical and/or pharmacological study; Δ: worthy of further investigation.

### Gymnosperms

Six gymnosperm species have been so far identified as medicinal in Sabah represented by 3 families, half of which are in the family Gnetaceae (Table 3). None are endemic, while one species remains unidentified.

**Table 3.** Medicinal plants of Sabah (gymnosperms).

**Subclass Order** Family	Genus, species, authority	Symptoms/diseases (ethnic group)	Local name	References
**Cycadidae Pax (1894)**				
**Cycadales Pers. Ex Bercht. & J. Presl (1820)**				
Cycadaceae Pers. (1807)	*Cycas revoluta* Thunb.	Bleedings, blood circulation, bruises, cancer, coughs, gastritis, hypertension, liver pain, neuralgia, sprains, rheumatism (Bajau)	Paku laut	Lee et al. (2015)
**Pinidae Cronquist, Takht. & W. Zimm. (1966)**				
**Auraucariales Gorozh. (1904)**				
Auraucariaceae Henkel & W. Hochst. (1865)	*Agathis borneensis* Warb^σ^	Medicinal	Rajah kayu	Foo (2018)
**Pinales Gorozh (1904)**				
Pinaceae Spreng. & Rudolphi (1830)	*Pinus* sp.	Fever (Bajau)	Nanas batu	Foo et al. (2016)
**Gnetidae Pax (1894)**				
**Gnetales Blume (1835)**				
Gnetaceae Blume (1833)	*Gnetum gnemon* L.	Fatigue (Dusun)	Bagu	Voeks and bin Nyawa (2006)
	*Gnetum macrostachyum* Hook.f.^σµΔ^	Fatigue, postpartum (Brunei)	Kokos	Kulip (1997)
	*Gnetum* sp.	Medicinal (Bajau)	Lautan seribu	Kodoh et al. (2009)

β: Borneo; σ: Sundaland; ϕ: Wallacea; π: Philippines; µ: Sahuland; Δ: worthy of further investigation.

### Basal angiosperms

There are 218 medicinal species in this clade represented by 53 families. Most of these plants are in the monocots with 158 species (72.4%) (Table 4). The three principal families of medicinal Monocots are the Zingiberaceae (53 species), the Poaceae (26 species), and the Arecaceae (22 species). Out of 49 magnoliid species, 24 are in the Lauraceae and 23 are in the Annonaceae. Twenty species (12.8%) are endemic to Borneo of which five are in the Annonaceae, four in the Musaceae and in the Zingiberaceae, and three in the Poaceae.

**Table 4.** Medicinal plants of Sabah (basal angiosperms).

**CLADE Subclass Order** Family	Genus, species, authority	Symptoms/diseases (ethnic group)	Local name	References
**PROTOMAGNOLIIDS**				
**Austrobaileyanae Doweld ex M.W. Chase & Reveal (2009)**				
**Austrobaileyales Takht. ex Reveal (1992)**				
Chloranthaceae R. Brown ex Sims (1820)	*Chloranthus officinalis* (Buch.-Ham.) Verdc.	Bleeding (Dusun)	Totol	Andersen et al. (2003)
		Bleeding (Kadazan)	Totol	Andersen et al. (2003)
	*Chloranthus* sp.	Skin diseases, body aches, internal injuries (Dusun)	kosup	Wiart (2024)
		Skin diseases, body aches, Internal injuries (Kadazan)	Kosup	Wiart (2024)
Schisandraceae Blume (1830)	*Kadsura borneensis* A.C. Sm^β†Δ^	Cramps (Lundayeh)	Putu urat	Kulip et al. (2000)
	*Kadsura lanceolata* King^σϕ†Δ^	Bacterial skin infection, swelling (Dusun)	Topis	Wiart (2024)
**MAGNOLIIDS**				
**Magnolianae Takht. (1967)**				
**Piperales Bercht. & C. Presl (1820)**				
Aristolochiaceae Juss. (1789)	*Aristolochia foveolata* Merr.^βσ^	Blow-gun darts poison antidote (Murut)	Tabar kedayan	Wiart (2024)
	*Aristolochia minutiflora* Ridl. ex Gamble^σ†Δ^	Diarrhea, vomiting (Lundayeh)	Lapad talang	Kulip et al. (2000)
	*Aristolochia papillifolia* Ding Hou^β†Δ^	Diarrhea, hepatitis, jaundice, pancreatitis, poison antidote (Murut)	Babas lontong	Kulip (2003a)
Piperaceae Giseke (1792)	*Peperomia pellucida* (L.) Kunth	Bacterial skin infection, blisters, coughs, diabetes, flu, hypertension rheumatism	Ketumpangan air	Mahmud and Razali (2016)
		sore eyes, (Bajau)	longsima	Foo et al. (2016)
		Sore eyes, fever, stomach aches (Bisaya)		Mojiol et al. (2010)
	*Piper betle* L.	Asthma, halitosis, bacterial skin infection, coughs, diabetes, nosebleeds, gout, hypertension, itchiness, stomach aches, toothaches, ulcers (Bajau)	Sirih	Kulip (2003a), Ahmad and Ismail (2003), Kodoh et al. (2014), Kodoh et al. (2017), Mahali et al. (2023)
		Bone pain, coughs, diabetes, halitosis, nosebleeds, scabies, wounds (Dusun)	Daing	Kulip (2003a)
		Hypertension (Rungus)		Kodoh et al. (2014)
		Fever (Brunei)	Daun sireh	Kodoh et al. (2014)
		Toothaches (Illanun)		Wiart (2024)
		Bacterial skin infection, body odor, coughs, diabetes, flatulence, halitosis, insect stings, nosebleeds, scabies, skin diseases, wounds (Kadazan)	Daing	Wiart (2024)
			Sirih hutan	
	*Piper caducibracteum* C. DC^βϕ†Δ^	Earache, menorrhagia, itchiness (Brunei)		Mahmud and Razali (2016)
	*Piper caninum* Bl.	Medicinal (Murut)	Kimput pilot	Wiart (2024)
	*Piper sarmentosum* Roxb.	Coughs, flu, malaria, ringworms, toothaches (Bajau)	Kaduk	Wiart (2024)
	*Piper umbellatum* L.	Bacterial skin infection (Dusun)	Kuyoh	Kulip (2014)
	*Piper* sp.	Skin diseases, insect stings (Dusun)	Bohuton	Kulip et al. (2005)
**Laurales Juss. ex Bercht. & Presl (1820)**				
Lauraceae Juss. (1789)	*Actinodaphne* sp.	Waist pain (Dusun)	Pongulobon kusai	Kulip et al. (2005)
		Waist pain (Kadazan)		
	*Cinnamomum iners* Reinw. ex Blume	Coughs, joint pain (Dusun)	Medang teja	Wiart (2024)
		Joint pain (Kadazan)	Medang teja	Wiart (2024)
	*Cinnamomum* sp.1	Stomach aches (Dusun)	Kusun	Andersen et al. (2003)
		Stomach aches (Kadazan)	Kusun	Andersen et al. (2003)
	*Cinnamomum* sp.2	Beriberi (Dusun)	Makalabau	Kulip (1997)
		Beriberi (Kadazan)	Makalabau	Kulip (1997)
	*Cinnamomum cassia* (L.) J. Presl	Cold (Bajau)	Kayu manis	Foo et al. (2016)
	*Eusideroxylon zwageri* Teijsm. & Binn.^σ†Δ^	Blow-gun darts poison _(_Murut)	Belian	Kulip (2003a)
	*Lindera pipericarpa* Boerl.^σ^	Snake bites (Murut)	Laindos	Kulip (2003a)
		Flatulence (Dusun)	Laindos	Wiart (2024)
		Abdominal pain (Lundayeh)	Tanom	Kulip et al. (2000)
	*Litsea accedens* (Bl.) Boerl.^σ^	Medicinal		Wiart (2024)
	*Litsea garciae* Vidal.^βπ^	Dislocation, sprains (Murut)	Pengolaban	Wiart (2024)
		Medicinal food (Dusun)	Pengolaban	Maid et al. (2017)
	*Litsea odorifera* Valeton ^σπϕ^	Gastritis, stomach aches (Murut)	Lawing	Kulip (2003a)
		Bone pain, diarrhea, fatigue, gastritis, skin diseases, stomach aches (Dusun)		Kulip (2003b)
		Bone pain, diarrhea, fatigue, gastritis, skin diseases, stomach aches (Kadazan)		Kulip et al. (2003b)
	*Litsea umbellata* (Lour.) Merr.	Medicinal	Medang wangi hitam	Kulip et al. (2010)
	*Litsea sp*.1	Bacterial skin infection (Dusun)	Sesulang kupes	Kulip (2014)
	*Litsea sp*.2	Scabies, wounds (Dusun)	Lamou lamou	Kulip (2014)
	*Litsea sp*.3	Flatulence (Dusun)	Sileu	Kulip (2014)
	*Persea americana* Mill.	Fever (Murut)	Buah lemak	Wiart (2024)
Annonaceae Juss. (1789)	*Annona muricata* L.	Coughs, stomach aches, hypertension, nausea, fever, diarrhea (Dusun)	Hampun kapal	Kulip (2003a)
		Coughs, stomach aches, hypertension, nausea, fever, diarrhea (Kadazan)	Hampun kapal	Kulip (2003a)
		Cancer (Bajau)	Nangka Belanda	Foo et al. (2016)
		Asthma, coughs, constipation (Murut)	Lampoon tuan	Kulip (2003a)
	*Artabotrys roseus* Boerl.^β†Δ^	Medicinal (Dusun)	Gangon	Kodoh et al. (2009)
		Medicinal (Kadazan)	Gangon	Kodoh et al. (2009)
	*Desmos teysmannii* (Boerl.) Merr.^σ^	Headaches (Murut)	Molisun rumungkut	Kulip (2003a)
	*Enicosanthum* sp.	Medicinal		Kodoh et al. (2009)
	*Fissistigma fulgens* Merr.^σπ^	Fatigue, headaches, toothaches (Dusun)		Kulip (2003a)
		Fatigue, headaches, toothaches (Kadazan)		Kulip (2003a)
	*Fissistigma latifolium* (Dunal) Merr.	Abdominal pain (Dusun)	Sumbun	Kulip (1997)
		Abdominal pain (Kadazan)	Sumbun	Kulip (1997)
	*Fissistigma manubriatum* Merr.^σ^	Fatigue (Dusun)	Gagon	
		Fatigue (Kadazan)	Gagon	
	*Goniothalamus roseus* Stapf^β†Δ^	Fatigue, fever (Dusun)	Limpanas	Foo et al. (2016)
	*Goniothalamus umbrosus* J. Sinclair^σ^	Magic rituals (Dusun)	Limpanas purak	Voeks and bin Nyawa (2006)
	*Goniothalamus velutinus* Airy Shaw^β^	Magic rituals (Dusun)	Kalampanas	Voeks (2008)
	*Goniothalamus woodii* Merr^βΔ^	Magic rituals (Murut)	Tampaliu	Wiart (2024)
	*Phaeanthus ophthalmicus* (Roxb. ex G. Don) J. Sinclair ^σπϕ^	Sore eyes (Murut)	Korokos	Wiart (2024)
	*Polyalthia bullata* King^σ^	Seizures (Lundayeh)	Lapad ruai	Kulip et al. (2000)
	*Polyalthia insignis* (Hook.f.) Airy Shaw^βπ^	Coughs, thrush (Dusun)		Kulip (2003b)
		Coughs, thrush (Kadazan)		Kulip et al. (2003b)
	*Polyalthia sumatrana* (Miq.) Kurz^σ^	Medicinal		(Kulip 2010)
	*Polyalthia tenuipes* Merr.^β†Δ^	Sick children (Dusun)	Kabanking	Wiart (2024)
	*Polyalthia* sp.	Skin diseases (Dusun)	Dolipanas	
	*Uvaria cuneifolia* (Hook.f. & Thomson) L.L. Zhou^σ†Δ^	Fever (Bajau)	Kayu bibiris	Wiart (2024)
	*Uvaria grandiflora* Roxb. ex Hornem	Fever, intestinal worms, jaundice, fatigue, stomach aches (Dusun)	Potudung	Wiart (2024)
		Fever, intestinal worms, jaundice, fatigue, stomach aches (Kadazan)	Potudung	Wiart (2024)
		Stomach aches, waist pain (Murut)	Nolilitan	Kulip (2003a)
	*Uvaria sorzogonensis* C. Presl^†Δ^	Fatigue (Dusun)	Sogombong	Wiart (2024)
		Fatigue (Kadazan)	Sogombong	Wiart (2024)
	*Uvaria* sp.1	Swelling (Dusun)	Bab	Kulip et al. (2005)
	*Uvaria* sp.2	Rheumatism (Dusun)	Langad langad	Kulip (1997)
	*Xylopia dehiscens* (Blanco) Merr.^βπ†Δ^	Medicinal (Rungus)	Mizas pizas	Wiart (2024)
	*Xylopia* sp.	Swelling (Dusun)	Linsou linsou	Kulip et al. (2005)
		Swelling (Kadazan)	Linsou linsou	Kulip et al. (2005)
**MONOCOTS**				
**Lilianae Takht. (1967)**				
**Order Acorales Link (1835**)				
Acoraceae Martinov (1820)	*Acorus calamus* L.	Fever, stomach aches (Murut)	Kusul	Kulip (2003a)
		Diarrhea, gastritis, poison antidote, insect stings, fatigue, magic rituals skin diseases (Dusun)	Komburongoh	Kulip (1997), Kulip (2009), On (2016), Kulip (2014)
		Diarrhea, gastritis, poison antidote, insect stings, fatigue (Kadazan)	Komburongoh	Kulip (1997), Kulip (2009)
Araceae Juss. (1789)	*Aglaonema oblongifolium* (Roxb.) Kunth^βϕµ Δ^	Bacterial skin infection (Murut)	Pilonos	Kulip (2003a)
	*Alocasia macrorrhizos* (L.) G. Don^βϕπµ^	Itchiness (Murut)	Buntui	Kulip (2003a)
	*Alocasia* sp.1	Itchiness (Dusun)	Sisial	Wiart (2024)
		Itchiness (Kadazan)	Sisial	Wiart (2024)
	*Alocasia* sp.2	Measles (Dusun)	Tanom	Kulip (1997)
		Measles (Kadazan)	Tanom	Kulip (1997)
	*Amorphophallus prainii* Hook.f. ^†Δ^	Medicinal food (Dusun)	Undipoh	Noweg et al. (2003)
	*Amydrium medium* Nicolson^σϕπ^	Flu (Dusun)	Kulimpiau	Kulip (1997)
		Flu (Kadazan)	Kulimpiau	Kulip (1997)
		Swollen legs (Lundayeh)	Lapad bara	Kulip (1997)
	*Homalomena propinqua* Schott^σϕ Δ^	Cold (Murut)	Nyato	Wiart (2024)
	*Homalomena* sp.	Magic rituals (Dusun)	Latu	Wiart (2024)
	*Rhaphidophora korthalsii* Schott^βϕπµ^	Gout (Lundayeh)	Ubat ugut	Kulip et al. (2000)
	*Schismatoglottis* sp.1	Medicinal (Murut)	Pongongondog	Kulip (2003a)
	*Schismatoglottis* sp.2	Medicinal food (Dusun)	Dukaruk	Noweg et al. (2003)
	*Scindapsus longistipitatus* Merr. ^β†Δ^	Skin diseases (Dusun)	Timbalung lanut	Wiart (2024)
		Skin diseases (Kadazan)	Timbalung lanut	Wiart (2024)
	Scindapsus perakensis Hook.f.	Medicinal (Murut)	Pagawangan	Kulip (2003a)
	Scindapsus sp.	Swelling (Dusun)	Timbolung lolu	Wiart (2024)
		Swelling (Kadazan)	Timbolung lolu	Wiart (2024)
**Alismatales R.Br. ex Bercht. & J.Presl (1820)**				
Alismataceae Vent. (1799)	Limnocharis flava (L.) Buchenau	Medicinal food (Dusun)	Kakatong	Noweg et al. (2003)
**Asparagales Link (1829)**				
Amaryllidaceae J. St.-Hil. (1805)	*Allium ascalonicum* L.	Cancer, wounds (Lundayeh)	Bawang Siam	Wiart (2024)
		Stomach aches, headaches, Joint pain (Rungus)		Kodoh et al. (2014), Kodoh et al. (2017)
		Fever (Jawa)		Wiart (2024)
		Flatulence (Dusun)		Kodoh et al. (2014, 2017)
		Flatulence (Kadazan)		Kodoh et al. (2014, 2017)
	*Allium cepa* L.	Cancer, fever, skin diseases (Bugis)	Lasuna cellak	Laudeh and Foo (2021), Wiart (2024)
	*Allium sativum* L.	Fever (Bugis)	Lasuna puteh	Laudeh and Foo (2021), Wiart (2024)
Asparagaceae Juss. (1789)	Cordyline fruticosa (L.) A. Chev.	Flatulence (Murut)	Pipisokalaganan	Kulip (2003a)
		Postpartum (Dusun)		Kulip (2003a)
		Postpartum (Kadazan)		Kulip (2003a)
	*Cordyline* sp.	Medicinal (Dusun)	Rolok	Andersen et al. (2003)
		Medicinal (Kadazan)	Rolok	Andersen et al. (2003)
	*Dracaena elliptica* Thunb.	Fatigue (Murut)	Sipak	Kulip (2003a)
	*Dracaena umbratica* Ridl.^σ^	Medicinal (Murut)	Dolol apui	Kulip (2003a)
	*Dracaena* sp.1	Magic rituals (Dusun)	Patidong	Wiart (2024)
	*Dracaena* sp.2	Fatigue (Dusun)	Sambangun	Voeks and Nyawa (2001)
	*Sansevieria trifasciata* Prain	Earache, itchiness, toothaches (Brunei)	Lidah jin	Wiart (2024)
		Blood circulation, kidney stones, poison antidote, wounds (Bajau)		Wiart (2024)
		Kidney stones (Dusun)		Wiart (2024)
Aspholedaceae Juss. (1789)	*Aloe vera* (L.) Burm.f.	Hair loss, kidney stones, bacterial skin infection, Postpartum, wounds (Bajau)	Lidah buaya	Awang-Kanak et al. (2018)
		Dandruff, skin diseases, wounds (Bugis)	Lidah buaya	Laudeh and Foo (2021)
		Asthma, burns, sore throats, fever, wounds (Brunei)	Lidah buaya	Wiart (2024)
		Burns, itchiness, hair wash, stomach aches, wounds (Dusun)	Dihabuazo	Ahmad and Ismail (2003)
		Stomach aches, wounds (Kadazan)	Dihabuazo	Ahmad and Ismail (2003)
		Bacterial skin infection, wounds (Rungus)		Kodoh et al. (2014)
		Insect stings (Illanun)		Wiart (2024)
	*Dianella ensifolia* (L.) Redouté	Headaches, fatigue (Dusun)	Tagari	Voeks and Nyawa (2006)
Hypoxidaceae R.Br. (1814)	*Curculigo latifolia* Dryand. ex W.T. Aiton	Asthma, abdominal pain, bone pain, coughs, skin diseases (Dusun)	Tambaka	Kulip (2003b)
		coughs, skin diseases (Kadazan)	Tambaka	Kulip (2003b)
		Fever, wounds (Murut)	Tambaka	Kulip (2003a)
		Abdominal pain (Lundayeh)	Tambaka	Kulip et al. (2000)
Orchidaceae Juss. (1789)	*Bromheadia finlaysoniana* (Lindl.) Miq.^σπϕµ†Δ^	Fatigue (Dusun)		Voeks and bin Nyawa (2006)
	*Dendrobium umbellatum* Rchb. f.^ϕµ†Δ^	Medicinal (Murut)	Tingasu	Wiart (2024)
	*Epigeneium* sp.	Cold, snakebites (Dusun)	Tapako	Kulip et al. (2005)
	*Flickingeria* sp.	Snakebites (Kadazan)		Kulip et al. (2005)
**Dioscoreales R.Br. (1835)**				
Dioscoreaceae R.Br. (1810)	*Dioscorea* sp.	Fatigue (Dusun)	Kolonton aiso due	Kulip (1997)
		Fatigue (Kadazan)	Kolonton aiso due	Kulip (1997)
**Liliales Pelerb (1826)**				
Smilacaceae Vent. (1799)	*Smilax odoratissima* Bl.^†Δ^	Sore throats (Lundayeh)	Lapad makar	Kulip et al. (2000)
	*Smilax* sp.1	Back and waist pain (Dusun)	Tunda	Kulip (2014)
		Back and waist pain (Kadazan)	Tunda	Kulip (2014)
	*Smilax* sp.2	Food poisoning (Dusun)	Tongkung kowilan	Kulip et al. (2005)
		Food poisoning (Kadazan)	Tongkung kowilan	Kulip et al. (2005)
**Pandanales R.Br. ex Bercht. & J. Presl (1820)**				
Pandanaceae R.Br. (1810)	*Pandanus amaryllifolius* Roxb.	Gout (Bajau)	Pandan	Awang-Kanak et al. (2018), Mahali et al. (2023)
**Arecales Bromhead (1840)**				
Arecaceae Bercht. & J. Presl (1820)	*Areca catechu* L.	Hypertension, poison antidote, scabies, syphilis, toothaches, wounds (Dusun)	Lugus	Kulip et al. (2005), Ahmad and Ismail (2003), Awang-Kanak (2022)
		Hypertension, poison antidote, scabies, syphilis, toothaches, wounds (Kadazan)	Lugus	Kulip et al. (2005), Ahmad and Ismail (2003), Awang-Kanak (2022)
		Anemia, stomach aches, bloating, gastritis, hypertension, toothaches (Bajau)	Pinang	Ahmad and Holdsworth (1995, 2014), Foo et al. (2016), Kodoh et al. (2017)
		Fatigue, scabies, toothaches, wounds (Rungus)		Wiart (2024)
		Medicinal (Bonggi-Molbog)		Lin (2022)
	*Arenga brevipes* Becc^.σ†Δ^	Medicinal (Dusun)		Wiart (2024)
	*Arenga pinnata* (Wurmb) Merr.	Medicinal food (Dusun)	Puluk	Noweg et al. (2003)
	*Calamus* sp.1	Fever (Dusun)	Lambah	Awang-Kanak et al. (2021)
	*Calamus* sp.2	Beriberi (Kadazan)	Sarae	Kulip et al. (2005)
	*Calamus sp*.3	Fatigue (Kadazan)	Tuai bondig	Kulip et al. (2005)
	*Caryota mitis* Lour.	Lack of milk (Dusun)	Botu	Martin et al. (2002), Wiart (2024)
	*Caryota* sp.1	Lack of milk (Dusun)		Andersen et al. (2003)
	*Caryota* sp.2	Medicinal food (Dusun)	Lurung	Noweg et al. (2003)
	*Cocos nucifera* L.	Fever, hypertension, measles, Smallpox (Bajau)	Suka	Kodoh et al. (2017), Awang-Kanak (2022), Mahali et al. (2023)
		Smallpox (Sungai)		Kodoh et al. (2014)
		Diarrhea, fever, headaches, hypertension, smallpox (Dusun)	Piasau	Ahmad and Holdsworth (2003),
		Diarrhea, fever, headaches (Kadazan)	Piasau	Ahmad and Holdsworth (2003), Kodoh et al. (2014)
		Smallpox (Rungus)		Kodoh et al. (2014)
		Smallpox (Brunei)		Kodoh et al. (2014)
		Smallpox (Illanun)		Kodoh et al. (2014)
		Cancer, headaches, itchiness, mumps, poison antidote (Bugis)	Kaluku	Laudeh and Foo (2021), Wiart (2024)
		Medicinal (Bonggi-Molbog)		Lin (2022)
		Postpartum (Murut)		Muhammed and Muthu (2015)
	*Daemonorops didymophylla* Becc.^σ^	Medicinal (Dusun)	Lomu	Wiart (2024)
	*Daemonorops periacantha* Miq.^σ†Δ^	Medicinal food (Dusun)	Uwai lambat	Voeks and bin Nyawa (2006)
	*Korthalsia jala* J. Dransf. ^β†Δ^	Difficult labour (Dusun)	Rukatan	Martin et al. (2002)
	*Licuala bidentata* Becc.^β†Δ^	Magic rituals (Dusun)	Silad	Martin et al. (2002), Wiart (2024)
	*Licuala spinosa* Wurmb.	Difficult labour (Suluk)	Palma	Wiart (2024)
		Magic rituals (Dusun)	Silad	Wiart (2024)
	*Metroxylon sagu* Rottb.	Fatigue, medicinal food (Dusun)	Sagu	Martin et al. (2002), Noweg et al. (2003), Maid et al. (2017)
		Fatigue (Kadazan)	Rumbio	Martin et al. (2002)
	*Plectocomiopsis geminiflora* (Griff.) Becc.	Medicinal food (Dusun)	Baluak	Noweg (2003)
	*Oncosperma horridum* (Griff.) Scheff.^†Δ^	Medicinal food (Dusun)	Nibung	Martin et al. (2002)
	*Oncosperma tigillarium* (Jack) Ridl.	Medicinal food (Dusun)	Libung	Noweg et al. (2003)
	*Plectocomia mulleri* Bl.^σ†Δ^	Contraceptive (Dusun)	Mangkawaian	Martin et al. (2002)
	*Plectocomiopsis geminiflora* (Griff.) Becc.^†Δ^	Medicinal (Murut)	Ambarua	Kulip (2003a)
		Medicinal food (Dusun)	Temberuak	Maid et al. (2017)
	Salacca zalacca (Gaertn.) Voss	Body aches, gastritis (Bajau)		Wiart (2024)
**Commelinales Mirb. ex Bercht. & J. Presl (1820)**				
Commelinaceae Mirbel (1804)	*Amischotolype sphagnorrhiza* Cowley^β†Δ^	Hematemesis (Dusun)		Wiart (2024)
	*Commelina communis* L.	Bladder stones (Lundayeh)	Obat batu	Kulip et al. (2000)
	*Commelina nudiflora* L.	Fever (Dusun)	Soriau ngadau	Ahmad and Ismail (2003)
	*Forrestia griffithii* C.B. Clarke^σ†Δ^	Medicinal (Murut)	Tatapis da aputulan	Kulip (2003a)
Hanguanaceae Airy Shaw (1965)	*Hanguana malayana* (Jack.) Merr.^σ†Δ^	Fungal infection (Lundayeh)	Bunga	Kulip et al. (2000)
		Cramps, fatigue, poison antidote, sprains (Dusun)	Nalu kapar	Kulip (1997, 2003a)
		Cramps, fatigue, poison antidote, sprains (Kadazan)	Nalu kapar	Kulip (1997, 2003a)
		Gastritis (Murut)	Tatapis da umbir	Kulip (2003a)
	*Hanguana* sp.	Bleeding (Dusun)	Tambaka	Kulip (1997)
Pontederiaceae Kunth (1816)	*Eichhornia crassipes* (Mart.) Solms	Asthma, coughs, stomach aches, toothaches (Brunei)	Keladi agas	Wiart (2024)
	*Monochoria vaginalis* (Burm. f.) C. Presl	Medicinal food (Dusun)	Tayaan	Noweg et al. (2003)
Bromeliaceae Juss. (1789)	*Ananas comosus* (L.) Merr.	Dandruff, diabetes (Murut)	Tingkauran	Kulip (2003a), Nasir and On (2015)
		Bacterial skin infection, burns (Bonggi-Molbog)	Pisang	Lin (2022)
Cyperaceae Juss. (1789)	*Cyperus brevifolius* (Rottb.) Endl. ex Hassk.	Postpartum	Darah kimut merah	Lin (2022)
	*Cyperus rotundus* L.	Indigestion, irregular menstruation, skin diseases (Bisaya)	Rumput halia hitam	Mojiol et al. (2010)
	*Cyperus kyllingia* Endl.	Postpartum	Darah kimut	Lin (2022)
	*Cyperus* sp.	Jaundice (Dusun)	Wallang	Kulip (2014)
	*Hypolytrum nemorum* (Vahl) Spreng^†Δ^	Medicinal (Murut)	Balasan sungei	
Eriocaulaceae Martinov (1820)	*Eriocaulon longifolium* Nees ex Kunth^†Δ^	Canker sores (Dusun)	Kumpau sambangau	Voeks and bin Nyawa (2006)
Flagellariaceae Dumort. (1829)	*Flagellaria indica* L.	Paralysis, stroke (Murut)	Waau	Kulip (2003a)
		Coughs, flu, vomiting (Rungus)	Sogoto tumolong	Ahmad and Holdsworth (1995)
		Jaundice (Dusun)		Wiart et al. (2004)
		Jaundice (Kadazan)		Wiart (2004)
	*Scleria bancana* Miq.^†Δ^	Medicinal (Murut)	Onininsil	Kulip (2003a)
Poaceae Barnhart (1895)	*Bambusa vulgaris* Schrad. ex J.C. Wendl.	Poison antidote (Dusun)	Tamahang	Andersen et al. (2003), Kodoh et al. (2017)
		Poison antidote (Kadazan)	Tamahang	Andersen et al. (2003), Kodoh et al. (2017)
	*Bambusa* sp.1	Postpartum (Bajau)	Bulu	Wiart (2024)
	*Bambusa* sp.2	Sick children (Dusun)		Kodoh et al. (2017)
		Sick children (Kadazan)		Kodoh et al. (2017)
	*Bambusa* sp.3	Medicinal food (Dusun)	Buluh bukit	Noweg et al. (2003)
	*Coix lacryma-jobi* L.	Coughs, fever, flu, headaches, intestinal worms, medicinal food (Dusun)	Dalai	Kulip (2007a, 2009), Maid et al. (2017)
		Coughs, fever, flu, headaches, intestinal worms (Kadazan)	Dalai	Kulip (2007a, 2009)
	*Cymbopogon citratus* (DC.) Stapf.	Breathlessness, cold, flatulence, gastritis, itchiness, postpartum, vomiting (Bajau)	Serai mandi	Foo et al. (2016), Mahali et al. (2023)
		Flatulence (Brunei)	Serai makan	Foo et al. (2016)
		Fatigue, fever, flatulence (Dusun)	Sagumau	Ahmad and Ismail (2003)
		Coughs, fatigue, fever (Murut)	Sauhumau	Ahmad and Holdsworth (1994), Kulip (2003a, 2007a)
	*Dendrocalamus asper* (Schult. f.) Backer ex K. Heyne	Medicinal food (Dusun)	Buluh betong	Noweg et al. (2003)
	*Dinochloa scabrida* S. Dransf.^β†Δ^	Medicinal (Dusun)		Kulip (2003a)
	*Dinochloa scandens* (Blume) Kuntze^σ^	Medicinal (Murut)	Baran	Wiart (2024)
		Medicinal food (Dusun)	Wadan	Maid et al. (2017)
	*Dinochloa sublaevigata* S. Dransf.^β†Δ^	Flatulence, sore eyes, sore throats, Stomach aches (Dusun)	Bamboo badan	Kulip (2014)
		Bleeding (Dusun)		Kulip (2014)
		Bleeding (Kadazan)		Kulip (2014)
	*Dinochloa trichogona* S. Dransf.^β†Δ^	Sore eyes (Dusun)	Bulu badan	Voeks and bin Nyawa (2006)
	*Dinochloa* sp.1	Medicinal (Murut)	Baran	Wiart (2024)
	*Dinochloa* sp.2	Bleeding (Kadazan)		Wiart (2024)
	*Eleusine indica* (L.) Gaertn.	Asthma, bone pain, Diarrhea, food poisoning, flu, hemorrhoids, hair loss, postpartum,	Liagon (Murut)	Kulip (2003a)
		Wounds (Dusun)	Solinatad	Kulip et al. (2003a)
		Hair loss, wounds (Kadazan)	Solinatad	Kulip (2003a)
				Kulip (2003b), Kodoh et al. (2017)
		Hair loss (Rungus)		Kodoh et al. (2017)
		Postpartum (Bonggi-Molbog)	Bulig pingan	Lin (2022)
	*Garnotia acutigluma* (Steud.) Ohwi^†Δ^	Venereal diseases (Lundayeh)	Udu bulu	Kulip et al. (2000)
	*Gigantochloa levis* (Blanco) Merr. ^†Δ^	Blood in stools, Pancreatitis (Dusun)	Poring	Kulip et al. (2005), Kulip (2007a)
		Pancreatitis (Kadazan)	Poring	Kulip et al. (2005), Kulip (2007a)
	*Imperata cylindrica* (L.) Raeusch.	Hemoptysis, hematuria, nosebleeds (Bisaya)		Mojiol et al. (2010)
		Bacterial skin infection, chickenpox, fever, hepatitis, measles, rheumatism, kidney diseases, smallpox, thrush (Dusun)	Paka	Ahmad and Holdsworth (2003), Awang-Kanak (2021)
		Bacterial skin infection, chickenpox, fever, rheumatism (Kadazan)		Ahmad and Holdsworth (2003)
		Fever, smallpox, wounds (Murut)	Lalang	Ahmad and Holdsworth (1994), Kulip (2003a)
		Smallpox (Rungus)		Kodoh et al. (2014)
		Postpartum (Brunei)	Lalang	Kodoh et al. (2014)
		Stomach aches (Bajau)		Kodoh et al. (2014)
		Flatulence, fever (Sungai)		Kodoh et al. (2014)
	*Lophatherum gracile* Brongn.	Pancreatitis (Lundayeh)	Udu bulu	Kulip et al. (2000)
		Postpartum (Dusun)		Voeks and bin Nyawa (2006)
	*Miscanthus floridulus* (Labill.) Warb. ex K. Schum. & Lauterb^†Δ^	Feeling hot, flatulence (Dusun)	Bidau	Kulip (2014)
*Panicum palmifolium* J. Koenig^†Δ^	Malaria (Dusun)	tandaki		Maid et al. (2017), Wiart (2024)
		Malaria (Kadazan)	Tandaki	Wiart (2024)
	*Paspalum conjugatum* P.J. Bergius^†Δ^	Bone pain (Dusun)	Talinting	Kulip (2014)
	*Saccharum officinarum* L.	Flu (Dusun)	Tebu	Kodoh et al. (2017)
		Flu (Kadazan)	Tebu	Kodoh et al. (2017)
		Yellow fever (Rungus)		Kodoh et al. (2017)
	*Schizostachyum latifolium* Gamble^σ†Δ^	Blood in stools (Dusun)	Bulu gana	Voeks and bin Nyawa (2006)
	*Thysanolaena latifolia* (Roxb. ex Hornem.) Honda	Flu, headaches (Dusun)	Togiung	Kulip (2003b, 2014)
		Headaches, fever (Kadazan)	Togiung	Kulip (2014)
	*Zea mays* L.	Stomach aches (Murut)	Halai	Kulip (2003a)
	Incognita	Postpartum (Bonggi-Molbog)	Dalapas	Lin (2022)
**Zingiberales Griseb. (1854)**				
Cannaceae Juss. (1789)	*Canna indica* L.	Metrorrhagia (Brunei)	Bunga canna	Wiart (2024)
Costaceae Nakai (1941)	*Costus paradoxus* K. Schum.^β†Δ^	Nasal diseases (Dusun)	Badui	Voeks and bin Nyawa (2006)
	*Costus speciosus* (J. Koenig ex Retz.) Sm.	Coughs (Rungus)	Busu	Ahmad and Holdsworth (1995), Kulip (2003a)
		Asthma, bone pain, breathlessness fever, flu, headaches, postpartum (Dusun)	Sidbu sidbu	Kulip (2003b)
		Asthma, bone pain, fever, flu, headaches, respiratory diseases (Kadazan)	Sidbu sidbu	Ahmad et al. (2003), Kulip (2003b)
		Asthma, respiratory problems (Lundayeh)	Silok	Kulip et al. (2000)
		Asthma, chest pain, flu, headaches, respiratory problems, Stomach aches, swelling (Murut)	Linsasabu	Ahmad and Holdsworth (1994), Kulip (2003b)
		Fever, smallpox (Bajau)	Setawar halia	Wiart (2024)
	*Costus* sp.	Sprains (Dusun)	Subor subor	Kulip et al. (2005)
		Sprains (Kadazan)	Subor subor	Kulip et al. (2005)
Marantaceae R.Br. (1814)	*Donax canniformis* (G. Forst.) K. Schum.	Coughs, sore eyes (Dusun)	Lias	Kulip (2007a, 2014)
Musaceae Juss. (1789)	*Musa acuminata* Colla	Medicinal food (Dusun)	Pisang utan	Noweg et al. (2003)
	*Musa beccarii* N.W.Simmonds^β†Δ^	Medicinal food (Dusun)	Powok	Noweg et al. (2003)
	*Musa campestris* Becc.^β†Δ^	Medicinal food (Dusun)	Kelalang	Noweg (2003)
	*Musa hirta* Becc.^β†Δ^	Medicinal food (Dusun)	Tagutui	Noweg et al. (2003)
	*Musa paradisiaca* L.	Fever, skin cleaning, sore throats (Bugis)	Daung loka	Laudeh and Foo (2021), Wiart (2024)
	*Musa violascens* Ridl.^β†Δ^	Medicinal food (Dusun)	Pisang hutan	Noweg et al. (2003)
	*Musa* sp.1	Diarrhea (Dusun)	Togutui	Kulip et al. (2005)
		Diarrhea (Kadazan)	Togutui	Kulip et al. (2005)
	*Musa* sp.2	Postpartum (Bonggi-Molbog)		Lin (2022)
Zingiberaceae Martinov (1820)	*Alpinia galanga* (L.) Sw	Indigestion, skin diseases (Dusun)	Lengkuas	Kulip (2007b)
		Indigestion, skin diseases (Kadazan)	Lengkuas	Kulip (2007b)
	*Alpinia* sp.1	Join pains, rheumatism (Bajau)		Kodoh et al. (2017)
	*Alpinia* sp.2	Skin diseases (Dusun)	Tolidus	Kulip (2007b)
		Skin diseases (Kadazan)	Tolidus	Kulip (2007b)
	*Alpinia* sp.3	Smelly menstruation (Tidung)	Limpuyang	Kulip (2007b)
	*Alpinia* sp.4	Medicinal food (Dusun)	Sagang	Voeks and bin Nyawa (2006)
	*Boesenbergia pulchella* (Ridl.) Merr. ^β†Δ^	Skin diseases (Dusun)	Lipat	Kulip (2007b)
		Skin diseases (Kadazan)	Lipat	Kulip (2007b)
	*Boesenbergia rotunda* (L.) Mansf.	Hypertension, postpartum, sprains, stomach aches (Bajau)	Temu kuci	Wiart (2024)
		Hypertension, postpartum, sprains, stomach aches (Brunei)	Temu kuci	Wiart (2024)
	*Boesenbergia stenophylla* R.M Sm.^β^	Poisoning, stomach aches (Lundayeh)	Kaburo apad	Kulip (2007b)
	*Boesenbergia* sp.	Rheumatism (Dusun)	Layo tutumolong	Kulip (2007b)
	*Curcuma aeruginosa* Roxb.	Hypertension (Rungus)	Temu iring	Awang-Kanak (2022)
	*Curcuma caesia* Roxb.	Coughs (Bajau)	Kunyit hitam	
	*Curcuma longa* L.	Bone fracture, fever, flu, jaundice, skin diseases, flatulence, postpartum, wounds (Bajau)	Kunyit	Kodoh et al. (2017), Mahali et al. (2023)
		Insect stings, itchiness, jaundice, poison antidote, skin diseases, sprains, stomach aches (Dusun)	Kunyit	Ahmad and Holdsworth (2003), Kulip (2007b), Kodoh et al. (2017)
		Insect stings, itchiness, jaundice, poison antidote, skin diseases, sprains, stomach aches (Kadazan)	Kunyit	Ahmad and Holdsworth (2003), Kulip (2007b), Kodoh et al. (2017)
		Bacterial skin infection, bone fracture, flatulence, itchiness, jaundice, wounds postpartum, skin diseases (Brunei)	Kunyit biasa	Kodoh et al. (2017)
		Fungal infections (Murut)	Kunyit	Kulip (2003a)
		Coughs, fever, skin diseases, smallpox (Bugis)	Kunyit	Laudeh and Foo (2021)
		Bone fracture, flatulence,		Kodoh et al. (2017)
		jaundice, postpartum skin diseases (Illanun)		Kodoh et al. (2017)
		Bone fracture, jaundice flatulence, skin diseases (Sungai)		Kodoh et al. (2017)
		Fever, flu (Rungus)		Foo (2016)
		Postpartum (Bonggi-Molbog)	Kunit	Lin (2022)
	*Curcuma xanthorrhiza* Roxb.	Coughs, high cholesterol, postpartum, smallpox (Bajau)	Tambu kuning	Mahali et al. (2023)
		Toothaches (Sungai)	Kunyit hitam	Kulip (2007b)
	*Etlingera brevilabrum* (Valeton) R.M. Sm.^βπ^	Wounds (Dusun)	Sibu	Kulip (2014)
	*Etlingera coccinea* (Blume) S. Sakai & Nagam.^σπ^	Blood in stools, constipation, hypertension, medicinal food, unclean blood	Tuhau	Kulip (2007b), Maid et al. (2017)
	*Etlingera elatior* (Jack) R.M. Sm.^σ^	Fever, flatulence, medicinal food (Dusun)	Topu	Kulip (2007b), Noweg et al. (2003)
		Fever, flatulence (Kadazan)	Topu	Kulip (2007b)
	*Etlingera littoralis* (J. Koenig) Giseke	Fever, stomach aches (Bisaya)	Tepus	Mojiol et al. (2010)
	*Etlingera punicea* (Roxb.) R.M. Sm.^σ^	Beriberi, hypertension, medicinal food, unclean blood (Dusun)	Tuhau	Kulip (2003a, 2007b), Noweg et al. (2003)
	*Etlingera* sp.	Hemorrhoids, irregular menstruation (Dusun)	Teriwad	Kulip (2007b)
	*Globba francisci* Ridl.^β†Δ^	Feeling hot (Dusun)	Layo timbahan	Kulip (2007b)
	*Globba propinqua* Ridl.^β†Δ^	Menorrhagia (Dusun)	Mazolozo	Kulip (2007b)
	*Globba* sp.	Fatigue (Dusun)		Voeks and bin Nyawa (2006)
	*Hedychium longicornutum* Griff. ex Baker ^σ^	Fever (Bisaya)	Kunyit hantu	Mojiol et al. (2010)
	*Hedychium* sp.1	Bleeding, wounds (Dusun)	Sidbu	Kulip (2007b)
		Bleeding, wounds (Kadazan)	Sidbu	Kulip (2007b)
	*Hedychium* sp.2	Insect stings (Dusun)	Sidbu	Kulip (2007b)
		Insect stings (Kadazan)	Sidbu	Kulip (2007b)
	*Hornstedia* sp.1	Cold (Lundayeh)	Baku tabu	Kulip (2007b)
	*Hornstedia* sp.2	Medicinal food (Dusun)	Tolidus	Kulip (2007b), Foo et al. (2016)
		Medicinal food (Kadazan)	Tolidus	Kulip (2007b), Foo et al. (2016)
	*Hornstedia* sp.3	Medicinal food (Dusun)	Talirusan	Noweg et al. (2003)
	*Kaempferia galanga* L.	Coughs, cold, dysmenorrhea, fatigue, headaches, postpartum (Bajau)	Cekur	Foo et al. (2016)
		Fatigue, stomach aches (Dusun)		Kulip (2007b), Wiart (2024)
		Fatigue, stomach aches (Kadazan)		Kulip (2007b), Wiart (2024)
		Coughs, dandruff, food poisoning, sore throats (Brunei)	Cekur	Wiart (2024)
		Postpartum (Sungai)	Kusur	Kulip (2007b)
	*Plagiostachys albiflora* Ridl.^σ†Δ^	Medicinal food (Dusun)	Wongking	Kulip (2007b)
	*Plagisotachys* sp.	Medicinal food (Lundayeh)	Tumbu bachit	Kulip (2007b)
	*Tamijia* sp.1	Food poisoning (Paitan)	Kamlimigi sarou	Kulip (2007b)
	*Tamijia* sp.2	Stomach aches (Dusun)	Sisibu	Kulip (2007b)
		Stomach aches (Kadazan)	Sisibu	Kulip (2007b)
	*Zingiber officinale* Roscoe	Feeling hot, flatulence, postpartum, sprains, rheumatism (Dusun)	Hayo	Kulip (2007b)
		Feeling hot, flatulence, postpartum, sprains, rheumatism (Kadazan)	Hayo	Kulip (2007b)
		Flatulence (Murut)	Halia	Kulip (2003a), Kodoh et al. (2017)
		Rheumatism (Brunei)	Halia rajah	Kodoh et al. (2017)
		Cold (Bajau)	Halia	Foo et al. (2016)
	*Zingiber officinale* var. *rubrum* Thelaide	Blood circulation, fever, skin cleaning, swelling (Bajau)		Wiart (2024)
	*Zingiber purpureum* Roscoe	Fatigue, medicinal food (Rumanau)	Dangalai	Kulip (2007b)
	*Zingiber zerumbet* (L.) Roscoe ex Sm.	Hair loss (Bajau)	Lempoyang	Mahali et al. (2023)
		Medicinal food, fatigue (Tidung)	Limpuyang	Kulip (2007b)
		Wounds (Murut)	Benggalai	Wiart (2024)
	*Zingiber* sp.1	Toothaches (Dusun)	Tolidus	Kulip (2007b)
		Toothaches (Kadazan)	Tolidus	Kulip (2007b)
	*Zingiber* sp.2	Diarrhea, vomiting (Rumanau)	Dangalai taragang	Kulip (2007b)
	*Zingiber* sp.3	Fever, rashes (Bonggi-Molbog)	Binuak	Lin (2022)
**EUDICOTS**				
**Ranunculanae Takht. ex Reveal, (1992)**				
**Ranunculales Juss. ex Bercht. & J. Presl (1820)**				
Menispermaceae Juss. (1789)	*Coscinium fenestratum* (Gaertn.) Colebr.	Jaundice (Murut)	Babas lingungan	Kulip (2003a)
	*Fibraurea tinctoria* Lour.	Hypertension, malaria, wounds (Murut)	Tolungon	Kulip (2003b)
		Chest pain, fever, headaches, jaundice, malaria, stomach aches (Dusun)	Tapa buawang	Kulip (2003b) Voeks and bin Nyawa (2006)
		Chest pain, jaundice, fatigue, malaria, stomach aches (Kadazan)	Tapa buawang	Kulip (2003b, 2014)
		Eczema, malaria (Lundayeh)	Babas	Kulip et al. (2000)
	*Pycnarrhena tumefacta* Miers ^σπϕµ†Δ^	Bacterial skin infection (Lundayeh)	Fatagah	Kulip et al. (2000)
		Medicinal food (Murut)	Apa	Wiart (2024)
		Medicinal food (Dusun)	Apak	Noweg et al. (2003)
		Medicinal food (Kadazan)	Apak	Noweg et al. (2003)
	*Stephania corymbosa* Walp. ^σπϕ†Δ^	Poison antidote (Murut)	Penaki	Wiart (2024)
	*Tinospora crispa* (L.) Hook.f. & Thoms.	Diabetes, heart diseases, hypertension, intestinal worms, scabies, wounds (Bajau)	Urat nuali	Mahali et al. (2023)
		Hypertension, insect stings, malaria, sore eyes, stomach aches (Dusun)Hypertension, insect stings, malaria, sore eyes (Kadazan)	Sapai	Kulip (2007a)
		Sore eyes (Lundayeh)	Ubat it mato	Kulip et al. (2000)
		Malaria (Murut)		
	*Tinospora* sp.	Sore eyes, stomach aches (Dusun)	Wakau	Andersen et al. (2003)
		Sore eyes, stomach aches (Kadazan)	Wakau	Andersen et al. (2003)
**Proteanae Takht. (1967)**				
**Proteales Juss. ex Bercht. & J. Presl (1820)**				
Proteaceae Juss. (1789)	*Helicia serrata* Bl. ^σϕ†Δ^	Medicinal (Murut)	Andaun motuka	Wiart (2024)

β: Endemic solely in Borneo; σ: Sundaland; ϕ: Wallacea; π: Philippines; µ: Sahuland; †: no phytochemical and/or pharmacological study; Δ: worthy of further investigation.

### Core angiosperms

Medicinal core angiosperms are represented by 273 species and 58 families, mostly in the Fabids (150 species) and Malvids (102 species) (Table 5). In the Fabids, the largest medicinal families are the Euphorbiaceae (23 species), Fabaceae (35 species), and the Moraceae (14 species). Most medicinal plants in the core eudicots belong to the family Dilleniaceae (11 species) while in the rosids they all belong to the Vitaceae (nine species). Eighteen species (6.5%) of core angiosperms are endemic to Borneo including four in the Melastomataceae. A total of 89 species (33.2%) are not identified.

**Table 5.** Medicinal of Sabah (core angiosperms).

**CLADE Subclass Order** Family	Genus, species, authority	Symptoms/diseases (ethnic group)	Local name	References
**CORE EUDICOTS**				
**Dillenianae Takht. ex Doweld** **(2001)**				
**Dilleniales DC. ex Bercht. & J. Presl (1820)**				
Dilleniaceae Salisb. (1807)	*Dillenia excelsa* (Jack) Martelli^σπ^	Blood in stools, chest pain (Dusun)	Doingins	Kulip et al. (1997)
		Blood in stools, chest pain (Kadazan)	Doingins	Kulip et al. (1997)
		Body odor (Rungus)	Pampan kazu	Wiart (2024)
	*Dillenia grandifolia* Wall. ex Hook. f. & Thomson^σ†Δ^	Stomach aches (Murut)	Dudungin	Kulip (2003a)
	*Dillenia indica* L.	Mouthwash, toothaches (Rungus)	Morotud	Ahmad and Holdsworth (1995)
		Medicinal (Murut)		Wiart (2024)
	*Dillenia suffruticosa* (Griff. ex Hook. f. & Thomson) Martelli	Bleeding (Dusun)	Simpur	Wiart (2024)
	*Dillenia sumatrana* Miq.^σ†Δ^	Rheumatism (Dusun)		Kulip (2003a)
	*Dillenia* sp.	Bleeding (Rungus)	Rungin	Kulip (1997)
	*Tetracera akara* Merr.	Medicinal		Kodoh (2005)
	*Tetracera fagifolia* Bl.^σπϕ†Δ^	Diarrhea, sore eyes (Dusun)	Pampan mianai	Voeks and bin Nyawa (2006)
	*Tetracera indica* (Christm. & Panz.) Merr.	Appetite stimulant, cough, feeling hot, inflammation, headaches (Dusun)	Pampan	Yusoff et al. (2003)
		Coughs (Kadazan)		Yusoff et al. (2003)
	*Tetracera macrophylla* Hook.f.& Thomson^σ^	Diarrhea (Dusun)	Pampan indu	Voeks and bin Nyawa (2006)
	*Tetracera scandens* (L.) Merr.	Coughs (Dusun)	Tambar	Kulip (2014)
		Medicinal (Bonggi-Molbog)	Onitu	Lin (2022)
**Myrothamnanae Takht. (1997)**				
**Saxifragales Bercht. & J. Presl (1820)**				
Crassulaceae J.St.-Hil. (1805)	*Kalanchoe pinnata* (Lam.) Pers.	Headaches (Dusun)	Kapal	Yusoff et al. (2003)
		Headaches (Kadazan)	Kapal	Kulip (2003b)
		Wounds (Murut)	Tanom tombiog	Ahmad and Holdsworth (1995), Kulip (2003a)
		Feeling hot, fever, stomach aches (Brunei)	Setawar	Wiart (2024)
		Fever, stomach aches (Bajau)	Sedingin	Wiart (2024)
**ROSIDS**				
**Rosanae Takht. (1967)**				
Vitales Juss. ex Bercht. & J. Presl (1820)				
Vitaceae Juss. (1789)	*Ampelocissus imperialis* (Miq.) Planch^σ†Δ^	Hematemesis (Dusun)	Kamburat	Voeks and bin Nyawa 2006)
	*Ampelocissus ochracea* (Teijsm. & Binn.) Merr.^βπ†Δ^	Beriberi (Dusun)	Tabai	Wiart (2024)
	*Ampelocissus polita* (Miq.) Pelser^†Δ^	Medicinal food (Dusun)	Mban ambuk	Voeks and bin Nyawa (2006)
	*Ampelocissus winkleri* Lauterb.^β†Δ^	Bone pain, insecticide (Dusun)	Kamburat	Voeks and bin Nyawa (2006)
	*Leea indica* (Burm. f.) Merr.	Fatigue, headaches, sprains (Dusun)		
		Fatigue, headaches, sprains (Kadazan)		
		Sprains (Murut)		Kulip (2003a)
		Back pain, dysmenorrhoea, itchiness (Bajau)	Temali mali	Mahali et al. (2023)
	*Tetrastigma diepenhorstii* (Miq.) Latiff^σ†Δ^	Sprain (Murut)	Daramatin	Wiart (2024)
	*Tetrastigma* sp.1	Bacterial skin infection, dandruff, toothaches (Dusun)	Torumun dakon	Kulip (2014)
	*Tetrastigma* sp.2	Pancreatitis (Dusun)	Lipoi	Kulip (1997)
		Pancreatitis (Kadazan)	Lipoi	Kulip (1997)
	*Vitis trifolia* L.	Medicinal (Murut)	Susumoloi	Kulip (2003a)
**FABIDS**				
**Rosanae Takht. (1967)**				
**Cucurbitales Juss. ex Bercht. & J. Presl (1820)**				
Anisophylleaceae Ridl. (1922)	*Anisophyllea disticha* Baill.^σϕ^	Joint pain (Lundayeh)	Lapad tulang	Wiart (2024)
		Fatigue (Dusun)	Sapad	Voeks and bin Nyawa (2006)
Begoniaceae C. Agardh (1824)	*Begonia* sp.1	Fungal infection (Dusun)	Tonsom onsom	Kulip (2005)
		Fungal infection (Kadazan)	Tonsom onsom	Kulip (2005)
	*Begonia* sp.2	Vomiting (Dusun)	Sompotungu	Kulip (2005)
Cucurbitaceae Juss. (1789)	*Benincasa hispida* (Thunb.) Cogn.	Jaundice (Bajau)	Buah kundru	
		Fever (Dusun)		Kodoh et al. (2017)
		Fever (Kadazan)		Kodoh et al. (2017)
		Fever (Jawa)		Kodoh et al. (2017)
		Medicinal (Murut)	Kundru	Muhammed and Muthu (2015)
	*Cucumis sativus* L.	Gout (Bajau)	Timun	
	*Cucurbita pepo* L.	Flatulence, scalding (Dusun)	Tawadak	
		Scalding (Kadazan)		
	*Luffa cylindrica* M. Roem.	Hemorrhoids (Lundayeh)	Pucula	Kulip (2014)
	*Momordica charantia* L.	Coughs, diabetes, high cholesterol, hypertension, postpartum, yellow fever (Bajau)	Peria	Kodoh et al. (2014), Mahali et al. (2023)
		Hypertension (Dusun)	Popori	Kodoh et al. (2014)
		Hypertension (Kadazan)		Kodoh et al. (2014)
		Yellow fever (Rungus)		Kodoh et al. (2014)
		Hemorrhoids (Lundayeh)	Feria	Wiart (2024)
		Smallpox (Murut)	Poporia	Wiart (2024)
	*Sechium edule* (Jacq.) Sw.	Asthma (Bugis)	Labu	Laudeh and Foo (2021)
	*Trichosanthes cucumerina* L.	Swelling (Murut)	Molisun	Kulip (2003a)
Tetramelaceae Airy Shaw (1965)	*Octomeles sumatrana* Miq.^σπϕµ^	Bone pain, chest pain, fatigue, wounds (Dusun)	Binuang	Kulip (2003b), Kodoh et al. (2009)
		Bone pain, chest pain, fatigue, wounds (Kadazan)	Binuang	Kulip (2003b), Kodoh et al. (2009)
**Fabales Bromhead (1838)**				
Fabaceae Lindley (1836)	*Airyantha borneensis* (Oliv.) Brummitt^βπ†Δ^	Fatigue (Dusun)	Barayung	Voeks and bin Nyawa (2006)
		Fever, hypertension, toothaches (Murut)	Matamis	Wiart (2024)
	*Albizia saponaria* (Lour.) Blume^σπϕµ^	Medicinal	Langir	Kodoh et al. (2021)
	*Albizia* sp.	Fatigue (Dusun)	Sapang	Kulip (1997)
		Fatigue (Kadazan)	Sapang	Kulip (1997)
	*Archidendron clypearia* (Jack) I.C. Nielsen	Dandruff, itchiness, toothaches, thrush (Dusun)	Sogo	Voeks and bin Nyawa (2006), Wiart (2023)
		Dandruff, toothaches, thrush (Kadazan)		Wiart (2024)
	*Archidendron ellipticum* (Blume) I.C. Nielsen	Dandruff, poison antidote, pancreatitis (Dusun)	Sabano	Voeks and bin Nyawa (2006), Wiart (2023)
		Dandruff, poison antidote (Kadazan)		Wiart (2024)
	*Bauhinia kockiana* Korth.^σϕ^	Magic rituals (Murut)	Kulih bakah	Kulip (2003a)
	*Bauhinia* sp.1	Vertigo (Dusun)		Voeks and bin Nyawa (2006)
	*Bauhinia* sp.2	Bacterial skin infection (Dusun)	Kukuak	Kulip (2005)
	*Bauhinia* sp.3	Bacterial skin infection (Kadazan)	Turukon	Wiart (2024)
	*Caesalpinia bonduc* (L.) Roxb.	Chickenpox, malaria (Bajau)	Mentayang	Foo et al. (2016)
	*Caesalpinia sappan* L.	Anemia, body aches, chest pain, cold, postpartum, wounds (Bajau)	Sapang	Foo et al. (2016), Kodoh et al. (2021)
	*Cassia alata* L.	Asthma, beriberi, fatigue, jaundice, ringworms, scabies, skin diseases, stomach aches (Dusun)	Mangarut	Kulip (1997), Kulip et al. (2003a)
		Asthma, beriberi, jaundice, fatigue, ringworms scabies, skin diseases (Kadazan)	Kayabau	Kulip (1997), Kulip et al. (2003a)
		Skin diseases (Bugis)	Galenggang	Laudeh and Foo (2021)
		Stomach aches (Rungus)	Kurubau	Ahmad and Holdsworth (1995)
		Ringworm (Bonggi-Molbog)	Gompog	Lin (2022)
	*Cassia* sp.	Asthma (Dusun)	Wollu	Kulip (1997)
		Asthma (Kadazan)	Wollu	Kulip (1997)
	*Crotalaria pallida* Aiton	Flu (Dusun)	Ngrik ngrik	
	*Dalbergia parviflora* Roxb.	Fatigue, Jaundice, postpartum (Dusun)	Tampan kalabau	Kulip (1997)
		Fatigue, Jaundice, postpartum (Kadazan)	Tampan kalabau	Kulip (1997)
	*Dalbergia* sp.	Asthma, joint pain (Dusun)		
		Asthma, joint pain (Kadazan)		
	*Desmodium heterocarpum* DC.	Postpartum (Dusun)	Mampan sokot	Kulip (1997)
		Postpartum (Kadazan)	Mampan sokot	Kulip (1997)
	*Entada rheedei* Spreng.	Itchiness (Dusun)		Voeks and bin Nyawa (2006)
	*Erythrina* sp.	Medicinal (Dusun)	Radap	
	*Fordia splendidissima* (Miq.) Buisen subsp. *splenditissima*^σ^	Insecticide (Dusun)	Tawir	Voeks and bin Nyawa (2006)
	*Intsia palembanica* Miq.	Bleeding (Dusun)	Tupin	Kulip (1997)
		Bleeding (Kadazan)	Tupin	Kulip (1997)
	*Koompassia malaccensis* Maing^σ†Δ^	Allergy, asthma, bloating, body aches, convulsions, Blood in stools, gastritis, stomachaches, swollen gums, toothaches (Bajau)	Raja kayu	Foo et al. (2016)
	*Millettia nieuwenhuisii* J.J. Smith^β^**^†^**^Δ^	Thrush (Murut)	Ramus	Kulip (2003a)
	*Mimosa pudica* L.	Headaches, vertigo, wounds (Dusun)	Togop togop	Ahmad and Holdsworth (2003)
		Asthma, bacterial skin infection, insect bites, malaria, menorrhagia, skin diseases, stomach aches, swelling, wounds (Brunei)	Rumput semalu	Wiart (2024)
		Stomach aches (Murut)	Tenom molu	Kulip (2003a)
		Medicinal (Bonggi-Molbog)	Koya koya	Lin (2022)
	*Neptunia oleracea* Lour.	Medicinal food (Bajau)	Semalu air	Wiart (2024)
	*Parkia singularis* Miq.^σ†Δ^	Kidney stones (Murut)	Kundai	Kulip (2003a)
	*Parkia speciosa* Hassk.	Flu, hypertension, indigestion, kidney diseases	Petai	Wiart (2024)
		Medicinal food (Dusun)	Petai	Maid et al. (2017)
	*Sesbania grandiflora* L.	Headaches, fever, flu, smallpox Canker sores, coughs, Diarrhea, fever, headaches, phlegm, smallpox, stomach aches (Bajau)	Kemang tuli	Mahali et al. (2023); Wiart (2024)
	*Sindora* sp.	Medicinal (Murut)	Talikakasam	Kulip (2003a)
	*Spatholobus ferrugineus* (Zoll. & Moritzi) Benth**.^σ^**	Fatigue (Dusun)	Kalibid	Voeks and bin Nyawa (2006)
	*Spatholobus gyrocarpus* Benth.^σπ†Δ^	Thrush (Murut)	Ramus	Kulip (2003a)
	*Spatholobus* sp.1	Coughs, Diarrhea (Dusun)	Belohu	Kulip (2014)
	*Spatholobus sp*.2	Cold, fever (Dusun)	Lipoi	Kulip (2005)
		Cold, fever (Kadazan)	Lipoi	Kulip (2005)
	*Spatholobus* sp.3	Medicinal	Gingor	Wiart (2024)
	*Vigna unguiculata* (L.) Walp.	Fever, headaches (Dusun)	Balatong	Wiart (2024)
		Stomach aches (Bajau)	Kacang Panjang	
Polygalaceae Hoffmanns. & Link (1809)	*Polygala paniculata* L.	Coughs, fever, flatulence, gastritis, hypertension, toothaches (Dusun)	Gosok	Ahmad and Holdsworth (2003), Andersen et al. (2003), Kulip (2013)
		Hypertension, toothaches (Kadazan)	Mentimagas	Andersen et al. (2003)
	*Polygala* sp.	Heart diseases (Dusun)		Wiart (2024)
		Heart diseases (Kadazan)		Wiart (2024)
	*Xanthophyllum excelsum* (Blume) Miq.^†Δ^	Gastritis (Lundayeh)	Lapad atag	Kulip et al. (2000)
	*Xanthophyllum reticulatum* Chodat^β†Δ^	Magic rituals (Dusun)	Ngkruab	Wiart (2024)
**Fagales Engl. (1892)**				
Casuarinaceae R.Br. . (1814)	*Casuarina sumatrana* Jungh. ex de Vriese^σ^	Hair loss (Bajau)	Aru	Wiart (2024)
**Malpighiales Juss, ex Bercht. & J. Presl. (1820)**				
Achariaceae Harms (1897)	*Pangium edule* Reinw.^σπϕµ^	Medicinal food (Dusun)	Pangi	Maid et al. (2017)
	*Trichadenia philippinensis* Merr.^βπµϕ†Δ^	Magic rituals (Murut)	Ulok ulok	Kulip (2003a)
Clusiaceae Lindley (1836)	*Calophyllum* sp.	Back pain, bacterial skin infection kidney diseases, waist pain (Dusun)	Lawong	Wiart (2024)
		Back and waist pain, bacterial skin infection Kidney diseases (Kadazan)	Lawong	Wiart (2024)
	*Garcinia atroviridis* Griff. ex T. Anderson	Earache, postpartum (Dusun)	Asam gelugor	Maid et al. (2017)
	*Garcinia mangostana* L.	Hematemesis (Murut)	Timpurog	Kulip (2003a)
	*Garcinia parvifolia* (Miq.) Miq.^σϕ^	Medicinal food, postpartum (Dusun)	Kandis	Voeks and bin Nyawa (2006), Maid et al. (2017)
	*Hypericum japonicum* Thunb. ex Murr.	Ringworms, shingles (Dusun)	Tungkedem	Ahmad and Holdsworth (2003)
Dichapetalaceae Baill. (1886)	*Dichapetalum gelonioides* (Roxb.) Engl.	Cold, deafness, diabetes, dysmenorrhea, gastritis, hypertension, postpartum (Bajau)	Kokos	Foo et al. (2016)
		Fatigue (Dusun)		Voeks and bin Nyawa (2006)
	*Dichapetalum* sp.	Fatigue (Murut)	Akar urat	Wiart (2024)
Euphorbiaceae Juss. (1789)	*Codiaeum variegatum* (L.) Blume	Gastric ulcers (Kadazan)	Bunga mas	Ahmad and Holdsworth (2003)
	*Croton* sp.1	Sick children (Dusun)	Tolotok	Kulip (2005)
		Sick children (Kadazan)	Tolotok	Kulip (2005)
	*Croton* sp.2	Sick children (Dusun)	Kalayong	Kulip (1997)
		Sick children (Kadazan)	Kalayon	Kulip (1997)
	*Croton* sp.3	Fatigue (Rungus)	Rolok taragan	Wiart (2024)
	*Euphorbia hirta* L.	Bacterial skin infection, itchiness, swelling (Dusun)	Rumput susu	Ahmad and Ismail (2003)
		Bacterial skin infection, itchiness, swelling (Kadazan)	Kohonsizud	Ahmad and Ismail (2003)
		Bone fracture, sore eyes (Lundayeh)		Wiart (2024)
		Asthma, headaches (Brunei)	Rumput susu kambing	Wiart (2024)
		Fungal infection (Bonggi-Molbog)	Pitik pitik	Lin (2022)
	*Euphorbia prostrata* Aiton	Fever, hypertension, malaria (Dusun)	Nipon nipon betina	Wiart (2024)
		Yellow fever (Murut)	Galung galung	Wiart (2024)
	*Elateriospermum tapos* Bl.^σ^	Medicinal food (Dusun)	Pogo	Voeks and bin Nyawa (2006)
	*Homalanthus populneus* (Geiseler) Pax & Prantl^σπϕ^	Cramps (Dusun)	Dayang mato	Kulip (2013)
	*Homalanthus* sp.	Toothaches (Dusun)	Moropingan	Kulip et al. (2005)
	*Jatropha curcas* L.	Wounds (Dusun)	Jarak	Kulip (2013)
		Sprains (Bonggi-Molbog)	Tangan tangan	Lin (2022)
		Hemorrhoids (Bugis)	Tangan tangan	Laudeh and Foo (2021)
	*Jatropha podagrica* Hook.	Jaundice (Dusun)	Segima	Wiart (2024)
	*Macaranga gigantea* (Zoll.) Müll.Arg.	Thrush (Dusun)	Bangawong	Voeks and bin Nyawa (2006)
	*Macaranga gigantifolia* Merr.^βπ^	Diarrhea (Dusun)		Kulip (2003a)
		Diarrhea (Kadazan)		Kulip (2003a)
		Thrush (Murut)	Binawong	Kulip (2003a), Wiart (2024)
	*Macaranga tanarius* (L.) Müll. Arg.	Fatigue (Brunei)	Muyung	Wiart (2024)
	*Macaranga* sp.	Thrush (Dusun)	Limbukon	Kulip et al. (2005)
		Thrush (Kadazan)	Limbukon	Kulip et al. (2005)
	*Mallolus macrostachyus* (Miq.) Müll. Arg.^σ^	Bacterial skin infection, stomach aches, wounds (Dusun)	Jabai	Voeks and bin Nyawa (2006)
		Bacterial skin infection, stomach aches (Kadazan)	Dahu	Kulip (2014)
		Wounds (Rungus)	Dahu	Wiart (2024)
	*Mallotus miquelianus* (Scheff.) Boerl. ^σπ†Δ^	Cirrhosis (Lundayeh)		Wiart (2024)
	*Mallotus paniculatus* (Lam.) Müll. Arg.	Scabies, wounds (Dusun)	Dauk	Kulip (2013)
	*Manihot esculenta* Crantz	Child rashes, measles (Lundayeh)	Dikayu	Awang-Kanak et al. (2021)
		Diarrhea, fever, flatulence, headaches, wounds (Dusun)	Bayag kayu	
		Postpartum (Bugis)	Ubi	Laudeh and Foo (2021)
		Fever, headaches (Brunei)		Kodoh et al. (2017)
	*Pedilanthus tithymaloides* (L.) Poit.	Medicinal (Murut)	Tatapis tindukon	Kulip (2003a)
	*Ricinus communis* L.	Menorrhagia (Brunei)	Jarak jantan	
	*Sapium discolor* (Champ. ex Benth.) Müll. Arg.	Itchiness (Lundayeh)	Simbobolou	Kulip et al. (2000)
	*Sauropus androgynus* (L.) Merr.	Wounds (Dusun)	Totopus teropuk	Kulip (2014)
		Fever, sore eyes (Brunei)	Cangkuk manis	Wiart (2024)
		Medicinal (Murut)		Wiart (2024)
Flacourtiaceae Rich ex DC. (1824)	*Casearia grewiifolia* Vent.^σπϕπ^	Pancreatitis (Murut)	Salodkan	Wiart (2024)
	*Casearia rugulosa* Bl.^β†Δ^	Bacterial kin infections (Dusun)	Keh lupor	Voeks and bin Nyawa (2006)
	*Flacourtia rukam* Zoll. & Moritzi	Abdominal pain, headaches (Dusun)	Peripot	Kulip (1997)
		Abdominal pain, headaches (Kadazan)	Peripot	Kulip (1997)
	*Homalium foetidum* (Roxb.) Benth.	Medicinal (Murut)	Lulumada	Wiart (2024)
Linaceae DC. ex Perleb (1818)	*Indorouchera griffithiana* (Planch.) Hallier f.^σ†Δ^	Rheumatism (Dusun)	Kabul	Voeks and bin Nyawa (2006)
	*Philbornea magnifolia* (Stapf) Hallier f.^σπ†Δ^	Food poisoning (Dusun)		Voeks and bin Nyawa (2006)
Pandaceae Engl. & Gilg (1913)	*Galearia fulva* (Tul.) Miq.^†Δ^	Medicinal food (Dusun)	Sanggara	
Passifloraceae Juss. ex Roussel (1806)	*Adenia macrophylla* (Blume) Koord.^†Δ^	Seizure (Lundayeh)	War ruai	Kulip et al. (2000)
	*Passiflora foetida* L.	Hypertension, medicinal food (Dusun)	Lapak lapah	Npweg et al. (2003), Awang-Kanak et al. (2018), Awang-Kanak (2022)
		Hypertension, medicinal food (Bajau)	Lapak lapak	Awang-Kanak et al. (2018), Awang-Kanak (2022)
Peraceae Klotzsch (1859)	*Trigonopleura malayana* Hook.f.^σϕ†Δ^	Masticatory (Dusun)	Saripa	Wiart (2024)
	*Trigonostemon polyanthus* Merr.^βπ†Δ^	Poison antidote (Dusun)	Ambuk sagubang	Voeks and bin Nyawa (2006)
Phyllanthaceae Martinov (1820)	*Antidesma montanum* Bl.	Chest pain (Murut)	Damat mandalom	Kulip (2003a)
	*Baccaurea lanceolata* (Miq.) Müll. Arg.^σπ^	Abdominal pain, stomach aches (Murut)	Limposu	Kulip (2003a)
		Bone pain, wounds (Dusun)	Nipassu	Kulip (2003a)
		Bone pain, wounds (Kadazan)		
	*Bischofia javanica* Bl.	Blood circulation, diarrhea, stomach aches (Dusun)	Tugo	Andersen et al. (2003), Kulip
		Stomach aches (Kadazan)	Tongon	Andersen et al. (2003)
	*Breynia coronata* Hook.f.^σ^	Blood in stools, postpartum (Dusun)		Kulip (2003b)
		Blood in stools, postpartum (Kadazan)		Kulip (2003b)
	*Bridelia stipularis* (L.) Bl.	Fever, diabetes, postpartum, thrush (Dusun)	Belingkut	Kulip et al. (2003a)
		Fever, diabetes, postpartum, thrush (Kadazan)		Kulip et al. (2003a)
		Diabetes, thrush (Murut)	Bolingkut	Kulip et al. (2003a)
	*Glochidion macrostigma* Hook.f.^σ†Δ^	Cold (Murut)	Sondot laling	Kulip (2003a)
	*Glochidion rubrum* Bl.	Blood in stools (Dusun)	Dampul	Voeks and bin Nyawa (2006)
	*Glochidion* sp.	Rheumatism (Dusun)		Voeks and bin Nyawa (2006)
	*Phyllanthus niruri* L.	Fever (Bajau)	Dukung anak	
		Fatigue, hypertension (Dusun)		(Kulip 2013)
		Acne, ringworm (Bonggi-Molbog)	Buritas	Lin (2022)
	*Phyllanthus* sp.1	Diarrhea (Dusun)	Nipon nipon	Kulip et al. (2005)
	*Phyllanthus* sp.2	Foul body odor (Dusun)	Mongkolongkoi	Kulip (1997)
Putranjivaceae Meisn. (1842)	*Drypetes kikir* Airy Shaw^σ†Δ^	Medicinal food (Dusun)	Bubuk	Voeks and bin Nyawa (2006)
	*Drypetes macrostigma* J.J Sm.^β†Δ^	Medicinal food (Dusun)	Bubuk	Voeks and bin Nyawa (2006)
Rhizophoraceae Pers. (1806)	*Bruguiera parviflora* Wight	Fatigue (Rungus)	Langadoi	Wiart (2024)
	*Ceriops tagal* (Perr.) C. B. Rob	Fatigue (Bajau)	Tengal	
	*Rhizophora apiculata* Bl.	Constipation, halitosis, hypertension (Bajau)	Bangkita	Awang-Kanak et al. (2018), Wiart (2023)
Violaceae Batsch (1802)	*Rinorea* sp.	Coughs (Dusun)	Posiou	Kulip (1997)
		Coughs (Kadazan)	Posiou	Kulip (1997)
Oxalidales Bercht. & J. Presl (1820)				
Connaraceae R.Br. . (1818)	*Agelaea macrophylla* (Zoll.) Leenh^†Δ^	Fatigue (Murut)	Kalam malam	Wiart (2024)
	*Cnestis platantha* Griff. ^†Δ^	Coughs, fatigue, fever, flu, measles, sprains, stomach aches (Dusun)	Lingem	Wiart (2024)
		Fatigue, fever, flu, measles, sprains (Kadazan)		Wiart (2024)
	*Ellipanthus tomentosus* Kurz	Coughs (Dusun)	Bidon	
		Coughs (Kadazan)	Bidon	
	*Rourea mimosoides* Planch.	Blood in stools (Dusun)	Udang udang	Voeks and bin Nyawa (2006)
Oxalidaceae R. Br. (1818)	*Averrhoa bilimbi* L.	Hypertension (Bajau)	Belimbing pusung	Mahali et al. (2023)
		Fatigue (Dusun)	Tulod ulod	Wiart (2024)
		Fatigue (Kadazan)	Tulod ulod	Wiart (2024)
	*Averrhoa carambola* L.	Fever (Murut)	Belimbing	Muhamed et al. (2015)
Elaeocarpaceae Juss. ex DC. (1816)	*Elaeocarpus clementis* Merr.^β†Δ^	Medicinal (Rungus)	Timbarazung	Wiart (2024)
**Rosales Bercht. & Presl. (1820)**				
Moraceae Link (1831)	*Antiaris toxicaria* Lesch	Blowgun dart poison (Murut)	Paliu	Kulip (2003a)
	*Artocarpus elasticus* Reinw ex Bl.	Medicinal (Murut)	Puputul	Wiart (2024)
	*Artocarpus camansi* Blanco^σπϕµ^	Fever (Bajau)	Kemansi	Mahali et al. (2023)
	*Artocarpus heterophyllus* Lam.	Bacterial skin infections, sore throat (Dusun)	Nanko	Ahmad and Holdsworth (2003)
	*Artocarpus odoratissimus* Blanco^β^	Rough skin (Murut)	Tarap	Muhammed et al. (2015)
		Medicinal food (Dusun)	Terap	Maid et al. (2017)
	*Artocarpus tamaran* Becc.^β†Δ^	Medicinal (Dusun)	Timbagan	Wiart (2024)
	*Ficus deltoidea* Jack^σ^	Medicinal (Murut)	Agolauran	Kulip (2003a)
		Itchiness (Bugis)	Mas cotek	Laudeh and Foo (2021)
		Cold, sore eyes (Bajau)	Mas corek	Foo et al. (2016)
		Contraceptive (Dusun)		Wiart (2024)
		Contraceptive (Kadazan)		Wiart (2024)
	*Ficus elliptica* Hook ex Miq.^σ†Δ^	Shivers (Dusun)	Hintotobu	Kulip (1997)
		Shivers (Kadazan)	Hintotobu	Kulip (1997)
		Medicinal (Murut)	Silabon rindoh	Wiart (2024)
	*Ficus lepicarpa* Bl.	Fatigue, fever, ringworms (Dusun)	Tombuwasak	Wiart (2024)
	*Ficus racemosa* var *elongata* (King) M.F. Barrett^σ^	Medicinal (Murut)	Tandilan	Wiart (2024)
	*Ficus septica* Burm.f.^σπϕµ^	Flatulence, postpartum, wounds (Bajau)	Tisan	Mahali et al. (2023)
		Headaches, postpartum, stomach aches (Dusun)	Hintotobu	Maid et al. (2017), Awang-Kanak (2021)
		Postpartum (Kadazan)	Hintotobow	Kulip (1997)
	*Ficus* sp.1	Swelling (Dusun)	Tambunan	Kulip et al. (2005)
		Swelling (Kadazan)	Tambunan	Kulip et al. (2005)
	*Ficus* sp.2	Stomach aches (Dusun)	Togung	Kulip et al. (2005)
		Stomach aches (Kadazan)	Togung	Kulip et al. (2005)
	*Ficus* sp.3	Postpartum (Dusun)	Sintotobou kusai	Kulip et al. (2005)
		Postpartum (Kadazan)	Sintotobou kusai	Kulip et al. (2005)
	*Ficus* sp.4	Coughs, wounds (Dusun)	Tongkungkop	Kulip et al. (2005)
		Coughs, wounds (Kadazan)	Tongkungkop	Kulip et al. (2005)
Rhamnaceae Juss. (1789)	*Alphitonia incana* (Roxb.) Teijsm. & Binn. ex Kurz^βϕµ†Δ^	Headaches, itchiness, pancreatitis, jaundice, skin diseases, sprains (Dusun)	Bolotiom	Kulip (2003a)
		Headaches, itchiness, pancreatitis, jaundice, skin diseases, sprains (Kadazan)	Pukudita	Kulip (2003a)
	*Alphitonia* sp.	Itchiness (Dusun)	Pakodita	Kulip (2014)
	*Ziziphus borneensis* Merr.^β†Δ^	Hematemesis (Dusun**)**		Voeks and bin Nyawa (2006)
	*Ziziphus horsfieldii* Miq.^σπ†Δ^	Magic rituals (Rungus)		Wiart (2024)
Rosaceae Juss. (1789)	*Prunus arborea* (Bl.) Kalkman	Veterinary medicine (Rungus)	Kalanos	Wiart (2024)
	*Rubus moluccanus* L.	Sore eyes (Dusun)		Wiart (2024)
	*Rubus* sp.	Chest pain (Dusun)		Voeks and bin Nyawa (2006)
Urticaceae Juss. (1789)	*Dendrocnide elliptica* (Merr.) Chew ^βπ†Δ^	Medicinal (Rungus)	Ohopoi	Wiart (2024)
	*Leucosyke capitellata* Wedd.^σπϕµ†Δ^	Body aches, diabetes, hypertension, postpartum (Dusun)	Mandahasi	Ahmad and Ismail (2003), Wiart (2024)
		Earache, sore eyes (Kadazan)	Tahpoi	Andersen et al. (2003)
	*Pipturus arborescens* (Link) C.B. Rob.^βπϕµ^	Fever, flu (Dusun)	Bayug	Wiart (2024)
		Fever, flu (Kadazan)	Bayug	Wiart (2024)
	*Poikilospermum cordifolium* (Barg.-Petr.) Merr.^σ^	Malaise (Rungus)	Sodingkalan	Wiart (2024)
	*Poikilospermum suaveolens* (Bl.) Merr.	Medicinal food, postpartum (Murut)	Bunatol	Kulip (2003a)
		Sore eyes (Dusun)	Gunaton	Wiart (2024)
	*Poikilospermum* sp.1	Flatulence, postpartum, fatigue, wounds (Dusun)	Saringkalang	Wiart (2024)
	*Poikilospermum* sp.2	Medicinal food (Dusun)	Binatong	Noweg et al. (2003)
	*Pouzolzia* sp.	Difficult labor (Dusun)	Komburiong	Kulip et al. (2005)
		Difficult labor (Kadazan)	Komburiong	Kulip et al. (2005)
	*Urtica* sp.	Coughs (Dusun)	Mandahasi	Wiart (2024)
**MALVIDS**				
**Rosanae Takht. (1967)**				
**Brassicales Bromhead (1838)**				
Caricaceae Dumort. (1829)	*Carica papaya* L.	Diabetes, headaches, hypertension, kidney diseases, malaria, sinusitis (Bajau)	Kepayas	Awang-Kanak (2022), Kodoh et al. (2014)
		Contraceptive, constipation, gonorrhea, hemorrhoids, hypertension, lack of milk postpartum, weak uterus (Dusun)	Tepayas	Kodoh et al. (2014)
		Contraceptive, gonorrhea, hemorrhoids, lack of milk (Kadazan)		Kodoh et al. (2014)
		Malaria (Sungai)		Kodoh et al. (2014)
		Hypertension (Jawa)		Kodoh et al. (2014)
		Hypertension (Murut)	Betik	Kodoh et al. (2014)
		Hypertension (Rungus)		Kodoh et al. (2014)
		Diabetes, gout (Bugis)	Betik	Laudeh and Foo (2021)
		Fever (Brunei)	Betik	Kodoh et al. (2014)
		Fever, joint pain (Illanun)		Kodoh et al. (2014)
		Malaria (Suluk)		Kodoh et al. (2014)
		Hypertension (Bonggi-Molbog)	Kepayas rampayan	Lin (2022)
Cleomaceae Bercht. & J. Presl (1820)	*Cleome chelidonii* L.f.	Stomach aches (Murut)	Janggut kucing	Wiart (2024)
	*Gynandropsis gynandra* (L.) Briq	Hemorrhoids, rheumatism (Brunei)	Maman hantu	Mahmud and Razali (2016)
Moringaceae Martinov (1820)	*Moringa oleifera* Lam	Skin cleaning (Bugis)	Gemunggal	Laudeh and Foo (2021)
		Asthma, coughs, Diarrhea, Fever (Bajau)	Marrungai	Wiart (2024)
**Malvales Juss. ex Bercht. & J. Presl (1820)**				
Bixaceae Kunth (1822)	*Bixa orellana* L.	Gastritis, pain, white hair (Murut)	Suliabai	Muhammed and Muthu (2015)
Bombacaceae Kunth (1822)	*Bombax ceiba* L.	Fatigue (Dusun)	Kapok	Kulip (2003b)
		Fatigue (Kadazan)	Kapok	Kulip (2003b)
		Hematemesis (Murut)	kapok	Kulip (2003a)
	*Ceiba pentandra* (L.) Gaertn.	Fever, sprains (Dusun)		Kodoh et al. (2017)
		Sprains (Kadazan)		Kodoh et al. (2017)
		Bacterial skin infection (Bajau)		Kodoh et al. (2017)
	*Durio grandiflorus* (Mast.) Kosterm. & Soegeng^β†Δ^	Medicinal food (Dusun)	Durian mantua	Wiart (2024)
	*Durio graveolens* Becc.^σ†Δ^	Medicinal food (Murut)	Ruyan	Kulip (2003a)
	*Durio zibethinus* Rumph. ex Murray^σ^	Canker sores, stomach aches (Dusun)	Ratu	
Dipterocarpaceae Blume (1825)	*Parashorea malaanonan* Merr.^βπ^	Medicinal (Murut)	Melapi	Kulip (2003a)
	*Shorea macroptera* Dyer ^σ†Δ^	Food poisoning (Murut)	Omnompik	Kulip (2003a)
	*Shorea parvistipulata* F. Heim^β†Δ^	Fatigue (Murut)	Roloi	Kulip (2003a)
Malvaceae Juss. (1789)	*Abelmoschus esculentus* L.	Fever, flu (Bajau)	Kancang bendi	
	*Abutilon indicum* (L.) Sweet	Sprains (Dusun)	Tondorupang	Wiart (2024)
		Sprains (Kadazan)	Tondorupang	Wiart (2024)
	*Gossypium* sp.	Flatulence (Dusun)	Gapas	Kulip (2014)
	*Hibiscus rosa-sinensis* L.	Bacterial skin infection, coughs, dysmenorrhea, fever, hair loss, phlegm, mumps (Bajau)	Bungo raya	Foo et al. (2016); Kodoh et al. (2017), Awang-Kanak et al. (2021), Mahali et al. (2023)
		Asthma, bacterial skin infection, dysmenorrhoea, fever, jaundice, sprains, swelling, wounds (Dusun)	Tongkuango	Ahmad and Holdsworth (2003), Kodoh et al. (2017)
		Asthma, bacterial skin infection, dysmenorrhea, sprains, swollen fingers, wounds (Kadazan)	Tongkuango	Ahmad and Holdsworth (2003), Kodoh et al. (2017)
		Bacterial skin infection, fever (Murut)		Wiart (2024)
		Fever (Brunei)		Wiart (2024)
		Yellow fever (Rungus)		Kodoh et al. (2017)
		Headaches (Lundayeh)	Bunga raya	Kulip et al. (2000)
	*Hibiscus sabdariffa* L.	Cancer (Bajau)		Foo et al. (2016)
	*Hibiscus* sp.	Hypertension (Murut)		Kulip (2003a)
	*Sida acuta Burm*.f.	Stomach aches (Dusun)	Bulitotok	(Kulip 1997_
		Stomach aches (Kadazan)	Bulitotok	Kulip (1997)
	*Sida rhombifolia* L.	Poison antidote (Murut)	Dalupang	Kulip (2003a)
		Constipation, body aches (Bugis)	Canggadori	Laudeh and Foo (2021)
		Snakebites (Lundayeh)	Tahong	Kulip et al. (2000)
		Bacterial skin infection, Diarrhea, fever, headaches, skin diseases, swelling (Dusun)	Tatak tatak	Wiart (2024)
		Bacterial skin infection, diarrhea, fever, headaches, skin diseases, swelling (Kadazan)	Tatak tatak	Wiart (2024)
		Fever (Bugis)		Wiart (2024)
	*Urena lobata* L	Dysmenorrhoea, itchiness, back aches, dysmenorrhoea (Bajau)	Pulut pulut	Mahali et al. (2023)
		Constipation, thrush (Murut)	Injilokot	Kulip (2003a)
	*Urena* sp.	Bacterial skin infection (Dusun)	Tondorupang	Kulip et al. (2005)
		Bacterial skin infection (Kadazan)	Tondorupang	Kulip et al. (2005)
Tiliaceae Juss. (1789)	*Microcos antidesmifolia* (King) Burret ^σ†Δ^	Magic rituals (Rungus)	Kodong	Wiart (2024)
	*Microcos cinnamomifolia* Burret^β†Δ^	Magic rituals (Dusun)	Ngkodong	Wiart (2024)
Sterculiaceae Vent. (1807)	*Leptonychia heteroclita* Kurz^†Δ^	Stomach aches (Dusun)	Tembulang manok	Voeks and bin Nyawa (2006)
	*Scaphium macropodum* ^σ^	Medicinal (Dusun)	Kapayang	Wiart (2024)
	*Theobroma cacao* L.	Itchiness, skin diseases (Lundayeh)	Coco	Wiart (2024)
Thymeleaceae Juss. (1789)	*Phaleria papuana* Warb. ex K. Schum. & Lauterb.^βϕµ^	Asthma, allergy, diabetes, fatigue, heart diseases, hepatitis, hypertension, indigestion, kidney diseases, malaise (Bajau)	Mahkota dewa	Foo et al. (2016)
	*Wikstroemia androsaemifolia* Hand.-Mazz.^†Δ^	Headaches (Dusun)	Sinantali	Wiart (2024)
	*Wikstroemia ridleyi* Gamble ^σ†Δ^	Fatigue (Dusun)	Tindor	Wiart (2024)
	*Wikstroemia* sp.	Body aches (Dusun)	Tindot	Kulip (2005)
		Body aches (Kadazan)	Tindot	Kulip (2005)
**Myrtales Juss. ex Bercht. & J. Presl (1820)**				
Combretaceae R.Br. . (1810)	*Combretum nigrescens* King ^σ†Δ^	Internal injuries, wounds (Murut)	Damat dumalarom	Kulip (2003a)
	*Lumnitzera littorea* (Jack) Voigt	Bleeding (Bajau)	Santing	Mojiol et al. (2016)
Lythraceae J. St.-Hil. (1805)	*Lawsonia inermis* L.	Coughs (Dusun)	Inai	Wiart (2024)
		Itchiness, body aches (Murut)	Inai	Muhammed and Muthu (2015)
	*Punica granatum* L.	Dysmenorrhoea (Bajau)	Delima	Mahali et al. (2023)
Melastomataceae Juss. (1789)	*Clidemia hirta* (L.) D. Don	Stomach aches (Bisaya)	Senduduk paksa	Mojiol et al. (2010)
		Fever, flu (Dusun)		Wiart (2024)
	*Dissochaeta monticola* Bl.^σ†Δ^	Blowgun darts poison (Murut)	Bina	Wiart (2024)
	*Melastoma beccarianum* Cogn.^βΔ^	Blemishes (Dusun)	Duduk abai	Voeks and bin Nyawa (2006)
	*Melastoma malabathricum* L.	Diabetes, diarrhea, gout, gynecological disorders, hypertension, unclean blood (Bajau)	Lekat lekat	Mahali et al. (2023)
		Blood in stools, Diarrhea (Bisaya)	Senduduk	Mojiol et al. (2010)
		Blemishes, diarrhea, fever, measles, stomach aches, ulcers, postpartum, bleeding, wounds (Dusun)	Gosing gosing	Ahmad and Holdsworth (2003), Voeks and bin Nyawa (2006)
		Diarrhea, measles, stomach aches, bleeding, wounds (Kadazan)	Gosing gosing	Ahmad et al. (2003)
		Postpartum (Murut)	Lalarit	Wiart (2024)
		Diarrhea (Brunei)	Senduduk	
		Medicinal (Rungus)		(Ahmad and Holdsworth (1995)
	*Melastoma* sp.	Poison antidote, stomach aches, wounds (Dusun)		
		Poison antidote, stomach aches, wounds (Kadazan)		
	*Memecylon scolopacinum* Ridl.^β†Δ^	Hematemesis (Dusun)		Voeks and bin Nyawa (2006)
	*Pternandra gracilis* (Cogn.) N.P. Nayar^β†Δ^	Food poisoning (Dusun)	Banawar	Voeks and bin Nyawa (2006)
	*Pternandra* sp.	Diarrhea (Dusun)		Voeks and bin Nyawa (2006)
	*Sonerila crassiuscula* Stapf.^β†Δ^	Insect repellent (Lundayeh)	Bubuk kato	Wiart (2024)
Myrtaceae Juss. (1789)	*Decaspermum* sp.	Diarrhea, fever, flatulence (Dusun)		Wiart (2024)
	*Psidium guajava* L.	Blood in stools, diarrhea indigestion, postpartum, scalding, stomach aches (Bajau)	Liabas	Kodoh et al. (2014), Mahali et al. (2023)
		Diarrhea (Bugis)	Jambu	Laudeh and Foo (2021)
		Blood in stools, diarrhea, indigestion, stomach aches (Dusun)	Biabas	Yusoff et al. (2003), Ahmad and Holdsworth (2003), Kodoh et al. (2014)
		Blood in stools diarrhea, indigestion, stomach aches (Kadazan)	Liabas	Ahmad and Holdsworth (2003), Kodoh et al. (2014)
		Blood in stools, diarrhea (Lundayeh)	Giabas	Kulip et al. (2000)
		Indigestion, stomach aches (Rungus)		Kodoh et al. (2014)
		Indigestion, stomach aches (Sungai)		Kodoh et al. (2014)
		Indigestion, stomach aches (Jawa)		Kodoh et al. (2014)
		Indigestion, bacterial skin infection, stomach aches Indigestion, stomach aches (Illanun)	Biabas	Kodoh et al. (2014)
		Chickenpox, constipation, fever (Murut)	Kaliabas	Ahmad and Holdsworth (1994), Muhammed and Muthu (2015)
	*Rhodomyrtus tomentosa* (Aiton) Hassk.	Diarrhea (Dusun)	Jilong	Voeks and bin Nyawa (2006)
	*Syzygium aqueum* (Burm. f.) Alston ^σϕπµ^	Fever (Bajau)	Jambu madu	Foo et al. (2016)
	*Syzygium malaccense* (L.) Merr. & L.M. Perry	Diarrhea, stomach aches (Brunei)	Jambu air	Wiart (2024)
		Diarrhea, stomach aches (Bajau)	Jambu yagang	Ahmad and Ismail (2003)
Sonneratiaceae Engl. (1893)	*Sonneratia alba* Sm.	Cosmetic, Diarrhea, fever, medicinal food (Bajau)	Perepat	Mojiol et al. (2016)
**Sapindales Juss. ex Bercht. & J. Presl (1820)**				
Anacardiaceae R.Br. (1818)	*Lannea coromandelica* (Houtt.) Merr.	Wounds (Bugis)	Kayu Jawa	Laudeh and Foo (2021)
	*Mangifera caesia* Jack^σ^	Medicinal (Dusun)	Beluno	Maid et al. (2017)
	*Mangifera indica* L.	Medicinal food (Murut)	Longgom	Kulip (2003a)
	*Mangifera pajang* Kosterm.^β^	Cancer, high cholesterol, medicinal food (Dusun)	Bambangan	Maid et al. (2017)
	*Pegia sarmentosa* (Lecomte) Hand.-Mazz.^†Δ^	Wounds (Dusun)	Pegia	Kulip (2014)
	*Semecarpus cuneiformis* Blanco^σϕπ†Δ^	Wounds (Murut)	Kutang	Wiart (2024)
Burseraceae Kunth (1824)	*Canarium dichotomum* Miq.^σ^	Medicinal food (Dusun)	Kedondong	Maid et al. (2017)
	*Canarium littorale* Bl.^†Δ^	Medicinal food (Dusun)	Adal	Voeks and bin Nyawa (2006)
	*Canarium pilosum* A.W. Benn.	Medicinal food (Dusun)	Adal	Voeks and bin Nyawa (2006)
	*Dacryodes incurvata* (Engl.) H.J. Lam^σπ†Δ^	Medicinal food (Dusun)	Nguluon	Voeks and bin Nyawa (2006)
Meliaceae Juss. (1789)	*Lansium domesticum* Corrêa^σπϕ^	Toothaches (Dusun)	Langsat	Kodoh et al. (2017), Wiart (2024)
		Toothaches (Kadazan)	Langsat	Kodoh et al. (2017), Wiart (2024)
		Abdominal pain, diarrhea. stomach aches, Diarrhea (Murut)	Langsat	Kulip (2003a)
	*Xylocarpus granatum* J. Koenig	Blood in stools (Bajau)	Terbigit	Mojiol et al. (2016)
Rutaceae Juss. (1789)	*Citrus aurantium* L.	Menorrhagia, yellow fever (Bajau)		Kodoh et al. (2017)
	*Citrus hystrix* DC.	Bloating (Bajau)	Limau manuk	Mahali et al. (2023)
	*Citrus limon* (L.) Osbeck	Prevent sweating (Dusun)	Limau	Kulip (2013)
	*Citrus microcarpa* Bunge	Coughs, fever, smoking addiction, sore throats (Bajau)	Limau kasturi	
		Hypertension (Sungai)		Kodoh et al. (2017)
		Obesity (Jawa)		Kodoh et al. (2017)
	*Citrus* sp.	Sore throats (Dusun)	Kolopis	Wiart (2024)
	*Clausena excavata* Burm.f.	Venereal diseases (Lundayeh)	Alab layat	Kulip et al. (2000)
		Headaches, swelling, toothaches (Dusun)		Kulip et al. (2003a)
		Headaches, swelling, toothaches (Kadazan)		Kulip et al. (2003a)
		Toothaches (Rungus)	Untut paranok	Ahmad and Holdsworth (1995)
	*Luvunga motleyi* Oliver	Pancreatitis, smoking addiction (Dusun)	Tiga tiga	Wiart (2024)
	*Melicope* sp.	Beriberi (Dusun)	Pau	Kulip et al. (2005)
		Beriberi (Kadazan)	Pau	Kulip et al. (2005)
	*Micromelum minutum* Wight & Arn.	Medicinal (Murut)	Kimamansak	Kulip (2003a)
	*Micromelum* sp.	Flatulence (Dusun)	Paw	Kulip et al. (2005)
	*Murraya koenigii* (L.) Spreng.	Bruises, diabetes, indigestion, vomiting, swelling (Bajau)	Daun kari	Wiart (2024)
Sapindaceae Juss. (1789)	*Dimocarpus* sp.	Medicinal food (Dusun)	Mau gabuk	Voeks and bin Nyawa (2006)
	*Guioa bijuga* (Hiern) Radlk.^σπ†Δ^	Medicinal food (Rungus)	Anggil	Wiart (2024)
	*Guioa pleuropteris* (Blume) Radlk.^σπ†Δ^	Fatigue, flatulence, postpartum, stomach aches, thrush (Dusun)	Gulambir ayam	Kulip (2003b)
		Fatigue, flatulence, postpartum, stomach aches, thrush (Kadazan)		Kulip (2003b)
	*Lepisanthes amoena* (Hassk.) Leenh^σ^	Malaria (Dusun)	Kuinin	Wiart (2013)
	*Lepisanthes fruticosa* (Roxb.) Leenh.^σπϕ^	Fatigue (Dusun)	Banculuk	Voeks and bin Nyawa (2006)
		Medicinal food (Murut)	Talikasan	Kulip (2003a)
	*Lepisanthes* sp.1	Medicinal (Murut)	Bolilingasan	Wiart (2013)
	*Lepianthes* sp.2	Mumps (Dusun)	Boyongo	Kulip et al. (2005)
		Mumps (Kadazan)	Boyongo	Kulip et al. (2005)
	*Mischocarpus pentapetalus* (Roxb.) Radlk.^†Δ^	Medicinal (Rungus)	Tokingkid	Wiart (2024)
	*Nephelium macrophyllum* Radlk.^β†Δ^	Medicinal food, magic rituals (Dusun)	Mbokot	Voeks and bin Nyawa (2006)
	*Nephelium uncinatum* Radlk. ex Leenh.^σ†Δ^	Medicinal food (Dusun)	Kamanggis	Voeks and bin Nyawa (2006)
Simaroubaceae DC. (1811)	*Brucea javanica* (L.) Merr.	Dandruff, gastritis, lice, malaria intestinal worms, skin diseases, stomach aches (Dusun)	Garakat	Ahmad and Holdsworth (2003)
		Dandruff, gastritis, lice, malaria intestinal worms, skin diseases, stomach aches (Kadazan)	Monomopuru	Ahmad and Holdsworth (2003)
		Blood in stools (Rungus)		Kulip (2003b)
	*Eurycoma longifolia* Jack^σ^	Asthma, coughs, diarrhea, dysentery, fatigue, Voeks and Nyawa	Indigestion, malaria, stomach aches, tonic (Dusun)	Kulip (2003b, 2006), Maid et al. (2017)
		Asthma, fatigue, malaria, stomach aches (Kadazan)	Monompuru	Kulip (2003a, b), Kodoh et al. (2014)
		Fatigue (Bajau)	Tongkat Ali	Kodoh et al. (2014)
			Timuh	Kulip (2003b), Kodoh et al. (2014)
		Hypertension (Brunei)		Kodoh et al. (2014)
		Diabetes, hypertension (Illanun)		Kodoh et al. (2014)
		Medicinal (Murut)	Dulu	Kulip (2003a)
	*Eurycoma* sp.	Back pain (Dusun)	Mumud mondu	Wiart (2024)
		Back pain (Kadazan)	Mumud mondu	Wiart (2024)
**Caryophyllanae Takhtajan (1967)**				
**Caryophyllales Juss. ex Bercht. & J. Presl (1820)**				
Amaranthaceae Juss. (1789)	*Alternanthera sessilis* (L.) R.Br. ex DC.	Medicinal (Dusun)	Lalambi	Wiart (2024)
	*Amaranthus spinosus* L.	Blood in stools, difficulty urinating, flatulence, swelling, thrush, urinary tract infections (Dusun)	Samsam lodut	Ahmad and Ismail (2003), Wiart (2024)
		Blood in stools, difficulty urinating (Kadazan)		Ahmad and Ismail (2003)
		Seizure (Murut)	Sansam sau	Kulip (2003a)
	*Amaranthus* sp.	Medicinal food (Dusun)	Bayam liar	Noweg et al. (2003)
	*Celosia cristata* L.	Bacterial skin infection, coughs, hemorrhoids, swelling, wounds (Brunei)	Balung ayam	Wiart (2024)
	*Cyathula prostrata* (L.) Bl.	Insect bites (Murut)	Sansam bawi	Kulip (2003a)
		Abdominal pain, headaches (Dusun)		Wiart (2024)
		Abdominal pain, headaches (Kadazan)		Wiart (2024)
Basellaceae Raf. (1837)	*Anredera cordifolia* (Ten) Steenis	Gastritis (Murut)	Bina	Wiart (2024)
Nepenthaceae Dumort. (1829)	*Nepenthes ampullaria* Jack^σπϕµΔ^	Respiratory diseases (Lundayeh)	Telungau becuk	Kulip et al. (2000)
	*Nepenthes* sp.1	Syphilis (Dusun)	Kukuanga	Kulip et al. (2005)
		Syphilis (Kadazan)	Kukuanga	Kulip et al. (2005)
	*Nepenthes* sp.2	Diarrhea, fever (Bisaya)	Periuk pera	Mojiol et al. (2010)
Nyctaginaceae Juss. (1789)	*Bougainvillea* sp.	Bacterial skin infection, body aches, fever, flu, headaches, wounds (Bajau)	Bunga kertas	Foo et al. (2016)
Polygonaceae Juss. (1789)	*Polygonum minus* Huds	Cold, dysmenorrhea, indigestion (Bajau)	Kesum	Foo et al. (2016)
	*Polygonum odoratum* Lour.	Medicinal (Dusun)	Kesum	Wiart (2024)
	*Polygonum orientale* L.	Dandruff (Dusun)	Waying	Wiart (2024)
**Santalanae Thorne ex Reveal (1992)**				
Loranthaceae Juss. (1789)	*Scurrula* sp.	Toothaches (Dusun)	Tongom la’ an	Kulip et al. (2005)
		Toothaches (Kadazan)	Tongom la’ an	Kulip et al. (2005)
Olacaceae Jussieu ex R. Brown (1818)	*Scorodocarpus borneensis* (Baill.) Becc.^σ^	Medicinal food (Dusun)	Sembawang	Wiart (2024)

β: Borneo; σ: Sundaland; ϕ: Wallacea; π: Philippines; µ: Sahuland; †: no phytochemical and/or pharmacological study; Δ: worthy of further investigation.

### Upper angiosperms

Medicinal plants in this group encompass 165 species corresponding to 25 families. Most of these species belong to the lamiids (114 species or 69%) of which about half belong to the Rubiaceae. Thirteen species (7.8%) are endemic to Borneo, seven of which are in the Rubiaceae (Table 6).

**Table 6.** Medicinal of Sabah (upper angiosperms).

**CLADE Subclass Order** Family	Genus, species, authority	Symptoms/diseases (ethnic group)	Local name	References
**ASTERIDS**				
**Asteranae Takht. (1967)**				
**Ericales Bercht. & J. Presl (1820)**				
Actinidiaceae Engl. & Gilg. (1824)	*Saurauia fragrans* Hoogland^σ†Δ^	Ulcers (Dusun)	Longugan taragang	Wiart (2024)
	*Saurauia longistyla* Merr.^β†Δ^	Medicinal (Murut)	Usod usod	Kulip (2003a)
	*Saurauia* sp.1	Swelling (Dusun)	Longugan taragang	Kulip et al. (2005)
	*Saurauia* sp.2	Swelling (Dusun)	Longugan totomu	Kulip et al. (2005)
	*Saurauria* sp.3	Beriberi (Dusun)	Kebong	Kulip (2014)
Balsaminaceae Bercht. & J. Presl (1820)	*Impatiens balsamina* L.	Itchiness, snakebites, sprains (Lundayeh)		Wiart (2024)
Ebenaceae Gürke (1891)	*Diospyros andamanica* Bakh.^σ†Δ^	Poison for chicken (Murut)	Bokis manuk	Wiart (2024)
	*Diospyros elliptifolia* Merr.^σπ†Δ^	Medicinal (Rungus)	Radtak	Wiart (2024)
	*Diospyros foxworthyi* Bakh^σ^	Poison antidote, stomach aches	Sungkang seribu	Foo et al. (2016)
	*Diospyros wallichii* King & Gamble	Cachexia, jaundice (Lundayeh)	Lapad perurut	Kulip et al. (2000)
Lecythidaceae A. Rich. (1825)	*Barringtonia lanceolata* (Ridl.) Payens^β†Δ^	Insecticide, fish poisoning (Dusun)	Mpalang	Voeks and bin Nyawa (2006)
	*Barringtonia reticulata* (Blume) Miq.^σπϕ†Δ^	Insecticide, fish poisoning (Dusun)	Mpalang	Voeks and bin Nyawa (2006)
	*Barringtonia sarcostachys* (Merr.) Payens^β^	Insecticide, fish poisoning (Dusun)	Mpalang	Voeks and bin Nyawa (2006)
	*Barringtonia* sp.	Medicinal		Wiart (2024)
Primulaceae Batsch ex Borkh (1797)	*Ardisia* sp.1	Cold (Dusun)	Tolonsi	Wiart (2024)
	*Ardisia* sp.2	Fatigue, magic rituals (Dusun)	Rangup	Wiart (2024)
	*Embelia dasythyrsa* Miq.^σ†Δ^	Fever, medicinal food (Dusun)	Sowolikan	Wiart (2024)
	*Embelia philippinensis* A. DC^σ^	Medicinal food (Murut)	Papaling	Kulip (2003a)
	*Labisia pumila* (Bl.) Fern.-Vill	Postpartum (Bajau)	Kacip Fatimah	Wiart (2024)
	*Maesa* sp.1	Headaches, itchiness (Dusun)	Tonsom onsom	Kulip et al. (2005), Kulip (2014)
		Itchiness (Kadazan)	Tonsom onsom	Kulip et al. (2005)
Symplocaceae Desf. (1820)	*Symplocos odoratissima* Choisy ex Zoll.^σπϕ†Δ^	Fever, malaria (Lundayeh)	Lobo	Kulip et al. (2000)
**LAMIIDS**				
**Asteranae Takht. (1967)**				
**Gentianales Juss. ex Bercht. & J. Presl (1820)**				
Asclepiadaceae Borkh (1797)	*Asclepias curassavica* L.	Fever, bronchitis (Dusun)	Piak piak	Ahmad and Ismael (2003)
		Fever, bronchitis (Kadazan)	Piak piak	Ahmad and Ismael (2003)
	*Dischidia rafflesiana* Wall.^†Δ^	Cancer, skin diseases (Bajau)		Foo et al. (2016)
	*Hoya coronaria* Bl.	Body odor, pancreatitis (Dusun)	Lanau lanau	Wiart (2024)
		Body odor, pancreatitis (Kadazan)	Lanau lanau	Wiart (2024)
	*Hoya* sp.	Cancer (Dusun)	Bina	Andersen et al. (2003)
		Cancer (Kadazan)	Bina	Andersen et al. (2003)
		Medicinal (Murut)	Pongkukubab	Kulip (2003a)
Apocynaceae Juss. (1789)	*Allamanda cathartica* L.	Medicinal (Murut)	Bunga loceng	Muhammed and Muthu (2015)
	*Alstonia angustifolia* Wall.	Gastritis, wounds (Dusun)		Kulip (2003a)
		Gastritis, wounds (Kadazan)		Kulip (2003a)
	*Alstonia angustiloba* Miq.	Gastritis, malaria, sprains, wounds (Dusun)	Tombirong	Kulip (2003a)
		Gastritis, malaria, sprains, wounds (Kadazan)	Tembeilik	Salick et al. (1999), Kulip (2003a)
		Gastritis (Murut)		Kulip (2003a)
	*Alstonia macrophylla* Wall. ex G. Don	Seizure (Dusun)	Mangalang	
	*Alstonia scholaris (*L.) R.Br.	Diabetes, hypertension, malaria (Dusun)	Tombolik	Ahmad and Ismail (2003)
		Diabetes, hypertension, malaria (Kadazan)	Tombolik	Ahmad and Ismail (2003), Awang Kanak (2022)
	*Alstonia spatulata* Bl.	Wounds (Dusun)	Tembirog	Wiart (2024)
	*Catharanthus roseus* (L.) G. Don	Coughs, diabetes, lactation, malaria,	Kemunting Cina	Mahmud and Razali (2016)
		wounds (Brunei)		Awang-Kanak and Foo (2023)
	*Kopsia dasyrachis* Ridley^β^	Medicinal (Rungus)	Sarakad	Wiart (2024)
	*Kopsia* sp.^Δ^	Toothaches (Dusun)	Lodo lodo	Kulip et al. (2005)
		Toothaches (Kadazan)	Lodo lodo	Kulip et al. (2005)
	*Plumeria acuminata* W.T. Aiton	Constipation, haemorrhoids, internal pain (Murut)	Campaka	Wiart (2024)
	*Tabernaemontana macrocarpa* Jack	Bacterial skin infection, dermatitis (Dusun)	Lampada	Wiart (2024)
	*Tabernaemontana sphaerocarpa* Bl.	Medicinal (Dusun)	Lampad	Wiart (2024)
	*Tabernaemontana* sp.	Wounds (Dusun)	Lado lado	Kulip et al. (2005)
	*Willughbeia* sp.	Stomach aches	Combing	Wiart (2024)
Loganiaceae R. Br ex Mart (1827)	*Fagraea cuspidata* Bl.^βπ†Δ^	Chest pain, diabetes, fever, gastritis, jaundice, postpartum (Dusun)	Todopon	Kulip (2003b)
		Chest pain, diabetes, gastritis, jaundice (Kadazan)	Todopon	Kulip (2003b)
	*Fagraea racemosa* Jack	Chest pain (Dusun)	Todopon puok	Wiart (2024)
		Chest pain (Kadazan)	Todopon puok	Wiart (2024)
	*Strychnos ignatii* Berg.	Blow-gun dart poison (Murut)	Tataga do sangi	Kulip (2003a)
Rubiaceae Juss. (1789)	*Chassalia chartacea* Craib	Blurred vision (Dusun)	Lansi	Wiart (2024)
	*Gardenia tubifera* Wall.	Magic rituals (Rungus)	Piluzung	Wiart (2024)
	*Gardenia* sp.	Fatigue (Dusun)	Marabingo	Kulip (1997)
		Fatigue (Kadazan)	Marabingo	Kulip (1997)
	*Hedyotis auricularia* L.	Medicinal (Rungus)	Pipisoson tinanansad	Wiart (2024)
	*Hedyotis congesta* R.Br.	Wounds (Lundayeh)	Tapis apiris	Kulip et al. (2000)
	*Hedyotis rigida* (Blume) Walp	Wounds (Lundayeh)	Udu lomut	Kulip et al. (2000)
	*Hedyotis* sp.	Swelling (Dusun)	Mompu-ompu	Andersen et al. (2003)
		Swelling (Kadazan)	Monpu-ompu	Andersen et al. (2003)
	*Hydnophytum formicarum* Jack	Cancer, diabetes, hypertension (Lundayeh)	Sarang semut betina	Wiart (2024)
	*Ixora blumei* (Bl.) Zoll. & Moritzi^σ†Δ^	Hydrocele, swollen penis (Lundayeh)	Lapad bala	Kulip et al. (2000)
		Intestinal worms (Rungus)		Wiart (2024)
	*Ixora capillaris* Bremek^β†Δ^	Medicinal (Rungus)	Tagandap timulu	Wiart (2024)
	*Ixora fucosa* Bremek^β†Δ^	Appetite stimulant (Lundayeh)	Lapad lontong	Wiart (2024)
	*Ixora javanica* (Blume) DC.	Appetite stimulant (Lundayeh)	Busak wudan	Kulip et al. (2000)
	*Lasianthus inaequalis* Bl.^†Δ^	Fever (Lundayeh)	Pikolas	Mojiol et al. (2010)
	*Morinda borneensis* (Baill.) K. Schum^β†Δ^	Medicinal		Wiart (2024)
	*Morinda citrifolia* L.	Hypertension, joint pain (Bajau)	Mengkudu	Kodoh et al. (2009)
		Caries, gastric ulcers, hypertension, joint pain, dysmenorrhea, jaundice (Dusun)	Mengkudu	Kodoh et al. (2014)
		Caries, gastric ulcers, joint pain dysmenorrhea, hypertension, jaundice (Kadazan)	Bingkudu	Kodoh et al. (2014)
		Dysmenorrhea, hair loss, hypertension, white hair (Bugis)	Mengkudu	Laudeh and Foo (2021), Wiart (2024)
		Headaches, hypertension, poison antidote (Lundayeh)	Babas	Kulip et al. (2000)
		Cancer, caries, diabetes, dysmenorrhea, hypertension, joint pain, skin diseases (Brunei)		Mahmud and Razali (2016)
		Haemorrhoids, hypertension (Illanun)		
		Hypertension (Jawa)		
		Yellow fever (Rungus)		
		Headaches (Sungai)		
	*Mussaenda frondosa* L.	Headaches (Dusun)	Boliadok	Wiart (2024)
	*Mussaenda* sp.	Cold (Dusun)	Gayoh lubah	Kulip et al. (2005)
		Cold (Kadazan)	Gayoh lubah	Kulip et al. (2005)
	*Myrmecodia platytyrea* Becc.	Drunkenness, hypertension, poison antidote (Dusun)	Rajah ubat	Wiart (2024)
		Hypertension (Lundayeh)		Wiart (2024)
		Diabetes (Murut)	Sarang semut	Wiart (2024)
		Cancer, diabetes, fever, headaches, hypertension, kidney diseases, poison antidote, sinusitis, tuberculosis (Bajau)	Sarang semut	Wiart (2024)
	*Myrmecodia* sp.	Cancer		Wiart (2024)
	*Nauclea officinalis* (Pierre ex Pitard) Merr. & Chun.	Medicinal (Rungus)	Bongkol	Wiart (2024)
	*Nauclea orientalis* (L.) L.	Abdominal pain (Dusun)	Bongkol	Wiart (2024)
	*Neonauclea calycina* (Bartl. ex DC.) Merr.	Medicinal (Murut)^σπϕ^	Kembalu	Kulip (2003a)
	*Neonauclea gigantea* (Valeton) Merr.^β†Δ^	Diarrhoea, stomach aches, thrush (Dusun)	Mahitap	Wiart (2024)
	*Neonauclea* sp.	Diarrhoea (Dusun)	Intap	Kulip et al. (2005)
		Diarrhoea (Kadazan)	Intap	Kulip et al. (2005)
	*Neolamarckia* sp.	Beriberi (Dusun)	Towo	Kulip et al. (2005)
		Beriberi (Kadazan)	Towo	Kulip et al. (2005)
	*Oxyceros bispinosus* (Griff.) Tirveng.^†Δ^	Medicinal (Rungus)	Kovilan	Wiart (2024)
	*Paederia verticillata* Bl.^σπϕ†Δ^	Intestinal worms (Dusun)	Taud	Wiart (2024)
		Intestinal worms (Kadazan)	Taud	Wiart (2024)
	*Paederia* sp.1	Caries (Dusun)	Kombutong	Kulip et al. (2005)
		Caries (Kadazan)	Kombutong	Kulip et al. (2005)
	*Paederia* sp.2	Bleeding (Murut)	Ubat damat	Kulip (2003a)
	*Paederia* sp.3	Magic rituals (Rungus)	Papaid dazing	Wiart (2024)
	*Pavetta* sp.	Medicinal (Murut)	Buntungon	Kulip (2003a)
	*Praravinia suberosa* (Merr.) Bremek^β†Δ^	Medicinal (Murut)	Kingkimu	Kulip (2003a)
	*Psychotria gyrulosa* Stapf^β†Δ^	Headaches (Dusun)	Siroromuk	Wiart (2024)
	*Psychotria sarmentosa* Bl.	Itchiness (Murut)	Solovondo	Kulip (2003a)
	*Ridsdalea pseudoternifolia* (Valeton) J.T.Pereira^σ†Δ^	Medicinal (Rungus)	Sarakad rahat	Wiart (2024)
	*Uncaria acida* (W. Hunter) Roxb.	Caries (Dusun)	Langkawit	Wiart (2024)
	*Uncaria cordata* (Lour.) Merr.^†Δ^	Blood in stools, fatigue, fever, headaches, sprains (Dusun)		
	*Uncaria ferrea* (Bl.) DC	Medicinal (Rungus)	Ingangit	Wiart (2024)
	*Uncaria gambier* (W. Hunter) Roxb.	Flu, joint pain, wounds (Bajau)	Gambir	Foo et al. (2016)
	*Uncaria* sp.1	Cold, gout, headaches, Hematemesis (Dusun)	Kalawit	Kulip et al. (2005)
		Cold, gout, headaches, Hematemesis (Kadazan)	Kalawit	Kulip et al. (2005)
	*Uncaria* sp.2	Itchiness (Dusun)	kalait	Voeks and bin Nyawa (2006)
	*Urophyllum nigricans* Wernham^β†Δ^	Medicinal (Murut)		Wiart (2024)
**Lamiales Bromhead (1838)**				
Acanthaceae Juss. (1789)	*Acanthus* sp.	Earache (Dusun)	Tahipai	Andersen et al. (2003)
		Earache (Kadazan)	Tahipai	Andersen et al. (2003)
	*Andrographis paniculata* Nees	Hypertension, skin diseases (Bajau)	Hempedu bumi	Foo et al. (2016), Kodoh et al. (2017)
		Hypertension (Dusun)		Wiart (2024)
		Fever, hypertension, insect stings, snakebites, wounds (Brunei)		Kodoh et al. (2017)
		Hypertension (Murut)		Wiart (2024)
	*Clinacanthus nutans* (Burm. f.) Lindau	Cancer, diabetes, haemorrhoids, hypertension (Bajau)	Belalai gajah	Wiart (2024)
	*Graptophyllum pictum* (L.) Nees ex Griff.	Medicinal (Murut)	Lalamih	Kulip (2003a)
	*Hemigraphis* sp.	Chest pain, fatigue, headaches (Dusun)		Wiart (2024)
		Chest pain, fatigue, headaches (Kadazan)		Wiart (2024)
	*Hypoestes* sp.	Medicinal (Murut)	Matopait	Kulip (2003a)
	*Justicia gendarussa* Burm.f.	Back pain, bacterial skin infection, coughs, cramps, fatigue, flatulence, flu, headaches, joint pain, magic rituals, postpartum, rheumatism (Dusun)	Tambiau taragang	Wiart (2024)
		Back pain, bacterial skin infection, coughs, cramps, fatigue, flu, headaches, joint pain, postpartum, rheumatism (Kadazan)	Sikapapar	Wiart (2024)
		Stomach aches, magic rituals (Murut)	Solimbangan	Wiart (2024)
		Diarrhoea, headaches, rheumatism, swelling (Brunei)	Sarimbangun hitam	Ahmad and Holdsworth (2003)
Avicenniaceae Miq. (1845)	*Avicennia marina* (Forssk.) Vierh.	Medicinal food, stingray bites (Bajau)	Api api	Mojiol et al. (2016)
Bignoniaceae Juss. (1789)	*Oroxylum indicum* (L.) Vent.	Swelling (Murut)	Ulunan sangku	Kulip (2003a)
		Skin diseases, sprains, vomiting, wounds (Dusun)		Kulip (2003b)
		Skin diseases, sprains, vomiting, wounds (Kadazan)		Kulip (2003b)
Gesneriaceae Rich. & Juss. (1816)	*Cyrtandra areolata* (Stapf) B.L. Burtt^β†Δ^	Blood in stools, skin diseases (Murut)	Pohodo	Kulip (2003a)
	*Cyrtandra* sp.	Swelling (Dusun)	Lumpoh	Wiart (2024)
		Swelling (Kadazan)	Lumpoh	Wiart (2024)
	*Cyrtandromoea grandis* Ridl.^σΔ^	Medicinal (Murut)	Setawar	Wiart (2024)
Lamiaceae Martinov (1820)	*Coleus blumei* Benth.	Asthma, bacterial skin infection, coughs, fever, headaches, smallpox (Bajau)	Ati ati	Foo et al. (2016), Wiart (2024)
	*Gomphostemma* sp.	Bleeding (Murut)	Bunga susum	Kulip (1997)
	*Hyptis capitata* Jacq.	Stomach aches (Murut)	Baing baing	Kulip (2003a)
		Bacterial skin infection, cold, fever (Dusun)	Bala	Ahmad and Ismail (2003)
	*Ocimum basilicum* L.	Fever (Lundayeh)	Bawing	Wiart (2024)
		Medicinal food (Bajau)	Bawig	Awang Kanak et al. (2018)
		Medicinal food, convulsion (Bonggi-Molbog)	Sulasih	Lin (2022)
	*Ocimum tenuiflorum* Burm.f.	Coughs, gastric ulcer (Brunei)	Kemangi	Wiart (2024)
		Asthma, cold, coughs, fever headaches, indigestion (Bajau)		Foo et al. (2016)
	*Orthosiphon stamineus* Benth.	Diabetes, difficulty urinating, hypertension, kidney diseases (Dusun)		Awang Kanak (2022)
		Hypertension (Kadazan)		Awang Kanak (2022)
		Diabetes, hypertension (Murut)		Awang Kanak (2022)
		Hypertension (Sungai)		Awang Kanak (2022)
		Diabetes, hypertension (Lundayeh)		Awang Kanak (2022)
		Hypertension (Rungus)		Awang Kanak (2022)
		Diabetes, kidney diseases (Brunei)		Wiart (2024)
		Diabetes, hypertension, medicinal food (Bajau)		Awang Kanak et al. (2018)
	*Mentha arvensis* L.	Earache, medicinal food (Bajau)	Pudina	Foo et al. (2016)
Oleaceae Hoffmannsegg et Link (1809)	*Jasminum aculeatum* Blco^βπ†Δ^	Flatulence (Murut)	Onsom onsom	Kulip (2003a)
	*Jasminum bifarium* Wall^†Δ^	Sore eyes (Lundayeh)	Bunga melor	Wiart (2024)
Plantaginaceae Juss. (1789)	*Plantago major* L.	Coughs, dysmenorrhea, flu, hypertension, indigestion (Bajau)	Ekor anjing	Foo et al. (2016)
		Diabetes, urinary tract infections (Dusun)	Kulung	Ahmad and Holdsworth (2003)
		Urinary tract infections (Kadazan)	Kulung	Ahmad and Holdsworth (2003)
		Anaemia, cancer, diabetes, indigestion, wounds (Lundayeh)	Bunga	Kulip et al. (2000)
Scrophulariaceae Juss. (1789)	*Scoparia dulcis* L.	Fever, joint pain, sprains, stomach aches, vertigo (Dusun)	Telensi lensi	Wiart (2024)
		Fever, joint pain, malaise, stomach aches (Kadazan)	Kolimpang	Wiart (2024)
	*Scrophula* sp.	Asthma (Dusun)	Mata mata	Wiart (2024)
Verbenaceae Martinov (1820)	*Callicarpa longifolia* Lam.	Headaches, malaria, smallpox, shingles, Stomach aches (Dusun)	Sasad	Wiart (2024)
		Malaria, stomach aches (Kadazan)		Wiart (2024)
	*Callicarpa* sp.	Joint pain (Dusun)	Subol	Andersen et al. (2003)
		Joint pain (Kadazan)	Subol	Andersen et al. (2003)
	*Clerodendrum laevifolium* Bl.	Diarrhoea (Lundayeh)	Lilapo	Kulip et al. (2000)
	*Clerodendrum philippinum* Schauer	Bone fracture (Dusun)	Gutuk	Wiart (2024)
	*Clerodendrum* sp.	Medicinal food (Dusun)	Taum	Wiart (2024)
	*Lantana camara* L.	Itchiness (Dusun)	Ta ayam	Wiart (2024)
		Fever (Bonggi-Molbog)	Umbo pag’ asong	Lin (2022)
	*Hosea lobbii* (C.B. Clarke) Ridl. ^β†Δ^	Hematemesis (Dusun)	Tangalap	Wiart (2024)
	*Stachytarpheta jamaicensis* (L.) Vahl	Bleeding, diarrhoea, insect stings, medicinal food, poison antidote, sprains, skin diseases, snakebites (Dusun)	Soginap	Wiart (2024)
		Diarrhoea, poison antidote, sprains, skin diseases, snakebites, insect stings (Kadazan)	Tali tali	Wiart (2024)
		Bacterial skin infection, gonorrhoea swelling, wounds (Brunei)	Selasi dandi	Wiart (2024)
		Medicinal (Murut)	Indalupang	Kulip (2003a)
	*Vitex pubescens* (L.) Vahl	Postpartum (Brunei)	Kulimpapa	Wiart (2024)
		Beriberi, blood in stools, fever, hypertension, wounds (Dusun)	Bogong	Awang Kanak (2022), Wiart (2024)
		Beriberi, fever, hypertension, wounds (Kadazan)	Kuhim papo	Ahmad and Ismail (2003)
		Stomach aches, digestion, breathlessness (Bajau)	Kulimpapa	Wiart (2024)
		Medicinal (Rungus)		Ahmad and Holdsworth (1995)
**Solanales Juss. ex Bercht. & J. Presl (1820**)				
Convolvulaceae Juss. (1789)	*Ipomoea aquatica* Forssk.	Constipation (Lundayeh)	Kangkong	Wiart (2024)
	*Ipomoea batatas* (L.) Lam	Fever (Bajau)	Besina gerimit	Wiart (2024)
		Bacterial skin infection (Murut)	Ubi keledek	Wiart (2024)
	*Ipomoea* sp.	Medicinal food (Dusun)	Kasou	Noweg et al. (2003)
	*Merremia gracilis* E.J.F. Campb. & Argent^†Δ^	Asthma, diarrhoea, fatigue, jaundice, pancreatitis (Dusun)	Malagatas	Kulip (1997)
		Asthma, diarrhoea, fatigue, jaundice, pancreatitis (Kadazan)	Malagatas	Kulip (1997)
	*Merremia peltata* (L.) Merr.^†Δ^	Diarrhoea, flatulence, hair loss, Wiart stomach aches, wounds (Dusun)	Babas	Kulip (2003b, 2014, 2024)
		Diarrhoea, wounds (Kadazan)		Kulip (2003b)
Solanaceae Juss. (1789)	*Capsicum frutescens* L.	Difficult labor, fever, malaise, ringworms, sore eyes (Bajau)	Lodo poro	Mahali et al. (2023)
		Ailments associated with pregnancy, burns, itchiness, ringworms (Dusun)	Lado	Kulip (2014), Wiart (2024)
		Ailments associated with pregnancy, itchiness, ringworms (Kadazan)	Lado	Wiart (2024)
		Bacterial skin infection, canker sores, itchiness, rheumatism, wounds (Brunei)	Lada padi	Awang-Kanak and Foo (2023)
	*Nicotiana tabacum* L.	Ringworms (Lundayeh)	Sigup	Wiart (2024)
		Animal bites, insect stings, wounds (Dusun)	Sigup	Foo et al. (2016), Wiart (2024)
		Animal bites, insect stings, wounds (Kadazan)	Sigup	Foo et al. (2016), Wiart (2024)
		Wounds (Bajau)	Sigup	Foo et al. (2016)
	*Physalis minima* L.	Hypertension, intestinal worms, jaundice, high cholesterol (Bajau)	Letup letup	Mahali et al. (2023)
		Diabetes, hydrocele, hypertension, malaria (Dusun)	Tulapak	Awang-Kanak (2022)
		Diabetes, hypertension, malaria (Kadazan)	Tulapak	Wiart (2024)
		Hydrocele (Murut)	Pilanus	Wiart (2024)
		Sore throats (Brunei)		Wiart (2024)
		Medicinal (Rungus)		Ahmad and Holdsworth (1995)
	*Solanum erianthum* D. Don	Medicinal (Rungus)	Limbasong	Wiart (2024)
	*Solanum ferox* L.	Medicinal (Rungus)	Bintarung tondu	Wiart (2024)
		Medicinal food (Jawa)	Ricontom	Wiart (2024)
	*Solanum melongena* L.	Swollen gums, ulcers (Lundayeh)	Biterung	Wiart (2024)
		Medicinal food (Bajau)	Terung	Awang-Kanak et al. (2018)
	*Solanum nigrum* L.	Hypertension, flu, intestinal worms, medicinal food (Dusun)	Tutan hitam	Noweg et al. (2003), Wiart (2024)
		Hypertension, intestinal worms (Kadazan)	Tutan pura	Wiart (2024)
	*Solanum torvum* Sw.	Fever, medicinal food (Dusun)	Bintorung talum	Noweg (2003), Kulip (2013)
		Hypertension (Brunei)	Terung pipit	Wiart (2024)
		Medicinal (Murut)	Litahun	Ahmad and Holdsworth (1993), Kulip (2003a)
		Fever, medicinal food (Bonggi-Molbog)	Togung	Lin (2022)
		Medicinal food (Lundayeh)	Ulom	Kulip and Majawat (2000)
	*Solanum* sp.1	Dark urine (Dusun)	Tonsisiyah	Wiart (2024)
		Dark urine (Kadazan)	Tonsisiyah	Wiart (2024)
	*Solanum* sp.2	Swollen gums (Dusun)	Mansimang	Kulip et al. (2005)
		Swollen gums (Kadazan)	Mansimang	Kulip et al. (2005)
	*Solanum sp*.3	Medicinal food (Dusun)	Tutan puteh	Noweg et al. (2003)
**CAMPANULIIDS**				
**Asteranae Takht. (1967)**				
**Asterales Link (1829**)				
Asteraceae Martinov (1820)	*Ageratum conyzoides* L.	Wounds (Dusun)	Kambing kambing	Kulip et al. (2014)
		Gastritis, venereal diseases, wounds (Lundayeh)	Udu amek	Kulip et al. (2000)
	*Bidens pilosa* L.	Teething (Dusun)	Tondiokot	Ahmad and Holdsworth (2003)
	*Blumea balsamifera* DC.	Cold, gastritis, hypertension, postpartum (Bajau)	Sambun	Mahali et al. (2023)
		Abdominal pain, fatigue, fever, flatulence, insect stings, pancreatitis, postpartum, wounds (Dusun)	Tawawo	Kulip (1997), Ahmad and Holdsworth (2003), Kulip et al. (2014)
		Abdominal pain, fatigue, fever, insect stings, pancreatitis, postpartum, wounds (Kadazan)	Tawawo	Kulip (1997), Ahmad and Holdsworth (2003)
		Cold, constipation, flatulence, flu, gastritis, postpartum (Murut)	Tawawoh	Kulip (2003a)
		Fever, flatulence, postpartum (Lundayeh)	Ipong	Kulip et al. (2000)
		Intestinal worms (Rungus)		Kulip (2003a)
		Magic rituals, postpartum (Bonggi-Molbog)	Kelilibon	Lin (2022)
	*Blumea riparia* DC.	Hypertension (Murut)		Kulip (2003a)
	*Chromolaena odorata* (L.) R.M. King & H. Rob.	Wounds (Bonggi-Molbog)	Dolodoi	Pu Lin (2018)
	*Chromolaena* sp.	Veterinary (Dusun)	Nonokot	Wiart (2024)
	*Cosmos caudatus* Kunth	Medicinal food (Dusun)		Kulip et al. (2014)
		Osteoporosis, unclean blood (Brunei)		Wiart (2024)
		Ageing, blood circulation, diseases of the blood, fatigue, indigestion (Bajau)	Ulam rajah	Foo et al. (2016), Awang-Kanak and Foo (2023)
	*Crassocephalum crepidioides* (Benth.) S. Moore	Cancer (Murut)	Kinsau	Awang-Kanak and Foo (2023)
		Ageing, medicinal food (Dusun)	Koyundou	Awang-Kanak and Foo (2023)
		Ageing (Bajau)	Manggarang	Awang-Kanak and Foo (2023)
	*Crassocephalum* sp.1	Bone fracture (Dusun)	Lombon	Kulip et al. (2005)
		Bone fracture (Kadazan)	Lombon	Kulip et al. (2005)
	*Crassocephalum* sp.2	Bone fracture (Dusun)	Lokop	Kulip et al. (2005)
		Bone fracture (Kadazan)	Lokop	Kulip et al. (2005)
	*Elephantopus scaber* L.	Asthma, blood in stools, wounds	Salaman	
	*Elephantopus mollis* Kunth	Asthma, bacterial skin infection, bleeding, blood in stools, coughs, diarrhoea, flatulence, wounds (Dusun)	Salaman	Wiart (2024)
		Asthma, bleeding, blood in stools, flatulence, wounds (Kadazan)	Salaman	Wiart (2024)
		Blood in stools (Murut)	Honsigup	Kulip (2003a)
	*Erechtites hieraciifolius* (L.) Raf. ex DC.	Medicinal food (Dusun)	Bingol	Noweg et al. (2002)
	*Erechtites valerianaefolia* C.E.C. Fisch.	Medicinal (Murut)	Sumayon	Kulip (2003a)
	*Eupatorium odoratum* L.	Scalding, wounds (Dusun)	Rumput Malaysia	Wiart (2024)
		Scalding (Kadazan)	Rumput Malaysia	Wiart (2024)
		Scalding, wounds (Bajau)	Lunai lunai	Mahali et al. (2023)
		Scalding (Brunei)		
		Scalding, wounds (Murut)		
		Scalding, wounds (Rungus)		
		Scalding, insect stings (Sungai)		
		Scalding (Illanun)		
	*Gynura procumbens* (Lour.) Merr.	Fever, gastritis (Murut)	Sambung	Wiart (2024)
	*Synedrella nodiflora* (L.) Gaertn.	Fatigue (Murut)		Kulip (2003a)
**Apiales Nakai (1930)**				
Apiaceae Lindley (1836)	*Apium graveolens* L.	Gout (Bugis)	Daun sup	Laudeh and Foo (2021)
	*Centella asiatica* (L.) Urb.	Anemia, bacterial skin infection, blood circulation, cancer, diabetes, dementia, fever, jaundice, hypertension, indigestion, rashes, unclean blood, yellow fever (Bajau)	Pegaga	Awang-Kanak et al. (2018), Wiart (2024)
		Ageing, coughs, diarrhea, difficulty urinating, earache, hypertension, medicinal food, palpitations urinary tract infections, stomach aches (Dusun)	Salapid	Ahmad and Holdsworth (2003), Awang-Kanak (2022)
		Coughs, difficulty urinating, urinary tract infections, stomach aches (Kadazan)	Salapid	Ahmad and Holdsworth (2003)
		Canker sores, wounds (Brunei)		Wiart (2024)
		Yellow fever (Illanun)		Wiart (2024)
		Yellow fever (Sungai)		Wiart (2024)
		Fatigue (Lundayeh)	Pegago	Kulip and Majawat (2000)
		Sick children (Bonggi-Molbog)		Lin (2022)
	*Eryngium foetidum* L.	Medicinal food (Dusun)	Rumput tabug	Wiart (2024)
	*Eryngium* sp.	Sprains (Dusun)	Kosur	Kulip et al. (2005)
		Sprains (Kadazan)	Kosur	Kulip et al. (2005)
Araliaceae Juss. (1789)	*Aralia montana* Bl. ^σ† Δ^	Poison antidote (Dusun)	Golungang	Kulip (1997)
		Poison antidote (Kadazan)	Golungang	Kulip (1997)
	*Aralia* sp.1	Vertigo (Dusun)	Rupa	Kulip et al. (2005)
		Vertigo (Kadazan)	Rupa	Kulip et al. (2005)
	*Aralia* sp.2	Lack of milk (Dusun)	Rusap	Kulip et al. (2005)
		Lack of milk (Kadazan)	Rusap	Kulip et al. (2005)
	*Aralia* sp.3	Vertigo (Dusun)	Rusap	Kulip et al. (2005)
		Vertigo (Kadazan)	Rusap	Kulip et al. (2005)
	*Polyscias scutellaria* (Burm. f.) Fosberg	Fatigue (Lundayeh)	Polibas	Kulip et al. (2000)
	*Schefflera nervosa* (King) R. Vig.^σ†Δ^	Paralysis (Dusun)	Miang palat	Kulip (2014), Wiart (2024)
	*Schefflera petiolosa* (Miq.) Harm^β†Δ^	Asthma, bone pain, postpartum, skin diseases, wounds (Dusun)		Kulip (2003b)
		Asthma, bone pain, postpartum, skin diseases, wounds (Kadazan)		Kulip (2003b)
	*Schefflera* sp.	Pancreatitis (Dusun)	Malad palad	Kulip et al. (2005)
		Pancreatitis (Kadazan)	Malad palad	Kulip et al. (2005)
Pittosporaceae R. Br (1814)	*Pittosporum ferrugineum* W.T. Ait.	Bone pain (Dusun)	Saipang	Wiart (2024)

β: Borneo; σ: Sundaland; ϕ: Wallacea; π: Philippines; µ: Sahuland; †: no phytochemical and/or pharmacological study; Δ: worthy of further investigation.

## Main symptoms or illnesses

### General observation

According to the available data, most symptoms or illnesses are caused by infections, digestive problems, injuries, and pain (Tables 1–6). We also note that about half of these medicinal plants are used to treat one specific symptom or illness. There is also no therapeutic specificity at the subclass, order, and family level for peculiar symptoms or illnesses. That said, some patterns can be observed: lycophytes and inflammation, lamiids and cancer, Gleicheniaceae and sore eyes, Gnetaceae and fatigue, and Araceae and Zingiberaceae for inflammation. Plant families rich in tannins or phenolic compounds, such as Dilleniaceae, Phyllanthaceae, Melastomataceae, Myrtaceae, and Rubiaceae, are commonly used to treat bleeding, diarrhea, and blood in stools. It is also noteworthy that plants belonging to the genus *Macaranga* Thouars (1806) (Euphorbiaceae) are given for thrush (Table 5) while those in the genus *Saurauia* Willd. (1801) (Actinidiaceae) (Table 6) are commonly utilized for managing health issues related to inflammation. We also identified species from the genus *Ixora* L. (1753) (Rubiaceae) that are used as appetite stimulants (Table 6).

Ethnopharmacologists often face the challenge of classifying diseases associated with the symptoms being treated (Staub et al. 2015). For example, a plant traditionally used to relieve headaches may also display hypotensive properties, just as a plant intended for treating vision problems may have additional effects. Additionally, plant collections are often made by people without formal training in medicine, pharmacy, or pharmacology. Consequently, we organized the various symptoms and ailments in accordance with the Economic Botany Data Collection Standard (EBDC) developed by Cook (1995). Another notable point is that none of the available reports use ethnobotanical indices. This absence is beneficial because as Leonti (2022) wrote, “the cultural value and importance of plants in general, and more particularly medicinal plants and botanical medicines, cannot be summarized by numbers”. To further simplify and organize the available data, we can broadly categorize the frequency of utilization as follows: very frequent use (group I: 50 species or more), frequent use (group II: 25 to 49 species), moderate use (group III: 12 to 24 species), and rare use (group IV: 1 to 11 species).

### Infections and symptoms related to infections

Approximately one-third of the medicinal plants documented thus far are used to treat infections or symptoms associated with infections. Within this category, plants from group I are utilized for fever and viral infections. Most plants used for viral infections are given for cold and flu, and to a lesser extent for smallpox, yellow fever, chickenpox, measles, shingles, and mumps. Plants in group II treat bacterial skin infections and coughs. Plants in group III are employed to treat malaria, sore eyes, and fungal infections. Plants in group IV are given for ringworms, intestinal worms, sore throats, scabies, caries, thrush, urinary tract infections, venereal diseases, and gonorrhea. It is surprising that only one plant, *Myrmecodia platytyrea* Becc. (Rubiaceae), has been documented for the treatment of tuberculosis (Table 6), particularly given the devastating impact this disease had until the late 1950s (Koblenzer 1958).

There are ten species of plants fully identified and specifically used against fever and among these are *Equisetum ramosissimum* Desf. (Equisetaceae) (Table 2), *Uvaria cuneifolia* (Hook.f. & Thomson) L.L. Zhou (Annonaceae) (Table 4), *Artocarpus camansi* Blanco (Moraceae) (Table 5), and *Lasianthus inaequalis* Bl. (Rubiaceae) (Table 6).

Other observations revealed that four out of five lycophytes are used to treat fever, while plants belonging to the Menispermaceae, Simaroubaceae, and Apocynaceae are predominantly used to treat malaria. Some species are often employed for treating specific infections, such as *Cocos nucifera* L. (Arecaceae) with smallpox (Table 4).

### Digestive system disorders

Approximately one-third of the plants fall in this category, with species in group I used for stomach aches, diarrhea, and flatulence. We note that the plants from the family Zingiberaceae, which are used for stomachaches and flatulence, belong to group II (Table 4). There are five plant species with complete botanical names and specifically used for stomachaches such as *Dillenia grandifolia* Wall. ex-Hook. f. & Thomson. (Dilleniaceae) and *Leptonychia heteroclita* Kurz. (Sterculiaceae) (Table 5). Four plant species are specifically used for diarrhea of which *Tetracera macrophylla* Hook.f.& Thomson (Dilleniaceae) (Table 5) and *Neonauclea gigantea* (Valeton) Merr. (Rubiaceae) (Table 6). The high prevalence of digestive system ailments, particularly stomach pain and diarrhea, is likely to be associated with the consumption of unclean food and unsanitary water. Tanniferous and, therefore, astringent plants are commonly used for diarrhea. Examples include *Psidium guajava* L. (Myrtaceae) and *Melastoma malabathricum* L. (Melastomataceae) (Table 5) as well as plants within the genus *Neonauclea* Merr. (1915) (Rubiaceae) (Table 6). Some plants in group III are utilized as poison antidotes, and to treat gastritis, jaundice, pancreatitis, and blood in stools.

Other minor ailments in group IV include indigestion, constipation, abdominal pain, blood vomiting, hematemesis, canker sores, vomiting, and hemorrhoids. Other plants often employed for digestive ailments are *Cymbopogon citratus* (DC.) Stapf. (Poaceae) for flatulence (Table 4), *Clausena excavata* Burm.f. (Rubiaceae) for toothache, and *Elephantopus mollis* Kunth (Asteraceae) for blood in stools (Table 6). Plants in the genus *Alstonia* are frequently used to treat gastritis (Table 6).

### Injuries and related ailments

Approximately one-quarter of the medicinal plants identified in this review are used to treat injuries. Plants in group I are primarily employed for wounds, a practice likely influenced by traditional agricultural and forestry activities. It is important to note that before the arrival of British administrators in North Borneo, headhunting, and machete fights were common, contributing to the need for such remedies. Group II plants are used for sprains, bleeding, insect stings, as well as more rarely for scalding, snakebite, and for bone fractures. Minor uses include cramps, internal injuries, antidotes for blow-gun darts poison, nosebleeds, dislocation, burns, bruises, blisters, and stingray bites. Asteraceae are often used for bleeding and wounds, attributed to the presence of inulin, a dietary fiber (Zimmerman and Müller-Eberhard 1971). This is the case for *Eupatorium odoratum* L. (Table 6). Additionally, the Murutic and Dusunic ethnic groups are noted for using a greater variety of plants as antidotes for poisons compared to the other ethnic groups.

### Pain

Approximately one-quarter of the plants fall in this category, with species in group I used for headaches and in group II for toothaches. Plants in group IV for bone, joint and chest pain, as well as body aches. Minor uses include earache, waist pain, back pain, liver pain, and neuralgia.

### Inflammation and immune disorders

Fewer than one-quarter of the plants are used to address inflammation and immune disorders, primarily for conditions such as skin diseases, itchiness, asthma, and rheumatism. Other minor ailments treated in this category are ulcers, allergies, nasal diseases, child rashes, and eczema. Two lycophytes species out of five are used for asthma (Table 1). Plants in the genus *Alocasia* (Schott) G. Don (1839) (Araceae), are often used for itchiness (Table 4).

### Pregnancy, birth, puerperium disorders

Fewer than one-fifth of the plants are used for pregnancy, birth, and puerperium disorders. Most plants in this category are used for pregnancy, birth, or postpartum. Other minor uses include menorrhagia, metrorrhagia, dysmenorrhea, contraception, difficult labor, lack of milk, irregular menstruation, womb diseases, smelly menstruation, ailments associated with pregnancy, for a weak uterus, and pre-natal care. Plants in the genus *Lygodium* Sw. (1801) (Lygodiaceae) are used to treat womb-related diseases (Table 1), while *Blumea balsamifera* DC (Asteraceae) is commonly recommended for post-partum care. *Morinda citrifolia* L. (Rubiaceae) is employed to treat dysmenorrhea in four ethnic groups (Table 6). Plants with red flowers are often given for menstrual-related disorders (theory of signature). Examples include *Etlingera punicea* (Roxb.) R.M. Sm. (Zingiberaceae) and *Canna indica* L. (Cannaceae) (Table 4).

### Cardiovascular and circulatory system disorders

Fewer than one-fifth of these medicinal plants are used for cardiovascular and circulatory system disorders. Most plants in this category are used to treat hypertension. Other minor uses are blood circulation, heart diseases, breathlessness, anemia, swollen legs, unclean blood, and palpitations. One could examine the possible presence of hypotensive natural products (possibly indole alkaloids?) in *Chassalia chartacea* Craib (Rubiaceae) (Table 6).

### Metabolic and endocrine disorders

Fewer than one-fifth of these medicinal plants are given for metabolic and endocrine disorders. Murutic and Dusunic ethnic groups seem to employ fewer plants in this category probably because of their traditional way of life that incorporates the consumption of medicinal food combined with heavy physical work. Diabetes is the most common ailment followed by gout, high cholesterol, and obesity. Plants used for treating diabetes are found in the Piperaceae (Table 4) and the Phyllanthaceae, including *Bridelia stipularis* (L.) Bl. (Table 5) (Kulip et al. 2003a). This plant is utilized by the Dusuns, Kadazans, and Muruts. While the antidiabetic properties of *B. stipularis* have been validated *in vivo* (Khan et al. 2018), the active principle(s) remain unidentified.

### Genitourinary system disorders

Fewer than one-fifth of these medicinal plants are used for genitourinary system disorders. Difficulty urinating is the most prevalent ailment followed by kidney diseases, bladder stones, dark urine, kidney stones, hydrocele, and swollen penis.

### Neoplasms

Fewer than one-fifth of Sabah’s medicinal plants are used for cancer treatment. Interestingly, these plants come mainly from upper angiosperms (Table 6). An unidentified species in the genus *Myrmecodia* Jack. (1823) (Rubiaceae), is specifically used for cancer (Table 5) (Wiart 2024).

### Nervous system disorders

Less than a fifth of these medicinal plants are used for the treatment of nervous system disorders, mainly for seizures, paralysis, strokes, and dementia. None of the plants so far identified are used for psychiatric disorders.

### Unspecified health disorders

Approximately one-quarter of all recorded plants are used to treat symptoms or diseases that do not fit into any specific category, with most of these applications remaining unknown. Group I plants are used for fatigue. Other minor ailments are feeling hot, osteoporosis, ageing, chills, sick children, smoking addiction, malaise, vertigo, shivers, cachexia, and drunkenness. Gnetaceae such as *Gnetum gnemon* L. (Table 3) and plants in the family Annonaceae (Table 4) are often used for fatigue. *Eurycoma longifolia* Jack (Simaroubaceae) is used by three ethnic groups for fatigue (Table 5). *Physalis minima* L. (Solanaceae) is a Murut medicine for hydrocele (theory of signature?) (Table 5) (Wiart 2024).

### Other healthcare related uses

In this category, plants are primarily used as medicinal foods, in magical rituals, and for purposes such as insecticides, insect repellents, and lice control. Plant species in the families Zingiberaceae, Burseraceae, and Anacardiaceae are often used as medicinal foods (Table 5). Plants in the genus *Barringtonia* J.R. Forst. & G. Forst. (1776) (Lecythidaceae) are often used as an insecticide (Table 5). Plants in the genus *Goniothalamu*s (Blume) Hook. f. & Thomson (1855) (Annonaceae) employed for magic rituals (Table 4).

### Cosmetic uses

In this category plants are mainly employed for hair loss, dandruff, body odor, white hair, skin cleaning, hair wash, blemishes, rough skin, and to prevent sweating. Plants used for blemishes are common in the family Melastomataceae (Table 5).

### Miscellaneous uses

Plants in this category are used in veterinary medicine, as poisons for chickens and fish, and as blow dart poisons. Species in the genus *Barringtonia* are frequently employed for fish poisoning (Table 6).

## Medicinal uses according to ethnic groups

### General observations

Sabah does not have a unified traditional medical system, such as Ayurveda in India or Traditional Chinese Medicine. Instead, it encompasses a diverse range of botanical pharmacopeias practiced by its various ethnic groups. Most medicinal plants are utilized by ethnic groups of the Bornean linguistic group, primarily the Dusuns and Kadazans (Tables 1–6), followed by the Muruts. Additionally, plants are also used by the Bajaus, Lundayehs, and Bruneis, though to a lesser extent. Interestingly, the Muruts, Dusuns, and other communities from the Bornean linguistic group, who rely on shamans, exhibit a higher reliance on endemic plants in both medicinal and occult practices. This trend reflects their long-standing relationship with the natural environment. Notably, a significant number of plants, whose species names remain unidentified, are associated with the Murutic and, to a lesser extent, the Dusunic sub-groups. This observation highlights the substantial work yet to be undertaken in documenting and exploring their pharmacopeias. Another key finding is that ethnic groups that have converted to Islam appear less reliant on endemic plants, instead favoring well-documented species. This shift suggests a gradual loss of their core medicinal heritage. Furthermore, Muslim ethnic groups, such as the Bajaus, generally do not utilize plants for magical or occult rituals, indicating a divergence in cultural and medicinal practices.

**Figure 1.**
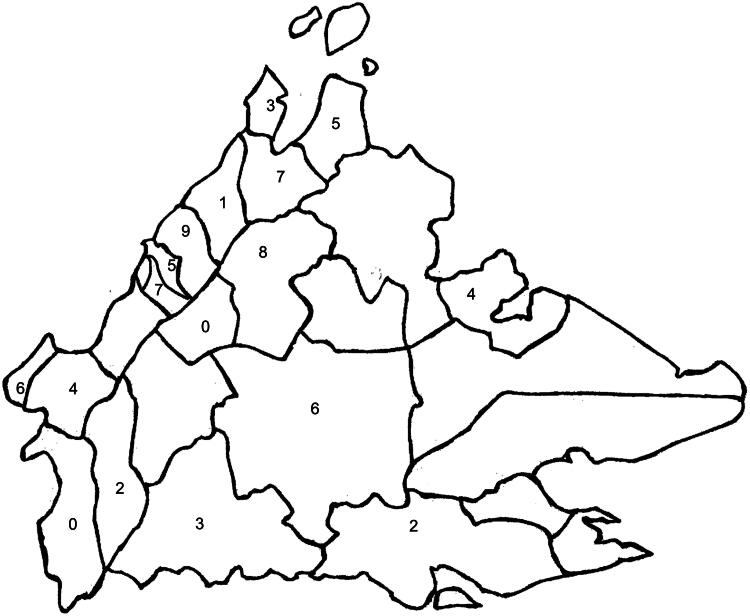
Schematic map of Sabah. 1**:** Putatan district; 2: Beluran; 3: Kinabatangan district; 4: Lahad Datu district; 5: Kunak district; 6: Semporna district; 7: Telupid district; 8: Keningau district; 9: Papar district; 10: Tambunan; 11: Kota Belud district; 12: Tenom district; 13: Kudat district; 14: Beaufort district; 15: Kota Kinabalu district; 16: Kuala Penyu district; 17: Penampang district; 18: Ranau district; 19: Tuaran district; 20: Sipitang district; 21: Kalabakan district; 22: Nabawan district; 23: Sandakan district; 24: Pitas district; 25: Tongod district; 26: Kota Marudu district

In examining the associations between medicinal plants and ethnic groups, Sabah’s medicinal plants can broadly be categorized into two main groups: (i) plants utilized by multiple ethnic groups and (ii) plants specific to a particular ethnic group. However, it is pertinent to note that data collectors often use broad umbrella terms rather than specifying the precise linguistic subgroup of the ethnic group studied. To enable and facilitate further studies on medicinal plants used specifically by certain ethnic groups, we provide a schematic map of Sabah in Figure 1.

A notable trend is the geographical and ecological distribution of endemic plant species used by different groups. For instance, many of the endemic plants utilized by the Dusuns are commonly found in mountain forests and on the slopes of Mount Kinabalu. This pattern is exemplified by selected Melastomataceae species. In contrast, plant species that form part of the Murutic pharmacopeia are predominantly sourced from primary rainforests, while medicinal plant species for the Bajaus are mostly found in mangroves and along beaches.

### Plants used by multiple ethnic groups

These medicinal plants are not endemic to Borneo; rather, they are widely distributed across the Asia-Pacific region, Africa, and Central America (Tables 2–6). In most cases, the active compounds in these plants have already been identified, and their pharmacological properties have been studied. We can cite for instance *Piper betle* L. (Piperaceae) and *Annona muricata* L. (Annonaceae) (Table 4).

We observe that ethnic groups within the Bornean linguistic family tend to use plants specific to their cultures, while non-Bornean groups, such as the Bajaus, often rely on plants shared with other ethnic groups and neighboring countries. Additionally, non-Bornean seafaring communities commonly use seashore plants, such as *Lumnitzera littorea* (Jack) Voigt (Combretaceae) and *Avicennia marina* (Forssk.) Vierh. (Avicenniaceae) (Table 5).

### Butungs

The Butungs live in Lahad Datu (Figure 1). They are Muslims who were once seafarers (King and King 1984). Currently, there is no available information regarding the medicinal plants they use.

### Jawa (Javanese stock)

During the early twentieth century, Javanese (Jawa) individuals were brought to North Borneo to work on plantations (De Silva 2009). Some Javanese communities are found near Sandakan (Figure 1) (King and King 1984). They use a few well-known food plants (Tables 4–6).

### Lundayehs (Kelabit family)

The Lundayehs reside in eastern Sabah, near the Sarawak border, where they settled approximately 100 years ago. They are also found in northern Sarawak, Brunei, and the northeastern corner of Kalimantan (King and King 1984). According to Shari Jeffri (personal communication) the word Lundayeh is made of “lun” which carries the meaning of a people, who is from the high country and “dayeh” meaning upstream rivers, they live in Murut areas and are best described as one of the hundreds of Dayak’s subtribes.

A total of 65 medicinal plants have been identified as being used by the Lundayeh ethnic group, of which 34 (52%) are specific to their community. Among these, four species are endemic (Tables 4–6). This highlights the unique pharmacopeia of the Lundayehs and underscores the importance of further research to document and preserve their traditional medicinal knowledge. The endemic *Ixora capillaris* Bremek (Rubiaceae) is used by both the Rungus and Lundayehs. Although demographically in the minority, they are, with the Dusuns and Kasazan, the largest users of medicinal ferns.

They use plants to treat of pathologies possibly related to excessive alcohol intake, such as cirrhosis and pancreatitis (Spicak et al. 2012), and appear to be the ethnic group that utilizes the most plants for venereal diseases, malaria, and itchiness. Being farmers and forest harvesters, they employ some well-known vegetables such as *Solanum melongena* L. (Solanaceae) or ornamental plants like *Impatiens balsamina* L. (Balsaminaceae) and even tobacco. The medicinal wealth of the Lundayehs raises the question of whether this linguistic group would not be better at home in the Bornean linguistic group.

### Bonggi-Molbogs (Palawanic family)

There is currently limited data available on the plants used by the Bonggi-Molbogs, an ethnic group found in Banggi Island, located at the northern tip of Sabah (Figure 1) (Kluge and Choi 2016; Lin 2021; Lin 2022). Eighteen plants were identified as being used medicinally by this ethnic group. None of these are endemic. They are the only ethnic group to utilize *Drynaria roosii* Nakaike (Polypodiaceae) (Table 1), a species of the genus *Musa* L. (1753) (Musaceae), a species of the genus *Zingiber* Mill. (1754) (Zingiberaceae) (Table 4), and *Chromolaena odorata* (L.) R.M. King & H. Rob. (Asteraceae) (Table 6). Although Muslims, they have female shamans, known as "boliyan," who use plants in magical rituals, particularly for postpartum care, such as *B. balsamifera*. Paul Lin (2021) recorded the use of flowering plants, although their identification has not yet been completed: “banag perempuan” and “banag laki” for postpartum as well as “binuak” for fever and rash in children, “bonid” and “tig lagbi” for fever, “dupdup bilaus” for pregnancy disconforts, “karam bunga” and “wa’ ag” to ward off evil spirits, “penulak” for itchiness, “roko” for infected eyes and for bloating, “tanggar masug” for vomiting, and “uru banta” as a detoxifier following illnesses.

### Illanuns (Danau family)

This ethnic group consists of pirates from Mindanao who settled on Sabah’s northern coast approximately 400 years ago (King and King 1984). They are seafarers and Muslims and have settled in Lahad Datu and Kota Belud (Figure 1). Ten plants have been identified as used medicinally by this group. These plants are well-known throughout Southeast Asia and are not specific to this ethnic group (Tables 4–6).

### Suluks (Tausug)

Suluks or Tausug are originally Muslim seafarers and slavers and from the Northeast coast of Mindanao who have settled in Telupid, Lahad Datu, Sandakan, Semporna, and Tawau (Figure 1) (King and King 1984; Pugh-Kitingan 2015). In the data available to us, they use two seashore plants found all over Southeast Asia (Tables 4–5). The medicinal flora of the Suluks living in Sabah remains unexplored perhaps for safety reasons. Historically, the Northern and Eastern part of Sabah has always been a dangerous zone, with for instance, a Suluk attack on the first and last British settlement on the Island of Balambangan in March 1775 (Julian 1963). Between 2014 and 2017, multiple cases of kidnapping and murder have been reported in Pulau Baik, Sandakan, and Semporna (Peters et al. 2019).

### Bugis

The Bugis reside around Sandakan, Tawau, and Lahad Datu (Figure 1) (King and King 1984). Currently, four plants have been identified as being medicinally used by the Bugis. They are the only ethnic group to use *Lannea coromandelica* (Houtt.) Merr. (Anacardiaceae) (Laudeh and Foo 2021) (Table 5).

### Ida’ans (Ida’anic group)

The Ida’ans were among the earlier inhabitants of Sabah’s southeastern coast and are known to be rice farmers and to harvest bird nests in caves (King and King 1984). Communities are found in Sandakan, Kinabatangan, and Lahad Datu. To date there is no data available on the medicinal plants used by this ethnic group.

### Bruneis (Malayic group)

The Bruneis (or Kedayans) are Muslims and live mainly along the coasts of Sipitang, Beaufort, and Kuala Penyu (Figure 1) (King and King 1984). Available reports document 43 plant species in their pharmacopeia, most of which are well-known in Southeast Asia and are also predominantly used by the Bajaus (Tables 1–6). Of these plants, 11 (25.5%) are unique to their ethnic group and often used for women’s health. We note that Muslim ethnic groups tend to utilize more plants for post-partum and childbirth related ailments than other groups.

### Sea Dayaks (Malayic group)

Some Sea Dayaks (or Ibans) migrated to Tawau searching for fertile land during the 1950s (Pugh-Kitingan 2015). Currently no data is available on the medicinal plants this ethnic group uses.

### Cocos Malays (Malayic group)

This ethnic group settled near Tawau in the 1950s from the Cocos Islands (Pugh-Kitingan 2015). Currently no data is available on the medicinal plants this ethnic group uses.

### Bajau (Baju group)

The Bajaus are found not only in Sabah but also in Eastern Indonesia and in the South Philippines—hence, they are not *sensu stricto* natives of Sabah (Evans 1953). It is estimated that they reached Sabah around 200 years ago from Johore in Peninsular Malaysia (Shari Jeffri, personal communication). They are traditionally coastal dwellers, with the West coast Bajaus in the Northwest, while the East coast Bajaus reside around Sandakan (Figure 1; King and King 1984). Of the 101 plants documented as being used medicinally by the Bajau, 41 (40%) are utilized exclusively by this group. They are often considered locally as “the guardians of the sea”, which likely explains why the plants in their pharmacopeia are gathered from the seashore, mangroves, and estuaries, such as *Caesalpinia bonduc* (L.) Roxb. (Fabaceae), *Neptunia oleracea* Lour. (Fabaceae), and *Rhizophora apiculata* Bl. (Rhizophoraceae).

In general, Bajaus use plants that are well-known in Southeast Asia, often available in wet markets locally known as “tamu” and most are non-endemic. As Muslims, the Bajaus typically do not use plants for magical rituals nor for treating symptoms related to alcoholism or poor hygiene. Instead, they primarily use plants for cosmetic purposes. We note that hypertension, gout, high cholesterol are not uncommon among them compared to other food-deprived ethnic groups. As fishermen, they have less need for plants for treating injuries, but they do utilize *A. marina* for stingray barb injuries (Table 5; Mojiol et al. 2016).

The medicinal use of plants among the Bajau is the broadest of the various groups in Sabah, suggesting that their botanical pharmacopeia has been well documented compared to other ethnic groups. Reports on the medicinal plants used by the Bajau primarily focus on the West Coast Bajau, leaving the pharmacopeia of the East Coast Bajau (Kagayans and Ubians) largely unexplored, likely due to safety concerns associated with traveling to Sabah’s East Coast.

### Bisayas (Bornean Dusunic sub-group)

Within the Dusunic sub-group, the Bisayas are Muslims, wet rice farmers, and reside on the southwest coast of Sabah near Brunei (King and King 1984; Lobel 2013). To date, seven plant species have been recorded as part of their medical practice. None of these plants are endemic and 4 are used exclusively by them such as *Etlingera littoralis* (J. Koenig) Giseke (Zingiberaceae) (Tables 4, 5). These plants are mainly used to treat fever and stomach aches.

### Dusuns (Bornean Dusunic sub-family)

According to Rutter (1929), the Dusun people refer to themselves as "tulun tindal" (land people), while the term Dusun may originate from a Bruneian word meaning "orchard", highlighting their connection to the interior and western regions of Sabah. The Dusun people have historically been the largest community followed by the Kadazans, Bajaus, and Muruts (Reid 1997).

A total of 445 species which account 62.4% of all the medicinal plants documented in Sabah are used by the Dusuns. This predominance could be explained by, at least in part, three factors: (i) demographically, they constitute the majority, (ii) they are among the first inhabitants of Sabah, and (iii) they mainly inhabit the northern, western and interior portions of Sabah which allows access to various flora types. A total of 191 plant species (39.7%) have been identified as being used exclusively by this ethnic group. Of these, about half of them are not yet fully identified and their medicinal uses are not yet specified, which indicates that the Dusun pharmacopeia is still incompletely known.

The Dusuns are the primary consumers of endemic medicinal plants, and most have not been phytochemically or pharmacologically studied. Compared to other ethnic groups, except for the Muruts and Kadazans, they tend to use more monocots, particularly Poaceae, and primary rainforest plants in their medicinal practices. The most common uses of these endemic plants include the treatment of hematemesis (vomiting of blood) and medicinal food. Compared to other ethnic groups, they seem to use plants linked to injuries (bleeding, sprains, cramps, bone fractures), lack of hygiene (skin diseases, dandruff, body odor), malnutrition (beriberi), and alcoholism (hematemesis, jaundice, pancreatitis). Other medical symptoms and conditions include fatigue (undoubtedly due to physical work and lack of nutrition), and bites from snakes and other insects. The use of plants for treating cancer, diabetes, and obesity is less common, as the Dusuns typically consume their own garden products. The Dusuns or “people of the orchard” are farmers and hunters explaining the use of plants as medicinal food, veterinary use, and fish poisoning.

Several plants, often endemic to Borneo, are used in magic rituals by the Dusuns. Ethnic groups of Bornean origin, like the Dusuns, traditionally believe that illnesses are caused by evil spirits or violations of primordial taboos (Voeks 2008). When afflicted with diseases, they consult elderly women shamans, known as "bobolian*"* or "bobolizan*"*, who communicate with "the other side" to explain the wrongdoing and provide guidance on which plants to use, the rituals to perform, when and where to harvest the plants (Evans 1953). An example is *Acorus calamus* L. (Acoraceae), or “komburongo*”*, believed to obstruct evil spirits and prevent them from doing harm during spiritual healings (Table 4) (On and Ishak 2016). Other important plants in this ethnic group’s occult practices include *Licuala spinosa* Wurmb. (Arecaceae) and *Licuala bidentata* Becc. (Arecaceae), locally known as "silad" (Table 4) (Martin et al. 2002; Wiart 2024). One might wonder what the value is in studying plants used for magic rituals. One answer is that some very useful drugs have been discovered from such plants such as morphine. Additionally, these magic rituals originated from a time when ethnic groups practiced animism. The plants used in these rituals have long-standing cultural and spiritual significance for these communities. Another example is *Pongamia pinnata* (L.) Pierre (Fabaceae) used for magic rituals for fever by the Dusuns (Pauline Yong Pau Lin personal communication) and extracts of this plant elicited antipyretic activity (Srinivasan et al. 2003). Access to modern medicine and religious conversion is bringing the rich Dusun, Kadazans, and Rungus shamanic culture to extinction (Reid 1997) while the younger generations are making “conscious efforts to avoid learning medicinal species” (Voeks and Nyawa 2001).

The Dusuns, compared to other ethnic groups, tend to consume monilophytes as medicinal foods. These need to be examined for their nutraceutical properties, particularly *Helminthostachys zeylanica* (L.) Hook. (Ophioglossaceae) (Noweg et al. 2003), which is also used for cancer by the Lundayehs (Table 2) (Kulip et al. 2000; Kulip 2007a). *Mangifera pajang* Kosterm. (Anacardiaceae), an endemic species, is traditionally used for managing cancer and high cholesterol (Maid et al. 2017) (Table 5). As such, it may hold potential as a nutraceutical.

### Kadazans (Bornean Dusunic sub-group)

Kadazans primarily inhabit the west coast, particularly in areas like Papar (Figure 1), whereas the Eastern Kadazans are in the southeastern part of Sabah. The term Kadazandusun emerged in the 1960s as a local political response to the rising influence of Muslim leadership from Peninsular Malaysia (Ueda 2009). Two hundred and three species of plants are currently used by the Kadazans. These plants and their therapeutic indications are, for the most part, like those used by the Dusuns. This ethnobotanical similarity may be attributed to the idea that the Kadazans are, in a sense, urban Dusuns, as suggested by the prefix *"kadai,"* which means store.

### Rungus (Bornean Dusunic sub-group)

Fifty-four plant species have been identified as being used for medicinal purposes by the Rungus. Among these, 23 species (or 43.5%) are specific to their pharmacopeia, including two endemic species: *Elaeocarpus clementis* Merr. (Elaeocarpaceae) and *Kopsia dasyrachis* Ridl. (Apocynaceae) (Table 5). The Rungus live mainly on the North coast of Sabah (King and King 1984), which explains why some of their medicinal plants are found in swamps, swamp forests, riverbanks, or mangroves such as *Bruguiera parviflora* Wight (Rhizophoraceae) (Table 5) and *E. clementis*. We note that the Rungus often use interesting plants from primary forests such as *Oxyceros bispinosus* (Griff.) Tirveng (Rubiaceae) and *K. dasyrachis* (Table 6). Additionally, the Rungus, Dusuns, and Kadazans are the only ethnic groups known to utilize plants in the genus *Kopsia* Blume (1823) (Apocynaceae), indicating that their botanical pharmacopeia is of particular interest. The Rungus pharmacopeia remains underexplored, with 18 plant species whose medicinal uses have not yet been defined. This could be because Sabah’s east coast is a dangerous region, making it difficult to conduct research. Additionally, we observed that the Rungus use four plants for the treatment of yellow fever. Some plants are also used in magical rituals, such as *Ziziphus horsfieldii* Miq. (Rhamnaceae) (Table 5).

### Plants used by other ethnic groups in the Bornean Dusunic sub-group

There are no data currently available on the plants used by the Dumpas, Kimaragangs, Kuijaus, Labuks, Lotuds, Papars, Sonsogons, Tatanas, Tobilungs, and others.

### Muruts (Bornean Murutic sub-group)

The Muruts, or "hill people," predominantly live in the southwestern parts of Sabah, near primary rainforests and rivers (Prentice 1969; Ahmad and Holdsworth 1994; Muhammed and Muthu 2015) as well as in the hilly uplands southern part of Sabah (Kulip 2003a). Although linguistically and culturally distinct from the Kadazandusun, they are sometimes grouped in political discourse under the umbrella term KDM (Kadazandusun-Murut) (Ueda 2009). They are wet rice farmers who established villages close to rivers and primary rainforests (Ahmad and Holdsworth 1994). Unlike ethnic groups outside of the Bornean linguistic group, they respect the plants with whom they live deeply in harmony. A total of 154 plants have been identified in their pharmacopeia, of which 73 species (or 47.4%) are exclusive to this ethnic group. These plants primarily grow in primary forests with geographical restrictions often limited to the Malay Peninsula, Indonesia, and the Philippines. Out of these, ten (6.4%) are endemic such as *Aristolochia papillifolia* Ding Hou (Aristolochiaceae) (Table 4).

By reviewing the plants used by the Muruts, we observe that they utilize all the Dipterocarpaceae. Additionally, they are the only group known to prepare blowgun poisons and antidotes for blowgun poisoning. A notorious case of fatal poisoning from a Murut blowgun dart occurred in the late nineteenth century, involving the German geologist Francis Xavier Witti (Wannier 2017). An example of a plant used in blowgun dart poisons is *Eusideroxylon zwageri* Teijsm. & Binn. (Lauraceae) (Table 4), a species found only in Borneo and Sumatra (Kulip 2003a). Another source of such poison is *Dissochaeta monticola* Bl. (Melastomataceae) (Wiart 2024) (Table 5). The active toxic constituents of these two plants are still unknown. *Antiaris toxicaria* Lesch (Moraceae) (Table 5) and *Strychnos ignatii* Berg. (Loganiaceae) (Table 6) are other source of such poison (Kulip 2003a). Therapeutically, substances in blow pipe poison have the potential to be developed as neuro-muscular medicines.

Like the Dusuns, the Muruts resort to shamans but call them “lumahon” (Fung 2004). They use the endemic plants *Goniothalamus woodii* Merr (Annonaceae) and *Ziziphus borneensis* Merr. (Rhamnaceae) for magic rituals (Wiart 2024). It should also be noted that a significant portion of the plants used by this ethnic group have medicinal uses that remain undefined, indicating that the Murut botanical pharmacopeia is still largely unexplored.

### Plants used by other Bornean Murutic sub-groups

There is currently no data available on the plants used by the Bookans, Ganas, Kalabakans, Keningaus, Paluans, Sembakungs, Serundungs, Tagals, Timugons, and others. Note that Murutic sub-groups are also found in North Kalimantan (Lobel 2013).

### Sungai (Bornean Paitanic sub-group)

The Sungai people are Muslims residing principally along the East coast rivers (the word “Sungai” means river in Malay). A total of seven plant species have been identified as being used by them, are well-known in Southeast Asia, and none are specific to their group, possibly due to the loss of core cultural knowledge (Tables 4–6). It is unclear whether these studies specifically address the Abai Sungai or Sungai *sensu lato*.

### Plants used by other ethnic groups in the Bornean Paitanic sub-group

Reports on other Paitanic ethnic groups are currently very limited. Two gingers are recorded as used by the Rumanaus: *Zingiber purpureum* Roscoe (Zingiberaceae) and a plant in the genus *Zingiber* (Kulip 2007b) (Table 4). *Tamijia* sp.1 (Zingiberaceae) is the only plant known to be used by the Paitans (Kulip 2007b). No data is available on medicinal plants used by the Kalabuans, Lingkabaus, Lobus, Sinabus, Subpans, and Upper Kinabatangans. Speakers of Paitanic languages are primarily distributed along the rivers of eastern Sabah and studying their pharmacopeia could bring to light a number of riparian species.

### Tidungs (Bornean tidung sub-group)

They live on the Southeast coast of Sabah and come from Indonesia (King and King 1984). So far, only one plant has been identified as being used by the Tidung: *Zingiber zerumbet* (L.) Roscoe ex Sm. (Zingiberaceae) (Table 4) (Kulip 2007b).

## Plants of phytochemical and pharmacological interest

### General observations

Most of the endemic plants that have not been subjected to phytochemical or pharmacological studies originate from the pharmacopeias of the Dusuns, Kadazans, Muruts, Rungus, and Lundayehs. The precise medicinal use of these plants is not clearly documented (Tables 1–6).

### Lycophytes

As surprising as it may be, mosses are a rich source of natural products, including an unusual series of piperidine alkaloids and serene triterpenes (Cao et al. 2017). *Selaginella argentea* (Wall. ex-Hook. & Grev.) Spring (Selaginellaceae) is utilized to treat asthma, body aches, fever, and headaches by the Muruts (Ahmad and Raji 1992; Kulip 2003a) (Table 1). These applications suggest the potential presence of anti-inflammatory or vasoactive compounds, which have yet to be identified (Table 1).

### Monilophytes

The pharmacological properties of ferns, particularly their anti-inflammatory effects, are often due to phenolic compounds (Dion et al. 2015). This is likely the case for *Gleichenia truncata* (Willd.) Spreng. (Gleicheniaceae), which is traditionally used by the Dusun people to treat sore eyes (Kulip 2014) and has shown anti-inflammatory activity *in vivo* (Suhaini et al. 2015). According to the current literature, 11 additional species remain phytochemically or pharmacologically unexplored (Table 2). *Diplazium cordifolium* Bl. (Athyriaceae), used by the Bajaus to treat colds and fever (Wiart 2024), may produce antibacterial or antiviral compounds. Similarly, *Drymoglossum piloselloides* (L.) C. Presl (Polypodiaceae), employed by the Bruneis to treat dysuria and hypertension (Mahmud and Razali 2016), could be a potential source of diuretic compounds. Notably, *Lygodium circinnatum* Sw. (Lygodiaceae), used by the Lundayehs for bacterial infections (Kulip et al. 2000), and *Lygodium salicifolium* C. Presl (Lygodiaceae), also employed by the Lundayehs for viral infections, suggest the presence of antibacterial and antiviral principles, respectively. Some medicinal uses also point to the presence of neuroactive natural products. This is the case for *Drynaria sparsisora* (Desv.) T. Moore (Polypodiaceae) given by the Dusuns and Kadazans for asthma and heart disease (Wiart 2024).

*Stenochlaena palustris* (Burm. f.) Bedd. (Blechnaceae), *Diplazium esculentum* (Retz.) Sw. (Athyriaceae), *Nephrolepis acutifolia* (Desv.) Christ (Nephrolepidaceae), *Acrostichum aureum* L. (Pteridaceae), and *Cyclosorus aridus* (D. Don) Ching (Thelypteridaceae) are considered to be medicinal foods by the Dusuns (Noweg et al. 2003). These species could potentially be used as nutraceuticals. Another interesting candidate is *H. zeylanica*, which is used as a medicinal food by the Dusuns (Noweg et al. 2003) and as a cancer treatment by the Lundayeh community (Kulip et al. 2000; Kulip 2007a).

### Gymnosperms

Gnetaceae are known to contain stilbenes with anti-neuroinflammatory properties (Yan et al. 2024), which may account for the traditional use of *Gnetum macrostachyum* Hook.f. to alleviate fatigue and postpartum conditions by the Bruneis (Table 3) (Kulip 1997). Further studies are warranted.

### Basal angiosperms

This clade is home to plants known to produce isoquinoline alkaloids and lignans of pharmaceutical interest. Fifty-three of the 216 species (or 25.4%) of basal angiosperms identified so far have not been examined for their constituents and pharmacological activities. Out of these 24 species are endemic (Table 4). New phytochemicals can potentially be discovered from rare, understudied plants. Plants in the family Schizandraceae are sources of unusual antiretroviral nortriterpenes and lignans (Gao et al. 2008; Liu et al. 2014). In this family, *Kadsura borneensis* A.C. Sm and *Kadsura lanceolata* King have not been studied. Another example is *E. zwageri*, that is used as a blow-gun dart poison by the Muruts (Kulip 2003a). The toxic principles of this tree are probably neuroactive isoquinoline alkaloids, whose myorelaxant properties could be examined. *Panicum palmifolium* J. Koenig (Poaceae) is taken by both the Dusuns and Kadazans for malaria and might produce antiplasmodial principles (terpenes?). The endemic *Boesenbergia pulchella* (Ridl.) Merr. (Zingiberaceae) could be examined for the presence of anti-SARS-CoV-2 compounds, given that the chalcone panduratin A was identified from *Boesenbergia rotunda* (L.) Mansf. (Zingiberaceae), a plant used in traditional medicine in Thailand (Kanjanasirirat et al. 2020). Antibacterial alkaloids are probably present in *Pycnarrhena tumefacta* Miers (Menispermaceae) used for bacterial skin infections by the Lundayehs.

An interesting aspect of the pharmacopeias of Dusuns, Kadazans, Muruts, and Rungus is the use of food plants unknown to the public. These plants have the potential, if not toxic, to be developed as food supplements or nutraceuticals. Examples are *Plagiostachys albiflora* Ridl. (Zingiberaceae) (Kulip 2007a) and *P. tumefacta* (Noweg et al. 2003).

### Core angiosperms

This clade regroups plants known to produce phenolic compounds, alkaloids and, to a lesser extent, saponins of pharmaceutical interest. Dilleniaceae, Phyllanthaceae, Melastomataceae, Myrtaceae, Combretaceae, and Rubiaceae accumulate tannins and other phenolic compounds, which, through their astringency, are used to stop bleeding, and treat diarrhea, diabetes, and thrush (Lima et al. 2014). Plants accumulating saponins such as in *Archidendron clypearia* (Jack) I.C. Nielsen (Fabaceae) are often employed for hygienic purposes (Voeks and bin Nyawa 2006) or as fish poison, like for instance, in the genus *Barringtonia* (Mangawang et al. 2020).

Sixty plant species in this clade have not been phytochemically or pharmacologically examined. Among these, 13 species are endemic (Table 5). Certain endemic plants within this clade have the potential to contain novel antimicrobial compounds, such as *Millettia nieuwenhuisii* J.J. Smith (Fabaceae) used by the Muruts for thrush (Kulip 2003a) or, *Casearia rugulosa* Bl. (Flacourtiaceae), employed to treat bacterial skin infections by the Dusuns (Voeks and bin Nyawa 2006). Plants in the genus *Shorea* Roxb. ex C.F. Gaertn. (1805) (Dipterocarpaceae) produce stilbenes active against influenza A viruses (Ito et al. 2018). Plants in the genus *Ficus* L. (1753) (Moraceae) yield phenanthroindolizine alkaloids with anti-cancer activity, as identified in *Ficus septica* Burm.f. (Damu et al. 2009). Such alkaloids could also be produced by *Ficus elliptica* Hook ex Miq. (Moraceae). Furthermore, *D. monticola* is traditionally used by the Muruts to prepare poisons for blowgun darts (Wiart 2024). Given that Melastomataceae family members do not typically produce neuroactive alkaloids, an investigation of this plant is warranted. Plants in the family Nepenthaceae are often used to treat diseases linked to infections such as *Nepenthes ampullaria* Jack (Kulip et al. 2000). Antibacterial principles in pitcher plants are naphthoquinones (Babula et al. 2009). *Canarium littorale* Bl. (Burseraceae), *Dacryodes incurvata* (Engl.) H.J. Lam (Burseraceae), and *Nephelium uncinatum* Radlk. ex Leenh. (Sapindaceae) are medicinal foods for the Dusuns. These plants could be examined for their potential value as nutraceuticals.

### Upper angiosperms

In this clade, 34 plant species have not been examined for their active constituents or pharmacological properties, 13 of which are endemic species (Table 6). Some plant species in this clade are interesting candidates for further phytochemical and pharmacological studies. This is the case for *Symplocos odoratissima* Choisy ex Zoll. (Symplocaceae) utilized by the Lundayehs for treating fever and malaria (Kulip et al. 2000). We can also cite *Jasminum bifarium* Wall (Oleaceae), which is used as a remedy for sore eyes among the Lundayehs (Wiart 2024). The Dusuns and Kadazans use a plant in the genus *Kopsia*, locally known as “lodo lodo” (Kulip et al. 2005), which has not yet been identified. This suggests that there are likely more medicinal plants within this family and genus to be discovered in Sabah. In the family *Dischidia rafflesiana* Wall. (Asclepiadaceae) is used for cancer by the Bajaus (Foo et al. 2016).

Rubiaceae are known to produce monoterpene quinoline and indole alkaloids of pharmaceutical interest. Such compounds could be present in species like *N. gigantea*, utilized by the Dusuns for the treatment of thrush (Wiart 2024). Other plants worthy of further investigation are *Psychotria gyrulosa* Stapf (Rubiaceae) employed by the Dusuns to relieve headaches, a symptom of hypertension (Wiart 2024), and *Paederia verticillata* Bl. (Rubiaceae), used by the Dusuns and Kadazans as a vermifuge (Wiart 2024).

Tropane alkaloids in the family Solanaceae and Convolvulaceae are of notable pharmaceutical interest. In this regard, the active principles of plant species like *Merremia gracilis* E.J.F. Campb. & Argent (Convolvulaceae), used to treat asthma, and *Merremia peltata* (L.) Merr. (Convolvulaceae), traditionally used for diarrhea by the Dusuns (Kulip 1997; Kulip 2003b), could also be studied. Additionally, *Embelia dasythyrsa* Miq. (Primulaceae), used for fever and as a medicinal food by the Dusuns (Wiart 2024), could be examined for its potential nutraceutical value.

## Hazards and poisoning

The toxicity and potentially hazardous effects of most of the plants currently identified as being used for medicinal purposes in Sabah remain unknown. However, certain plants, genera, and families are notoriously poisonous and should never be used for self-medication or as components of herbal remedies. The same is true for any plant for which no robust toxicological data exists.

It is not possible to provide estimates of poisoning cases for each group of plants in Sabah. Nor is it possible to provide data on known or suspected incidents, as no records are currently available in the literature.

### Lycophytes

Plants in the genus *Lycopodium* L. (1753) (Lycopodiaceae), are poisonous (Khalid et al. 2019) due to the presence of lycodine-type alkaloids with anticholinesterase activity, such as huperzine A (Felgenhauer et al. 2000).

### Monilophytes

Plants in the genus *Pteris* L. (1753) (Pteridaceae), are poisonous (Shearer 1945). The toxin involved is a norsesquiterpene glucoside, ptaquiloside (Gounalan et al. 1999) acting as a DNA-alkylating agent (Yamada et al. 2007).

### Gymnosperms

*Cycas revoluta* Thunb. (Cycadaceae), is toxic due to the presence of cyanogenetic glycosides (Chang et al. 2004), such as cycasin and neocycasin, which can cause severe vomiting (Chang et al. 2004). In addition, this plant produces non-protein amino acids, such as β-*N*-methylamino-l-alanine, which induces oxidative insults and protein denaturation at the neuronal level, leading to amyotrophic lateral sclerosis (Du et al. 2024).

### Basal angiosperms

Plants in the magnoliids produce poisonous alkaloids. For example, nephrotoxic aristolactam alkaloids, such as aristolochic acid (Chang et al. 2017), are produced by plants in the genus *Aristolochia* Juss. (1789) (Aristolochiaceae). Plants in the family Annonaceae (Neuwinger 1998) and Lauraceae (Kostermans et al. 1969) can induce parkinsonism due to the presence of aporphine alkaloids, such as anonaine, which deplete dopaminergic neurons from dopamine (Matsushige et al. 2012). Bisbenzylisoquinoline alkaloids from Lauraceae, such as d-tubocurarine, are myorelaxant nicotinic receptor antagonists (Wenningmann and Dilger 2001). Plants in the genus *Stephania* Lour. (1790) (Menispermaceae), produce carcinogenic nephrotoxin bisbenzylisoquinoline, such as alkaloids tetrandrine (Xu et al. 2016; Jiang et al. 2020).

In the monocots, *A calamus* is hepatotoxic because it contains the phenylpropanoid β-asarone (Vargas et al. 1998). Araceae often abound with calcium oxalate raphides responsible for nephrotubular obstructions (Yanagawa and Nagasawa 2024). Steroidal saponins in plant the genus *Dracaena* Vand. (1767) (Asparagaceae) are poisonous (Kinghorn 1979). Orchids in the genus *Dendrobium* Sw. (1799) (Orchidaceae) synthesize neurotoxic sesquiterpene alkaloids such as dendrobine (Colombo et al. 2009). The alkaloid dioscorine in the genus *Dioscorea* R.Br. (1810) (Dioscoreaceae) induces fatal paralysis (Bhandari and Kawabata 2005; Sriapha et al. 2015).

### Core angiosperms

Plants in the genus *Begonia* L. (1753) (Begoniaceae) and in the family Cucurbitaceae produce poisonous cucurbitane-type triterpenes such as cucurbitacin E (Colombo et al. 2009). Pyrrolizidine alkaloids, such as usaramine, are responsible for the toxicity of *Crotalaria pallida* Aiton (Fabaceae) (Jaramillo-Hernández et al. 2021; Williams and Molyneux 1987). Non-protein amino acids, such as mimosine, are toxins in *Mimosa pudica* L. (Fabaceae) (Murakoshi et al. 1971) and plants belonging to the family Connaraceae (Bell 2003). Plants in the order Malpighiales Juss, ex Bercht. & J. Presl. (1820) are often poisonous due to the presence of cyanogenic glycosides. This is the case for *Pangium edule* Reinw. (Achariaceae) (Lim and Lim 2013), *Manihot esculenta* Crantz (Euphorbiaceae) (McMahon et al. 1995), and *Passiflora foetida* L. (Passifloraceae) (Andersen et al. 1998).

Fluoroacetic acid is present in the family Dichapetalaceae (Hall 1972). In the family Euphorbiaceae, plants in the genera *Croton* L. (1753) and *Jatropha* L. (1753), as well as *Codiaeum variegatum* (L.) Blume (Euphorbiaceae) and *Pedilanthus tithymaloides* (L.) Poit. (Euphorbiaceae) produce carcinogenic diterpenes, such as 12-*O*-acetylphorbol-13-decanoate (Hecker 1977; Hecker 1981; Salatino et al. 2007). *Sauropus androgynus* (L.) Merr. (Euphorbiaceae) has been linked to obstructive ventilatory impairments, but the toxins involved remain unknown (Lin et al. 1996). *Ricinus communis* L. (Euphorbiaceae) produces the highly toxic lectin ricin (Worbs et al. 2011). *A. toxicaria* produces cardiac glycosides (Bisset 1962), such as malayoside (Shi et al. 2010). Oxaloacetate, present in plants of the family Oxalidaceae, causes tubular obstruction (Wong and Lansing 2021). Despite its popularity and use in local herbal products, *E. longifolia* has been reported to induce prostatic hyperplasia and the toxin involved has not yet unidentified (Faisal et al. 2013).

### Upper angiosperms

*Impatiens balsamina* abounds with calcium oxalate (Capacio and Belonias 2018). In the Rubiaceae, plants in the genus *Psychotria* L. (1759) produce toxic quinoline alkaloids such as emetine (Melo et al. 2021). The fruits of *M. citrifolia* contain high amounts of potassium and when ingested can cause life-threatening hyperkalemia (Mueller et al. 2000). Apocynaceae and Asclepiadaceae are generally poisonous. Some of them produce cardiotoxic cardenolides such as calotropin in *Asclepias curassavica* L. (Asclepiadaceae) (Mishra et al. 2015; Züst et al. 2019). Poisonous iridoid glycosides, such as plumericine, are found in *Allamanda cathartica* L. (Apocynaceae) and *Plumeria acuminata* W.T. Aiton (Apocynaceae) (Chaveerach et al. 2016). Poisonous indole alkaloids, like vincristine from *Catharanthus roseus* (L.) G. Don (Apocynaceae), induce acute cholangitis (Chuah et al. 2024).

Plants in the genus *Strychnos* L. (1753) (Loganiaceae) produce indole alkaloids, such as strychnine, which induce death by paralysis of the diaphragm (Philippe et al. 2004). *Solanum nigrum* L. (Solanaceae) is poisonous due to the presence of steroidal saponins, including solanine (Alexander et al. 1948; Colombo et al. 2009). Other types of toxins in this clade are calystegines indolizidine alkaloids in the Convolvulaceae (Diaz 2015) and hepatotoxic pyrrolizidine alkaloids in the Asteraceae (Wiedenfeld 2011) (Senturk et al. 2021) such as lycopsamine in *Ageratum conyzoides* L. (Asteraceae) (Bosi et al. 2013; Wiedenfeld and Röder 1991). In the family Araliaceae, plants in the genera *Aralia* L. (1753) and *Schefflera* J.R. Forst. & G. Forst. (1776) (Smith 2022) produce toxic saponins (Colombo et al. 2009). Plants in the genus *Pittosporum* Banks ex Gaertn. (1788) (Pittosporaceae) are poisonous, however the toxins responsible have not yet been identified (Colombo et al. 2009).

## Level of study

### General observations

Since the 1970s, a small but gradually increasing number of local studies on medicinal plants in Sabah have been published. However, this field of study is still preliminary and has mostly focused on communities in the western part of Sabah. This focus can be attributed to several factors, including financial constraints such as limited local research funding and insufficient international collaboration. Additionally, the eastern part of Sabah has been impacted by incidents of terrorist kidnappings (Peters et al. 2019). Another significant limiting factor is the excessive bureaucratic red tape at both regional and international levels required to access medicinal plants (Wiart 2024).

### Studies per district

Of the 26 districts in Sabah, eight have yet to be explored in ethnopharmacological studies (Figure 1): Beluran (Tidong, Kadazan, Dusun, and Sungai communities), Kinabatangan (Sungai communities), Lahad Datu (Begahak, Bugis, Ida’an, Illanun, Subpan, and Suluk communities), Kunak (Bajau and Bugis populations), Membakut (Tatana), Putatan (Bajau population), Semporna (Bajau and Suluk populations), and Telupid (Kadazans).

The remaining districts have been the subject of a few studies, which have been mostly published in local journals. Districts with three or more reports available include Keningau (Ahmad and Holdsworth 1994; Kulip 1997; Kulip 2003a; Ahmad and Ismail 2003; Noweg et al. 2003; Muhammed and Muthu 2015; Maid et al. 2017), and Papar (Kulip 1997; Noweg et al. 2003; Ahmad and Ismail 2003; Mahmud and Razali 2016), both in the West, as well as Tambunan (Kulip 1997; Ahmad and Ismail 2003; Noweg et al. 2003; Kulip et al. 2005; Kulip 2014). In the northwest, Kota Belud was subject to three studies (Awang-Kanak et al. 2018; Awang-Kanak et al. 2021; Mahali et al. 2023). In the southwest, Tenom has been explored in works by Ahmad and Holdsworth (1994), Kulip (1997), Noweg et al. (2003), Kulip (2003a), and Nassir and On (2016). Tongod, in the South, was subjected to three studies (Kulip 1997; Kulip et al. 2010; Kulip 2014). In the north, Kudat was covered in studies by Ahmad and Holdsworth (1995), Kulip (1997), Mojiol et al. (2016), Kodoh (2005), Kodoh et al. (2009), Kulip (2009), Kodoh et al. (2017), Kodoh et al., (2021), and Lin (2021, 2022).

Districts with fewer than three reports available include Beaufort (Mojiol et al. 2010), Kota Kinabalu (Andersen et al. 2003; Foo et al. 2016), and Kuala Penyu (Kulip 1997) in the west. Penampang (Kulip 1997), Ranau (Kulip 1997; Kulip et al. 2005), and Tuaran (Ahmad and Ismail 2003; Foo et al. 2018) in the northwest. Sipitang (Kulip 1997; Kulip et al. 2000) in the southeast. Nabawan (Kulip 2003a) and Tawau (Kulip 2003a; Laudeh and Foo 2021) in the southeast. Given this data, it is reasonable to estimate that the total number of medicinal plant species in Sabah significantly exceeds the 696 species documented.

## Comparison with neighboring states and countries

Currently, no comparative studies exist on medicinal plant use across different ethnolinguistic regions of Southeast Asia. Regarding the medicinal flora of Sabah, most of the medicinal plants utilized by ethnic out of the Bornean linguistic group are also used in the Philippines (Quisumbing 1951; Tanalgo et al. 2024), Indonesia (Heyne 1922; Slikkerveer and Slikkerveer 1995; Sundari 2019), Sarawak (Baling et al. 2018), and Malay Peninsula (Wiart et al. 2004). In the Philippines, the department of health has recommended the use of *B. balsamifera*, *Cassia alata* L. (Fabaceae), *P. guajava*, *Momordica charantia* L. (Cucurbitaceae), and *Peperomia pellucida* (L.) Kunth (Piperaceae). The Bonggi-Molbog use *Jatropha curcas* L. (Euphorbiaceae), locally referred to as "tangan-tangan," similarly to some ethnic groups in Zamboanga (Molina et al. 2020). The ancestors of the Bornean linguistic group are believed to have migrated from Taiwan *via* the Philippines during a period when sea levels were low, exposing a landmass known as Sundaland (Sathiamurthy and Voris 2006; Karin et al. 2017), but this theory is being questioned (Bernard Sellato, personal communication). This migration may explain why plants from the primary tropical forests, traditionally used as medicine by ethnic groups within the Bornean linguistic group, are also utilized in the Philippines. This is the case for the Dusuns with *Polyalthia insignis* (Hook.f.) Airy Shaw (Annonaceae) or *Litsea garciae* Vidal (Lauraceae), which raises the question as the Dusuns might have some ancestry from the Philippines as per the “The greater Central Philippines hypothesis” (Blust 1991).

Regarding Indonesia, a potential area of study could involve a comparative analysis of the plants used by the Muruts and the Murut-Tidungs of Kalimantan (Soriente and Inagaki 2012). As for the Bajaus, they use several plants that are also utilized by the Malays of Peninsular Malaysia, such *E. longifolia*, *Labisia pumila* (Bl.) Fern. -Vill. (Primulaceae), *Murraya koenigii* (L.) Spreng. (Rutaceae), and *Citrus hystrix* DC. (Rutaceae).

## Conclusions

Despite some efforts, particularly by local researchers, the body of knowledge regarding Sabah’s medicinal plants remains limited. These studies, however, highlight the use of 696 species of plants, predominantly angiosperms, primarily used for treating infections, digestive issues, injuries, and pains. Many of the plants employed by Dusuns, Kadazans, Muruts, Rungus, and Lundayehs have not undergone thorough phytochemical or pharmacological investigation and out of these 156 species stand out as particularly worthy of further investigation.

In summary, Sabah hosts a rich diversity of medicinal plants with immense potential for developing medicines, herbal remedies, and nutraceuticals of global significance. To unlock this potential, it is crucial to streamline local and international administrative processes, secure adequate research funding, and foster international collaborations, following models successfully implemented in similar regions. However, Sabah’s remaining reservoir of medicinal plants is rapidly diminishing due to deforestation, urbanization, and socio-economic pressures.

The recent COVID-19 pandemic, while relatively mild compared to potential pandemics caused by pan-resistant bacteria, fungi, or emerging zoonotic viruses, underscores the risks posed by deforestation and ecological disruption. Future pandemics are inevitable, and when they arise, new drugs will be urgently needed. At that moment, it will be important to remember that Sabah had the opportunity to harness its medicinal plant resources but failed to act—despite having no justifiable reason not to.

## Data Availability

This review uses data from a variety of sources, including Google Scholar, PubMed, Science Direct, and Web of Science.

## References

[CIT0001] Ahmad FB, Raji H. 1992. Medicinal plants of the Murut community in Sabah. In Ghazzaly I, Siraj O, Murtedza M, editors. Forest biology and conservation in Borneo. Kota Kinabalu: Centre for Borneo Studies.

[CIT0002] Ahmad FB, Holdsworth DK. 1994. Medicinal plants of Sabah, Malaysia, Part II. The Muruts. Int J Pharmacogn. 32(4):378–383. doi: 10.3109/13880209409083019.

[CIT0003] Ahmad FB, Holdsworth DK. 1995. Traditional medicinal plants of Sabah, Malaysia Part III. The Rungus People of Kudat. Int J Pharmacogn. 33(3):262–264. doi: 10.3109/13880209509065377.

[CIT0004] Ahmad FB, Holdsworth DK. 2003. Medicinal plants of Sabah, East Malaysia – Part I. Pharm Biol. 41(5):340–346. doi: 10.1076/phbi.41.5.340.15940.

[CIT0005] Ahmad FB, Ismail G. 2003. Medicinal plants used by Kadazandusun communities around Crocker Range. ASEAN Rev Biodivers Environ Conserv. 1:1–10.

[CIT0006] Alexander RF, Forbes GB, Hawkins ES. 1948. A fatal case of solanine poisoning. Br Med J. 2(4575):518–518. doi: 10.1136/bmj.2.4575.518. PMC2091497 18881287

[CIT0007] Andersen L, Adsersen A, Jaroszewski JW. 1998. Cyanogenesis of *Passiflora foetida*. Phytochem. 47(6):1049–1050. doi: 10.1016/s0031-9422(98)80070-8.

[CIT0008] Andersen J, Nilsson C, de Richelieu T, Fridriksdottir H, Gobilick J, Mertz O, Gausset Q. 2003. Local use of forest products in Kuyongon, Sabah, Malaysia. ARBEC. 2:1–18.

[CIT0009] Appell GN. 1968. The Dusun languages of northern Borneo: the Rungus Dusun and related problems. Ocean Linguist. 7(1):1–15. doi: 10.2307/3622844.

[CIT0010] Arthur HR. 1954. A phytochemical survey of some plants of North Borneo. J Pharm Pharmacol. 6(1):66–72. doi: 10.1111/j.2042-7158.1954.tb10920.x. 13118519

[CIT0011] Awang-Kanak F, Bakar MFA, Mohamed M. 2018. Ethnobotanical survey on plants used as traditional salad food (ulam) in Kampung Taun Gusi, Kota Belud Sabah, Malaysia. AIP Conference Proceedings. 2002, 020024.

[CIT0012] Awang-Kanak F, Matawali A, Jumat NR, Bakri SNS. 2021. A preliminary survey on edibles and medicinal plants used by Dusun of Kampung Pinolobu, Kadamaian, Kota Belud, Sabah, Malaysia. JTBC. 18:21–30. doi: 10.51200/jtbc.v18i.3440.

[CIT0013] Awang-Kanak F. 2022. Commonly available Sabah medicinal plants used for traditional hypertension treatment. In Proceeding of 2nd PPST STEM Seminar 2022. Universiti Malaysia Sabah.

[CIT0014] Awang-Kanak F, Foo J. 2023. Medicinal plants used for traditional skin diseases treatment in Sabah, Malaysia. In Proceeding of 3rd PPST STEM Seminar 2023. Universiti Malaysia Sabah.

[CIT0015] Babula P, Adam V, Havel L, Kizek R. 2009. Noteworthy secondary metabolites naphthoquinones – their occurrence, pharmacological properties and analysis. CPA. 5(1):47–68. doi: 10.2174/157341209787314936.

[CIT0016] Bailey ES, Fieldhouse JK, Choi JY, Gray GC. 2018. A mini review of the zoonotic threat potential of influenza viruses, coronaviruses, adenoviruses, and enteroviruses. Front Public Health. 6:104. doi: 10.3389/fpubh.2018.00104. PMC5900445 29686984

[CIT1016] Baling J, Noweg GT, Sayok AK, Wadell I, Ripen JE. 2018. Medicinal plants usage of Jagoi Bidayuh community, Bau district, Sarawak, Malaysia. JBK. 3(1):67–87. doi: 10.33736/jbk.619.2017.

[CIT0018] Bhandari MR, Kawabata J. 2005. Bitterness and toxicity in wild yam (*Dioscore*a spp.) tubers of Nepal. Plant Foods Hum Nutr. 60(3):129–135. doi: 10.1007/s11130-005-6841-1. 16187016

[CIT0019] Beaman JH. 1998. The plants of Mount Kinabalu, 3: gymnosperms and non-orchid mocotyledons. Kota Kinabalu: natural History Publications (Borneo) in association with Royal Botanic Gardens Kew.

[CIT0020] Bell EA. 2003. Nonprotein amino acids of plants: significance in medicine, nutrition, and agriculture. J Agric Food Chem. 51(10):2854–2865. doi: 10.1021/jf020880w. 12720365

[CIT0021] Benggon CJJ. 2008. Preliminary screening of secondary metabolites from selected medicinal plants from Kg Pulutan, Sabah. Thesis, Faculty of Resource Science and Technology, University Malaysia Sarawak.

[CIT0022] Bisset NG. 1962. Cardiac glycosides: part VII. Planta Med. 10(02):143–151. doi: 10.1055/s-0028-1100286.

[CIT0023] Bosi CF, Rosa DW, Grougnet R, Lemonakis N, Halabalaki M, Skaltsounis AL, Biavatti MW. 2013. Pyrrolizidine alkaloids in medicinal tea of *Ageratum conyzoides*. Rev Bras Farmacogn. 23(3):425–432. doi: 10.1590/S0102-695X2013005000028.

[CIT0024] Blust R. 1991. The greater central Philippines hypothesis. Ocean Linguist. 73–129. https://www.jstor.org/stable/3623084.

[CIT0025] Cao H, Chai TT, Wang X, Morais-Braga MFB, Yang JH, Wong FC, Wang R, Yao H, Cao J, Cornara L, et al. 2017. Phytochemicals from fern species: potential for medicine applications. Phytochem Rev. 16(3):379–440. doi: 10.1007/s11101-016-9488-7. 32214919 PMC7089528

[CIT0026] Capacio AFZ, Belonias BS. 2018. Occurrence and variation of calcium oxalate crystals in selected medicinal plant species. ATR. 40:45–60. doi: 10.32945/atr4024.2018.

[CIT0027] Chang SS, Chan YL, Wu ML, Deng JF, Chiu TF, Chen JC, Wang FL, Tseng CP. 2004. Acute Cycas seed poisoning in Taiwan. J Toxicol Clin Toxicol. 42(1):49–54. doi: 10.1081/CLT-120028744. 15083936

[CIT0028] Chaveerach A, Tanee T, Patarapadungkit N, Khamwachirapithak P, Sudmoon R. 2016. Cytotoxicity and genotoxicity of *Allamanda* and *Plumeria species*. Sci Asia. 42(6):375. doi: 10.2306/scienceasia1513-1874.2016.42.375.

[CIT0029] Collins NM, Sayer JA, Whitmore TC. 1991. Sabah and Sarawak (Eastern Malaysia). In: Collins NM, Sayer JA, Whitmore TC, editors. The conservation atlas of tropical forests Asia and the Pacific. London: Palgrave Macmillan. doi: 10.1007/978-1-349-12030-7_24.

[CIT0030] Colombo ML, Assisi F, Della Puppa T, Moro P, Sesana F, Bissoli M, Borghini R, Perego S, Galasso G, Banfi E, et al. 2009. Exposures and intoxications after herb-induced poisoning: a retrospective hospital-based study. J Pharm Sci Res. 2:123–136.

[CIT0031] Chang SY, Weber EJ, Sidorenko VS, Chapron A, Yeung CK, Gao C, Mao Q, Shen D, Wang J, Rosenquist TA, et al. 2017. Human liver-kidney model elucidates the mechanisms of aristolochic acid nephrotoxicity. JCI Insight. 2(22):e95978. doi: 10.1172/jci.insight.95978. 29202460 PMC5752374

[CIT0033] Chuah YY, Lee YY, Chou CK, Chang LJ. 2024. *Catharanthus roseus* intoxication mimicking acute cholangitis. BMC Complement Med Ther. 24(1):139. doi: 10.1186/s12906-024-04441-1. 38575897 PMC10993546

[CIT0034] Cook FEM. 1995. Economic botany data collection standard. Prepared for the international working group on taxonomic databases for plant sciences. Richmond, UK: Kew Royal Botanic Gardens, Kew.

[CIT0035] Cox PA, Balick MJ. 1994. The ethnobotanical approach to drug discovery. Sci Am. 270(6):82–87. 8023119

[CIT0036] Damu AG, Kuo PC, Shi LS, Li CY, Su CR, Wu TS. 2009. Cytotoxic phenanthroindolizidine alkaloids from the roots of *Ficus septica*. Planta Med. 75(10):1152–1156. doi: 10.1055/s-0029-1185483. 19296431

[CIT0037] De Silva M. 2009. Javanese indentured labourers in British North Borneo. Thesis, University of London. p. 1914–1932.

[CIT0038] De Vienne MS. 2015. Brunei: from the age of commerce to the 21st century. Singapore: NUS Press.

[CIT0039] De Wit HCD. 1948. Short history of the phytography of Malaysian vascular plants. FM. 4:71–161. https://repository.naturalis.nl/pub/532630.

[CIT0041] Diaz GJ. 2015. Toxicosis by plant alkaloids in humans and animals in Colombia. Toxins (Basel). 7(12):5408–5416. doi: 10.3390/toxins7124892. 26690479 PMC4690142

[CIT0042] Dion C, Haug C, Guan H, Ripoll C, Spiteller P, Coussaert A, Boulet E, Schmidt D, Jianbing W, Zhou Y, et al. 2015. Evaluation of the anti-inflammatory and antioxidative potential of four fern species from China intended for use as food supplements. Nat Prod Commun. 597–603. doi: 10.1177/1934578X1501000416. 25973486

[CIT0043] Du Q, Xing N, Guo S, Li R, Meng X, Wang S. 2024. Cycads: a comprehensive review of its botany, traditional uses, phytochemistry, pharmacology and toxicology. Phytochem. 220:114001. doi: 10.1016/j.phytochem.2024.114001. 38286200

[CIT0045] Evans IHN. 1922. Amon primitive people in Borneo. London. Seeley, Service & Co Limited.

[CIT0046] Evans IHN. 1953. The religion of the Tempasuk Dusuns of North Borneo. UK: Cambridge University Press.

[CIT0047] Faisal B, Mustafa NS, Najmuldeen GF, Althunibat OY, Azzubaidi MS. 2013. Histopathological effects of *Eurycoma longifolia* jack extract (Tongkat Ali) on the prostate of rats. J Asian Sci Res. 3:843–851.

[CIT0049] Felgenhauer N, Zilker T, Worek F, Eyer P. 2000. Intoxication with huperzine A, a potent anticholinesterase found in the fir club moss. J Toxicol Clin Toxicol. 38(7):803–808. doi: 10.1081/CLT-100102396. 11192470

[CIT0050] Foo J, Mohamad AL, Omar M, Amir AA. 2016. Ethnobotanical survey of medicinal plants traded at Tamu in Sabah urban area. ATMA. 4:79–87. doi: 10.17576/IMAN-2016-04SI1-09.

[CIT0051] Foo J. 2018. Penglibatan komuniti tempatan dalam pasaran tumbuhan ubatan di tamu pantai barat Sabah. Akademika. 88:35–47.

[CIT0052] Fuller RW, Bokesch HR, Gustafson KR, McKee TC, Cardellina JH, McMahon JB, Cragg GM, Soejarto DD, Boyd MR. 1994. HIV-inhibitory coumarins from latex of the tropical rainforest tree *Calophyllum teysmannii* var. *inophylloide*. Bioorg Med Chem Lett. 4(16):1961–1964. doi: 10.1016/s0960-894x(01)80543-6.

[CIT0054] Fung J. 2004. A comparative study of the Semai and the Muruts Shamanic Cultures. Shaman. 12:85–95.

[CIT0055] Gao XM, Pu JX, Huang SX, Lu Y, Lou LG, Li RT, Xiao WL, Chang Y, Sun HD. 2008. Kadcoccilactones A − J, Triterpenoids from *Kadsura coccinea*. J Nat Prod. 71(7):1182–1188. doi: 10.1021/np800078x. 18590312

[CIT0056] Gin OK. 2015. Borneo in the early modern period: c. late fourteenth to c. late eighteenth centuries. In Early Modern Southeast Asia. 1st ed. New York, USA: Routledge.

[CIT0057] Goh SH, Lee KH, Chuah CH, Ong HC, Madani L, Pereira JT. 1997. A phytochemical study of Borneo: selected plants from Sabah Lowland Forests. J Herbs Spices Med Plants. 5(1):29–52. doi: 10.1300/J044v05n01_05.

[CIT0058] Gounalan S, Somvanshi R, Kumar R, Dash S, Devi V. 1999. Clinico-pathological effects of bracken fern (*Pteridium aquilinum*) feeding in laboratory rats. Indian J Anim Sci. 69:385–388.

[CIT0059] Gunggut H, Mohd D, Zaaba Z, Liu MSM. 2014. Where have all the forests gone? Deforestation in land beneath the wind. Procedia Soc Behav Sci. 153:363–369. doi: 10.1016/j.sbspro.2014.10.069.

[CIT0060] Hall RJ. 1972. The distribution of organic fluorine in some toxic tropical plants. New Phytol. 71(5):855–871. doi: 10.1111/j.1469-8137.1972.tb01965.x.

[CIT0063] Hecker E. 1977. New toxic, irritant and cocarcinogenic diterpene esters from Euphorbiaceae and from Thymelaeaceae. Pure Appl Chem. 49(9):1423–1431. doi: 10.1351/pac197749091423.

[CIT0064] Hecker E. 1981. Cocarcinogenesis and tumor promoters of the diterpene Ester type as possible carcinogenic risk factors. J Cancer Res Clin Oncol. 99(1–2):103–124. doi: 10.1007/bf00412447. 7251629 PMC12253781

[CIT0065] Heyne K. 1922. De nuttige planten van Nederlandsch-Indië tevens synthetische catalogus der verzamelingen van het Museum voor economische botanie te Buitenzorg. Departement Van Landbouw, Nijverheid en Handel. 1

[CIT0067] Ito T, Hayashi K, Nishiguchi M, Hayashi T, Iinuma M. 2018. Resveratrol oligomer C-glucosidesand anti-viral resveratrol tetramers isolated from the stem bark of *Shorea uliginosa*. Phytochem Lett. 28:1–7. doi: 10.1016/j.phytol.2018.07.026.

[CIT0068] Jaramillo-Hernández DA, Rojas DJT, Sánchez DCC, Angulo LCV, Diaz VMM, Castillo LNP, Rodriguez AIR. 2021. Pneumotoxicity and hepatotoxicity due to *Crotalaria pallida* in the subchronic intoxication model in sheep. Rev Investig Vet Perú. 85–162. doi: 10.15381/rivep.v32i6.19923.

[CIT0071] Jiang Y, Liu M, Liu H, Liu S. 2020. A critical review: traditional uses, phytochemistry, pharmacology and toxicology of *Stephania tetrandra* S. Moore (Fen Fang Ji). Phytochem Rev. 19(2):449–489. doi: 10.1007/s11101-020-09673-w. 32336965 PMC7180683

[CIT0072] Julian EA. 1963. British projects and activities in the Philippines: 1759-1805. PhD Thesis, University of London, School of Oriental and African Studies (United Kingdom).

[CIT0073] Kam TS, Subramaniam G, Chen W. 1999. Alkaloids from *Kopsia dasyrachis*. Phytochem. 51(1):159–169. doi: 10.1016/S0031-9422(98)00721-3.

[CIT0074] Kanjanasirirat P, Suksatu A, Manopwisedjaroen S, Munyoo B, Tuchinda P, Jearawuttanakul K, Seemakhan S, Charoensutthivarakul S, Wongtrakoongate P, Rangkasenee N, et al. 2020. High-content screening of Thai medicinal plants reveals *Boesenbergia rotunda* extract and its component Panduratin A as anti-SARS-CoV-2 agents. Sci Rep. 10(1):19963. doi: 10.1038/s41598-020-77003-3. 33203926 PMC7672115

[CIT0075] Karin BR, Das I, Jackman TR, Bauer AM. 2017. Ancient divergence time estimates in *Eutropis rugifera* support the existence of Pleistocene barriers on the exposed Sunda Shelf. PeerJ. 5:e3762. doi: 10.7717/peerj.3762. 29093993 PMC5661453

[CIT0076] Kashman Y, Gustafson KR, Fuller RW, Cardellina JH, McMahon JB, Currens MJ, Buckheit RW, Hughes SH, Cragg GM, Boyd MR. 1992. HIV inhibitory natural products. Part 7. The calanolides, a novel HIV-inhibitory class of coumarin derivatives from the tropical rainforest tree, *Calophyllum lanigerum*. J Med Chem. 35(15):2735–2743. doi: 10.1021/jm00093a004. 1379639

[CIT0077] Kaur A. 2016. Economic change in East Malaysia: Sabah and Sarawak since 1850. In Studies in the economies of East and South-East Asia (SEESEA). London: Springer, Palgrave Macmillan. doi: 10.1057/9780230377097.

[CIT0078] Ken DWT. 2015. The name of Sabah and the sustaining of a new identity in a new nation. Archipel. Études Interdisciplinaires Sur le Monde Insulindien. 89:161–178. doi: 10.4000/archipel.495.

[CIT0079] Khalid M, Narasimhan A, Master M. 2019. Chemical pneumonitis due to inhalation of lycopodium: a case report. ELMCR. 3:57–60. doi: 10.24911/ejmcr/173-1539363917.

[CIT0080] Khan AS, Islam R, Alam MJ, Rahman MM. 2018. Investigation of anti-diabetic properties of ethanol leaf extract of *Bridelia stipularis* L. on alloxan induced type-2 diabetic rats. JAMPS. 18(4):1–9. doi: 10.9734/JAMPS/2018/45396.

[CIT0081] King JK, King JW. 1984. Languages of Sabah, a survey report. Canberra: Dept. of Linguistics, Research School of Pacific Studies. The Australian National University. Serie C – Number. 78.

[CIT00401] Kinghorn DA. 1979. Toxic Plants. US: Columbia University Press.

[CIT0082] Kluge A, Choi JH. 2016. Bonggi language vitality and local interest in language-related efforts: a participatory sociolinguistic study. LDC. 10:548–600.

[CIT0083] Koblenzer PJ. 1958. The health of the Rungus Dusun of British North Borneo. Am J Trop Med Hyg. 61:293–302. 13611809

[CIT0084] Kodoh J. 2005. Surveys of non-timber forest products traded in Tamu, Sabah, Malaysia. Sepilok Bull. 3:27–36.

[CIT0085] Kodoh J, Mojiol AR, Lintangah W. 2009. Some common non-timber forest products traded by indigenous community in Sabah, Malaysia. J Sust Dev. 2:148.

[CIT0086] Kodoh J, Kulip J, Folistinah G, Mojiol AR, Russel A, Awang Besar N. 2014. A Preliminary study on uses of medicinal plants by local communities in Kudat, Sabah, Malaysia. ICNP2014.p. 8–10.

[CIT0087] Kodoh J, Mojiol AR, Lintangah W, Gisiu F, Maid M, Liew KC. 2017. Traditional knowledge on the uses of medicinal plants among the ethnic communities in Kudat, Sabah, Malaysia. Int J Agr Forest Planta. 5:79–85.

[CIT0088] Kodoh J, Dzulkarnin FNA, Hassan A, Maid M. 2021. Marketing procedures and profit: a case study on medicinal plants at selected Tamu (Traditional Market) in Sabah. MJoSHT. 8:627–640.

[CIT0089] Kostermans AJ, Pinkley HV, Stern WL. 1969. A new Amazonian arrow poison: *Ocotea venenosa*. Bot Mus Leafl. 22(7):241–252. doi: 10.5962/p.168371.

[CIT0090] Kroeger PR. 1986. Intelligibility patterns in Sabah and the problem of prediction. In Geraghty P, Carrington L, and Wurm SA, editors, FOCAL I: papers from the Fourth International Conference on Austronesian Linguistics. C-93:309–339. Pacific Linguistics, The Australian National University.

[CIT0091] Kroeger PR. 1991. National language comprehension in rural Sabah. J Mod Lang. 6:29–47.

[CIT0092] Kulip J. 1997. A preliminary survey of traditional medicinal plants in the west coast and interior of Sabah. JTFS. 10:271–274.

[CIT0093] Kulip J, Majawat G, Kulik J. 2000. Medicinal and other useful plants of the Lundayeh community of Sipitang, Sabah, Malaysia. J Trop For Sci. 12:810–816.

[CIT0094] Kulip J. 2003a. An ethnobotanical survey of medicinal and other useful plants of Muruts in Sabah, Malaysia. Telopea. 10(1):81–98. doi: 10.7751/telopea20035608.

[CIT0095] Kulip J. 2003b. Similarity of medicinal plants used by two native communities in Sabah. Paper presented at: III WOCMAP 2003. Proceedings of the Congress on Medicinal and Aromatic Plants; Feb 3; Chiang Mai, Thailand.

[CIT0096] Kulip J, Indu JP, Mision R. 2005. Ethnobotanical survey of medical plants in the village of Kaingaran in Sabah, Malaysia. JTBC. 1:71–77. doi: 10.51200/jtbc.v1i.78.

[CIT0097] Kulip J. 2007a. Common medicinal plants of Sabah. Sepilok Bull. 6:1–23.

[CIT0098] Kulip J. 2007b. Gingers in Sabah and their traditional uses. Sepilok Bull. 7:23–44.

[CIT0099] Kulip J. 2009. Medicinal plants of Sabah, Malaysia: potential for agroforestry. JIRCAS Work Rep. 60:47–48.

[CIT0100] Kulip J, Fan LN, Manshoor N, Julius A, Said IM, Gisil J, Joseph JA, Tukin WF. 2010. Medicinal plants in Maliau Basin, Sabah, Malaysia. JTBC. 6:21–33.

[CIT0101] Kulip J. 2014. The ethnobotany of Dusun people in Tikolod village, Tambunan district, Sabah, Malaysia. Reinwardtia. 14(1):101–121. doi: 10.14203/reinwardtia.v14i1.400.

[CIT0102] Laudeh S, Foo J. 2021. Dokumentasi tumbuhan ubatan mengikut kearifan tempatan Wanita Bugis di Pulau Sebatik, Sabah, Malaysia. MJSSH. 6(12):60–66. doi: 10.47405/mjssh.v6i12.1200.

[CIT0103] Lee YF, Benson CKT, Majalap N, Salegin DJ. 2015. Medicinal plants of Sabah (Volume 1). Sabah Forestry Department. Malaysia: Sabah Government.

[CIT0104] Leong CE. 2009. Lest we forget (security and sovereignty of Sabah). Self-published.

[CIT0105] Liu J, Qi Y, Lai H, Zhang JZ, Jia X, Liu H, Zhang B, Xiao P. 2014. Genus *Kadsura*, a good source with considerable characteristic chemical constituents and potential bioactivities. Phytomedicine. 21(8–9):1092–1097. doi: 10.1016/j.phymed.2014.01.015. 24784528

[CIT0106] Maid M, Tay J, Yahya H, Adnan FI, Kodoh J, Chiang LK. 2017. The reliance of forest community on forest for livelihood: a case of Kampung Wawasan, Sook, Sabah, Malaysia. Int J Agric For Plant. 5:110–118.

[CIT0107] Lim TK, Lim TK. 2013. Pangium edule. In Edible medicinal and non-medicinal plants. Fruits. p. 780–784. New York, US.

[CIT0108] Leonti M. 2022. The relevance of quantitative ethnobotanical indices for ethnopharmacology and ethnobotany. J Ethnopharmacol. 288:115008. doi: 10.1016/j.jep.2022.115008. 35066067

[CIT0109] Lima CC, Lemos L, Conserva LM. 2014. Dilleniaceae family: an overview of its ethnomedicinal uses, biological and phytochemical profile. J Pharmacogn Phytochem. 3(2):181–204.

[CIT0110] Lin TJ, Lu CC, Chen KW, Deng JF. 1996. Outbreak of obstructive ventilatory impairment associated with consumption of *Sauropus androgynus* vegetable. J Toxicol Clin Toxicol. 34(1):1–8. doi: 10.3109/15563659609020224. 8632498

[CIT0111] Lin PYP. 2021. Traditional childbirth practices among the Molbog of Banggi island, Sabah. Borneo Res Bull. 52:128–152. https://eprints.ums.edu.my/id/eprint/40730.

[CIT0112] Lin PYP. 2022. The anthropology of childbirth: A study among the Molbog community of Banggi Island, Sabah, Malaysia. Thesis, Universiti Malaysia Sabah.

[CIT0113] Lobel JW. 2013. Philippine and North Bornean languages: issues in description, subgrouping, and reconstruction. Thesis, University of Hawaii.

[CIT0114] Matsushige A, Kotake Y, Matsunami K, Otsuka H, Ohta S, Takeda Y. 2012. Annonamine, a new aporphine alkaloid from the leaves of *Annona muricata*. Chem Pharm Bull (Tokyo). 60(2):257–259. doi: 10.1248/cpb.60.257. 22293487

[CIT0115] McMahon JM, White WL, Sayre RT. 1995. Cyanogenesis in cassava (*Manihot esculenta* Crantz). J Exp Bot. 46(7):731–741. doi: 10.1093/jxb/46.7.731.

[CIT0116] Mahali SNH, Derak R, Aziz ZA, Tobi B. 2023. Traditional medicinal plants and their uses from Sembirai Village, Kota Belud District, Sabah State, Malaysia Borneo. Biodiversitas. 24(11):5956–5961. doi: 10.13057/biodiv/d241114.

[CIT0117] Mahmud AM, Razali R. 2016. Medicinal plants used by the Brunei community in Kampung Benoni, Papar Sabah. Borneo Akademika. 1:52–61.

[CIT0118] Mangawang J, Cabatan ML, Zante J, Bibon CM. 2020. Phytochemical screening of fish poison tree, *Barringtonia asiatica* seed for potential biopesticidal activity and pharmaceutical uses. CLSU Int J of Sci Technol. 4:58–80. doi: 10.22137/ijst.2020.v4n1.05.

[CIT0119] Martin GJ, Lee Agama A, Beaman JH, Nais J. 2002. Projek etnobotani Kinabalu. The making of a Dusun Ethnoflora (Sabah, Malaysia). 9:p. 1–82.

[CIT0120] Maxwell AR. 1981. The origin of the name “Sabah”. Sabah Soc J. 7:91.

[CIT0122] Melo JKA, Ramos TRR, Baptista Filho LCF, Cruz LV, Wicpolt NS, Fonseca SMC, Mendonça FS. 2021. Poisonous plants for ruminants in the dairy region of Pernambuco, Northeastern Brazil. Pesq Vet Bras. 41:e06807. doi: 10.1590/1678-5150-pvb-6807.

[CIT0123] Mishra AK, George A, Devakiruba NS, Sathyendra S. 2015. A rare case of calotropis poisoning. Indian J Foren Med Toxicol. 9(2):62–64. doi: 10.5958/0973-9130.2015.00074.2.

[CIT0124] Mohiddin YBH, Chin W, Worth DH. 1992. Traditional medicinal plants of Brunei Darussalam Part III. Sengkurong. IJP. 30(2):105–108. doi: 10.3109/13880209209053967.

[CIT0125] Mojiol AR, Adella A, Kodoh J, Lintangah W, Wahab R. 2010. Common medicinal plants species found at burned and unburned areas of Klias peat swamp forest, Beaufort, Sabah Malaysia. JSD. 3(1):109–115. doi: 10.5539/jsd.v3n1p109.

[CIT0126] Mojiol AR, Lintangah W, Musri I, Alamjuri RH, Jaafar CSZ. 2016. Mangroves Forest Produce (MFP): importance and contribution to the local communities at Banggi Island Malaysia using free listing technique. IJAFP. 3:89–94.

[CIT0127] Molina RA, Esperat PEL, Gracia AA. 2020. Traditional healing practices in Zamboanga city, Philippines. IJMR. 6:81–87. doi: 10.36713/epra2013.

[CIT0129] Mueller BA, Scott MK, Sowinski KM, Prag KA. 2000. Noni juice (*Morinda citrifolia*): hidden potential for hyperkalemia? Am J Kidney Dis. 35(2):310–312. doi: 10.1016/s0272-6386(00)70342-8. 10676732

[CIT0130] Muhammed N, Muthu TA. 2015. Indigenous people and their traditional knowledge on tropical plant cultivation and utilization: a case study of Murut communities of Sabah, Borneo. JTRSS. 3(1):117–128. doi: 10.47253/jtrss.v3i1.503.

[CIT0131] Murakoshi I, Ohmiya S, Haguniwa J. 1971. Mimoside: a glucosidic metabolite of mimosine in *Mimosa pudica* and *Leucaena leucocephala*. Chem Pharm Bull. 19:2655–2657.

[CIT0132] Nassir N, On LK. 2016. Penggunaan tumbuh-tumbuhan dalam pengubatan tradisional etnik Murut Tahlol di Sabah: penelitian terhadap unsur kearifan tempatan. Jurnal Gendang Alam. 5:87–106.

[CIT0133] Neuwinger HD. 1998. Alkaloids in arrow poisons. In Alkaloids: biochemistry, ecology, and medicinal applications. Boston, MA: Springer US; p. 45–84.

[CIT0134] Noweg T, Abdullah AR, Nidang D. 2003. Forest plants as vegetables for communities bordering the Crocker Range National Park. ASEAN Rev Biodiv Environ Conser. 1–18. http://www.arbec.com.my/pdf/art3janmar03.pdf.

[CIT0135] On LK, Ishak S. 2016. Beliefs in the Komburongo (*Acorus calamus*) and its spiritual healing among the Dusunic people of Sabah, Malaysia. Adv Sci Lett. 22(5):1336–1339. doi: 10.1166/asl.2016.6606.

[CIT0136] Oppenheimer S. 2009. The great arc of dispersal of modern humans: africa to Australia. Quat Int. 202(1–2):2–13. doi: 10.1016/j.quaint.2008.05.015.

[CIT0137] Peng TN, Li LS, Lian JCK. 2022. Demographic and socioeconomic changes in Sabah. Malaysia: Universiti Malaysia Sabah Press.

[CIT0138] Peters D, Abubakar AU, Dollah R, Hassan WSW. 2019. Holistic development and security for Esszone. Sabah, Malaysia: University Malaysia Sabah Publishing. Kota Kinabalu.

[CIT0139] Philippe G, Angenot L, Tits M, Frédérich M. 2004. About the toxicity of some *Strychnos* species and their alkaloids. Toxicon. 44(4):405–416. doi: 10.1016/j.toxicon.2004.05.006. 15302523

[CIT0140] Poulsen AD, Beaman JH, Beaman RS. 2000. The Plants of Mount Kinabalu. 3. Gymnosperms and non-orchid monocotyledons. Kew Bull. 55(2):501. doi: 10.2307/4115671.

[CIT0141] Prentice DJ. 1969. The Murut languages of Sabah. Australia: The Australian National University Serie C – number 18.

[CIT0142] Pugh-Kitingan J. 2015. Cultural and religious diversity in Sabah and relationships with surrounding areas. Thesis, Tokyo University of Foreign Studies.

[CIT0143] Quisumbing E. 1951. Medicinal plants of the Philippines. Manila: Bureau of Printing.

[CIT0144] Ramdzan AR, Ismail A, Zanib ZM. 2020. Prevalence of malaria and its risk factors in Sabah, Malaysia. Int J Infect Dis. 91:68–72. doi: 10.1016/j.ijid.2019.11.026. 31785400

[CIT0146] Reid A. 1997. Endangered identity: Kadazan or Dusun in Sabah (East Malaysia). J Southeast Asian Stud. 28(1):120–136. doi: 10.1017/S0022463400015204.

[CIT0147] Rho MC, Toyoshima M, Hayashi M, Koyano T, Subramaniam G, Kam TS, Komiyama K. 1999. Reversal of multidrug resistance by kopsiflorine isolated from *Kopsia dasyrachis*. Planta Med. 65(4):307–310. doi: 10.1055/s-1999-13991. 10364833

[CIT0149] Roth HL, Low HB. 1896. The natives of Sarawak and British North Borneo: based chiefly on the mss. of the late HB Low, Sarawak government service. Vol. 1. London, UK: Truslove & Hanson.

[CIT0150] Rutter O. 1922. British North Borneo: an account of its history, resources, and native tribes. London, UK: Dalcassian Publishing Company.

[CIT0151] Rutter O. 1929. The pagans of North Borneo. United States: AMS Press, Inc.

[CIT0152] Salatino A, Salatino MLF, Negri G. 2007. Traditional uses, chemistry and pharmacology of *Croton* species (Euphorbiaceae). J Braz Chem Soc. 18(1):11–33. doi: 10.1590/S0103-50532007000100002.

[CIT0153] Salick J, Biun A, Martin G, Apin L, Beaman R. 1999. Whence useful plants? A direct relationship between biodiversity and useful plants among the Dusun of Mt. Kinabalu. Biodivers Conserv. 8:797–818. doi: 10.1023/A:1008853413930.

[CIT0154] Sathiamurthy E, Voris HK. 2006. Maps of Holocene Sea level transgression and submerged lakes on the Sunda Shelf. Nat Hist J Chulalongkorn Univ. 2:1–44.

[CIT0155] Sharapov AD, Fatykhov RF, Khalymbadzha IA, Zyryanov GV, Chupakhin ON, Tsurkan MV. 2023. Plant coumarins with anti-HIV activity: isolation and mechanisms of action. Int J Mol Sci. 24(3):2839. doi: 10.3390/ijms24032839. 36769163 PMC9917851

[CIT0156] Shi LS, Liao YR, Su MJ, Lee AS, Kuo PC, Damu AG, Kuo SC, Sun HD, Lee KH, Wu TS. 2010. Cardiac glycosides from *Antiaris toxicaria* with potent cardiotonic activity. J Nat Prod. 73(7):1214–1222. doi: 10.1021/np9005212. 20553004 PMC2917517

[CIT0157] Schulz G, Victoria C, Kirschning A, Steinmann E. 2021. Rocaglamide and silvestrol: a long story from anti-tumor to anti-coronavirus compounds. Nat Prod Rep. 38(1):18–23. doi: 10.1039/d0np00024h. 32699874

[CIT0158] Senturk H, Eksin E, Zeybek U, Erdem A. 2021. Detection of senecionine in dietary sources by single-use electrochemical sensor. Micromachines (Basel). 12(12):1585–1585. doi: 10.3390/mi12121585. 34945435 PMC8709324

[CIT0159] Shearer GD. 1945. Some observations on the poisonous properties of bracken (*Pteris aquilina*). J Comp Pathol Ther. 55:301–307. doi: 10.1016/S0368-1742(45)80028-0.

[CIT0160] Slikkerveer LJ, Slikkerveer MKL. 1995. Taman obat keluarga (TOGA): indigenous Indonesian medicine for self-reliance. Practical Action Publishing eBooks.

[CIT0161] Smith JP. 2022. Poisonous plants of home and garden. Botanical Studies. 104

[CIT0162] Sodhi NS, Koh LP, Brook BW, Ng PKL. 2004. Southeast Asian biodiversity: an impending disaster. Trends Ecol Evol. 19(12):654–660. doi: 10.1016/j.tree.2004.09.006. 16701328

[CIT0163] Soejarto DD, Farnsworth NR. 1989. Tropical rain forests: potential source of new drugs? Perspect Biol Med. 32(2):244–256. doi: 10.1353/pbm.1989.0003. 2648321

[CIT0164] Soriente A, Inagaki K. 2012. Kalimantan languages: An overview of current research and documentation. Current Trends of Linguistic Research of Indigenous Languages in Indonesia, p. 1–17.

[CIT0165] Spicak J, Pulkertova A, Kralova-Lesna I, Suchanek P, Vitaskova M, Adamkova V. 2012. Alcoholic chronic pancreatitis and liver cirrhosis: coincidence and differences in lifestyle. Pancreatology. 12(4):311–316. doi: 10.1016/j.pan.2012.05.008. 22898631

[CIT0166] Sriapha C, Tongpoo A, Wongvisavakorn S, Rittilert P, Trakulsrichai S, Srisuma S, Wananukul W. 2015. Plant poisoning in Thailand: a 10-year analysis from Ramathibodi Poison Center. Southeast Asian J Trop Med Public Health. 46(6):1063–1076. 26867365

[CIT0167] Srinivasan K, Muruganandan S, Lal J, Chandra S, Tandan SK, Raviprakash V, Kumar D. 2003. Antinociceptive and antipyretic activities of *Pongamia pinnata* leaves. Phytother Res. 17(3):259–264. doi: 10.1002/ptr.1126. 12672157

[CIT0168] Staub PO, Geck MS, Weckerle CS, Casu L, Leonti M. 2015. Classifying diseases and remedies in ethnomedicine and ethnopharmacology. J Ethnopharmacol. 174:514–519. doi: 10.1016/j.jep.2015.08.051. 26342522

[CIT0169] Stone BC. 1980. The vegetation and plant communities of Pulau Balambangan, Sabah, East Malaysia. JMBRAS. 53(1):68–89.

[CIT0170] Suhaini S, Liew S, Norhaniza J, Lee P, Jualang G, Embi N, Hasidah M. 2015. Anti-malarial and anti-inflammatory effects of *Gleichenia truncata* mediated through inhibition of GSK3β. Trop Biomed. 32(3):419–433. 26695202

[CIT0171] Sundari S. 2019. Ethnobotany study of Dayak society medicinal plants utilization in Uut Murung district. Murung Raya Regency, Central Kalimantan. In IOP Conference Series: Earth and Environmental Science. 298: p. 012005. Indonesia: IOP Publishing.

[CIT0172] Tanalgo K, Plang Y, Dela Cruz K, Rubio M, Hilario-Husain BA, Respicio JM, Lidasan A, Abdullah S, Fabrero GV, Ele RJ, et al. 2024. Patterns and predictors of medicinal plant use among ethnolinguistic groups in the 21st century Philippines. Res Square. doi: 10.21203/rs.3.rs-4413314/v1.

[CIT0173] Ueda T. 2009. Creating ‘Malaysians’: a case study of an urban kampung in Kota Kinabalu, Sabah, Malaysia. In: Kolig E, Angeles VS, Wong S, editors. Identity in crossroad civilisations: ethnicity, nationalism and globalism in Asia. ICAS Publications. Edited, Volume 8. Amsterdam: Amsterdam Univ. Press.

[CIT0174] Van der Ent A. 2011. The ecology of ultramafic areas in Sabah: threats and conservation needs. Gard Bull Singapore. 63:385–394.

[CIT0175] Vargas CP, Wolf LR, Gamm SR, Koontz K. 1998. Getting to the root (*Acorus calamus*) of the problem. J Toxicol Clin Toxicol. 36(3):259–260. doi: 10.3109/15563659809028951. 9656986

[CIT0176] Voeks RA, Nyawa S. 2001. Healing flora of the Brunei Dusun. Borneo Res Bull. 178–196.

[CIT0177] Voeks RA, bin Nyawa S. 2006. Dusun Ethnobotany: forest knowledge and nomenclature in Northern Borneo. J Cult Geography. 23(2):1–31. doi: 10.1080/08873630609478221.

[CIT0178] Voeks RA. 2008. Penan ethnobotany: subsistence strategy and breadth of knowledge. In P.G. Sercombe, B. Sellato (eds), Copenhagen: NIAS Press.

[CIT0179] Wannier MMA. 2017. Early explorers’ fatal destiny in the jungle of Sabah. Warta Geol. 43:63–66.

[CIT0180] Wenningmann I, Dilger JP. 2001. The kinetics of inhibition of nicotinic acetylcholine receptors by (+)-tubocurarine and pancuronium. Mol Pharmacol. 60(4):790–796. doi: 10.1016/S0026-895X(24)12307-3. 11562442

[CIT0181] Wiart C, Mogana S, Khalifah S, Mahan M, Ismail S, Buckle M, Narayana AK, Sulaiman M. 2004. Antimicrobial screening of plants used for traditional medicine in the state of Perak, Peninsular Malaysia. Fitoterapia. 75(1):68–73. doi: 10.1016/j.fitote.2003.07.013. 14693223

[CIT0182] Wiart C. 2024. Medicinal plants of Sabah, North Borneo. CRC Press. New York, USA. doi: 10.1201/9781003402886.

[CIT0183] Wiedenfeld H, Röder E. 1991. Pyrrolizidine alkaloids from *Ageratum conyzoides*. Planta Med. 57(6):578–579. doi: 10.1055/s-2006-960211. 17226207

[CIT0184] Wiedenfeld H. 2011. Plants containing pyrrolizidine alkaloids: toxicity and problems. Food Addit Contam Part A Chem Anal Control Expo Risk Assess. 28(3):282–292. doi: 10.1080/19440049.2010.541288. 21360374

[CIT0185] Williams TR. 1968. Ethnographic research in Northern Borneo. Oceania. 39(1):70–80. doi: 10.1002/j.1834-4461.1968.tb00985.x.

[CIT0186] Williams MC, Molyneux RJ. 1987. Occurrence, concentration, and toxicity of pyrrolizidine alkaloids in *Crotalaria* seeds. Weed Sci. 35(4):476–481. doi: 10.1017/S0043174500060410.

[CIT0187] Wong KW, Lansing MG. 2021. Case of acute kidney injury due to bilimbi fruit ingestion. BMJ Case Rep. 14(7):e242325. doi: 10.1136/bcr-2021-242325. PMC8728385 34301701

[CIT0188] Worbs S, Köhler K, Pauly D, Avondet MA, Schaer M, Dorner MB, Dorner BG. 2011. *Ricinus communis* intoxications in human and veterinary medicine-A summary of real cases. Toxins (Basel). 3(10):1332–1372. doi: 10.3390/toxins3101332. 22069699 PMC3210461

[CIT0189] Wright LR. 1966. Historical notes on the North Borneo dispute. J of Asian Stud. 25(3):471–484. doi: 10.2307/2052002.

[CIT0190] Xu XL, Yang LJ, Jiang JG. 2016. Renal toxic ingredients and their toxicology from traditional Chinese medicine. Expert Opin Drug Metab Toxicol. 12(2):149–159. doi: 10.1517/17425255.2016.1132306. 26670420

[CIT0191] Yamada K, Ojika M, Kigoshi H. 2007. Ptaquiloside, the major toxin of bracken, and related terpene glycosides: chemistry, biology and ecology. Nat Prod Rep. 24(4):798–813. doi: 10.1039/b614160a. 17653360

[CIT0192] Yan Q-W, Su B-J, He S, Liao H-B, Yue-Hou , Wang H-S, Liang D. 2024. Structurally diverse stilbenes from *Gnetum parvifolium* and their anti-neuroinflammatory activities. Bioorg Chem. 143:107060. doi: 10.1016/j.bioorg.2023.107060. 38154389

[CIT0193] Yanagawa Y, Nagasawa H. 2024. *Alocasia odora* poisoning due to calcium oxalate needle crystals in Japan. J Rural Med. 19(3):126–130. doi: 10.2185/jrm.2024-001. 38975041 PMC11222628

[CIT0194] Zimmerman TS, Müller-Eberhard HJ. 1971. Blood coagulation initiation by a complement-mediated pathway. J Exp Med. 134(6):1601–1607. doi: 10.1084/jem.134.6.1601. 5166613 PMC2139102

[CIT0195] Züst T, Petschenka G, Hastings AP, Agrawal AA. 2019. Toxicity of milkweed leaves and latex: chromatographic quantification versus biological activity of cardenolides in 16 *Asclepias* species. J Chem Ecol. 45(1):50–60. doi: 10.1007/s10886-018-1040-3. 30523520

